# Low-spin state design of highly active diatomic catalysts for oxygen reduction reaction

**DOI:** 10.1093/nsr/nwaf490

**Published:** 2025-11-07

**Authors:** Hongguan Li, Zhongbiao Li, Jian Zeng, Zhihao Liu, Shuanlong Di, Xinglong Li, Jing Wang, Shulan Wang, Li Li

**Affiliations:** School of Metallurgy, Northeastern University, Shenyang 110819, China; Foshan Graduate School of Innovation, Northeastern University, Foshan 528311, China; School of Metallurgy, Northeastern University, Shenyang 110819, China; Foshan Graduate School of Innovation, Northeastern University, Foshan 528311, China; School of Metallurgy, Northeastern University, Shenyang 110819, China; Foshan Graduate School of Innovation, Northeastern University, Foshan 528311, China; Department of Chemistry, College of Science, Northeastern University, Shenyang 110819, China; Department of Chemistry, College of Science, Northeastern University, Shenyang 110819, China; Department of Chemistry, College of Science, Northeastern University, Shenyang 110819, China; Key Laboratory of Heavy Metal Deep-Remediation in Water and Resource Reuse, Yanshan University, Qinhuangdao 066004, China; Department of Chemistry, College of Science, Northeastern University, Shenyang 110819, China; School of Metallurgy, Northeastern University, Shenyang 110819, China; Foshan Graduate School of Innovation, Northeastern University, Foshan 528311, China

**Keywords:** low-spin state, oxygen reduction reaction, diatomic catalyst, source-reduction approach, rapid O–O bond cleavage

## Abstract

Fe-based atomic catalysts are widely considered among the most promising non-noble candidates for the oxygen reduction reaction (ORR). The precise manipulation of spin states directly determines their performance but remains highly challenging. Herein, we demonstrate a source-reduction approach to design a low-spin Fe^2+^/Cu–N–C diatomic catalyst with fully occupied *d_z^2^_* orbitals. Compared with conventional Fe^3+^ catalysts, the adsorption energy of the *OH intermediate was significantly lowered by minimizing metal–oxygen orbital interactions. *In situ* synchrotron evidence and *ab initio* molecular dynamics simulations further reveal the unusually rapid O–O bond cleavage for *OOH dissociation that is viewed as another key rate-limiting ORR step. The catalyst therefore exhibited fast ORR kinetics with remarkably high half-wave potentials of 0.926/0.828 V in alkaline/acidic media and superior durability of only 17 mV loss after 10 000 cycles, along with outstanding fuel cell performance. This work provides new insights into the spin state engineering and reaction pathway modulation of catalysts.

## Teaser

This work designs a low-spin diatomic catalyst which exhibits highly efficient and durable oxygen reduction capability, offering a new strategy for fuel cell development.

## INTRODUCTION

As the demand for sustainable energy solutions continues to grow, proton-exchange membrane fuel cells (PEMFCs) have emerged as a leading candidate for sustainable power generation [1–3]. However, their practical performance is largely constrained by the sluggish oxygen reduction reaction (ORR) kinetics at the cathode [4–6]. Single-atom Fe–N–C catalysts have been extensively studied as promising alternatives to platinum-group metals (PGMs) for the cathodic ORR considering their abundant active sites and tunable electronic structure [7–9]. While the well-defined porphyrin-like structures of the FeN_4_ moiety can endow the catalysts with enhanced reactivity and structural regularity, their activity and durability still cannot meet the practical requirements [10–12]. Currently, the rational design of efficient Fe–N–C catalysts, particularly at the atomic and electronic levels, still remains highly challenging.

Theoretical calculations reveal that one of the primary potential-determining steps (PDSs) for ORR is the desorption of the *OH intermediate from the catalytic surface, which is attributed to the localization of the strongly correlated Fe 3d states [13]. Spin modulation has emerged as a promising approach recently to promote the delocalization of Fe 3d charge by enabling spin-related charge transfer between the metal site and key reaction intermediates [14–16]. In Fe–N–C structures, low-spin Fe^2+^ has fully occupied high-energy d orbitals and fewer unpaired electrons, which is considered as the favorable spin state for ORR [17]. Dual-metal sites are also engineered as the diatomic catalysts to promote spin coupling between Fe-metal sites, facilitating spin polarization changes or even spin-state transitions [18–20]. However, the delocalization of Fe 3d charge through previous attempts often induced metallic conductivity, hindering valence state control and leading to a mixture of ferrous and ferric ions [21]. Furthermore, in the in-plane square symmetry of the FeN_4_ moiety in Fe–N–C, Fe^2+^ can exhibit three spin states: intermediate spin (*d_xy_*^2^*d_xz_*^2^*d_yz_*^1^${d}_{{z}^{\mathrm{2}}}$^1^), low spin (*d_xy_*^2^*d_xz_*^2^*d_yz_*^2^), and high spin (*d_xy_*^2^*d_xz_*^1^*d_yz_*^1^*d*_*z*^2^_^1^*d*_*x*^2^__−y^2^_^1^), each presenting different interactions with ORR intermediates due to a difference in spatial orbital occupations [22–24]. Theoretical models have shown that the *d_xz_* (*d_yz_*) and *d_z^2^_* orbitals play a critical role in stabilizing the π* orbitals of O_2_, which are crucial for elevating the ORR performance. However, rational design strategies to achieve favorable orbital occupations, particularly those that stabilize the low-spin configuration of Fe^2+^, are still underdeveloped. Currently, it remains highly challenging to precisely manipulate the spin state of Fe–N–C catalysts without compromising electronic conductivity or structural integrity, highlighting the urgent need for the development of effective low-spin-state regulation strategies.

In this work, we present an elaborately designed Fe source reduction strategy for synthesizing highly active Fe^2+^/Cu–N–C diatomic catalysts. Compared with commonly reported catalysts derived from Fe^3+^, Fe^2+^ offers a fully occupied *d_z^2^_* orbital, which weakens the π interaction with *OH intermediates and promotes efficient decoupling of *OH, thereby rendering *OH desorption no longer the PDS of the ORR process. Mössbauer spectroscopy and synchrotron radiation analyses confirm the presence of a higher proportion of low spin states and accessible active sites in the Fe^2+^/Cu–N–C catalyst over its Fe^3+^ counterpart. We also employed *ab initio* molecular dynamic (AIMD) simulations, original infrared spectroscopy, and *in situ* synchrotron radiation spectroscopy to gain insights into the reaction mechanism under aqueous conditions. The results reveal that the Fe^2+^/Cu–N–C catalyst exhibits a high onset reaction potential and facilitates the rapid cleavage of *OOH intermediates into *O···OH, showcasing its rapid and distinct ORR catalytic pathway. Benefiting from these advantages, the as-synthesized Fe^2+^/Cu–N–C catalyst achieves impressive half-wave potentials in both alkaline and acidic conditions along with exciting peak density of practical fuel cell devices.

## RESULTS AND DISCUSSION

### Diatomic spin state design and microstructure characterization

**Figure 1.**
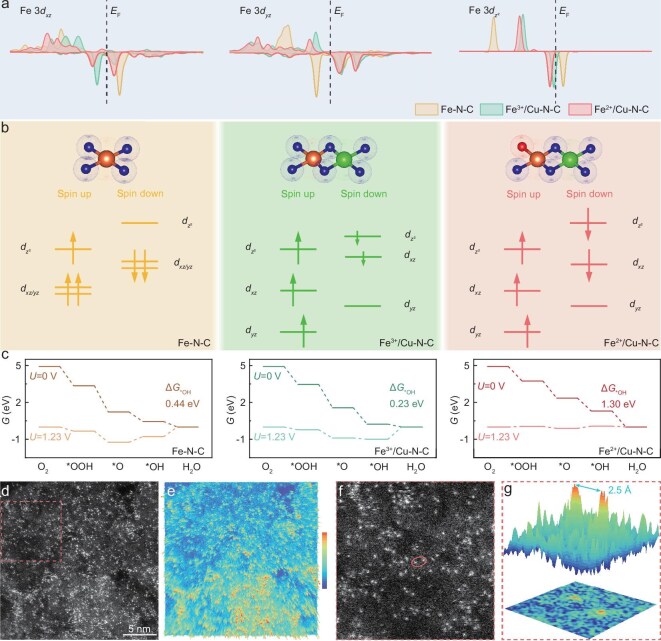
Electronic structure of Fe/Cu diatomic catalysts and their microscopic evidence. (a) PDOS of Fe 3d orbits. (b) Crystal field splitting diagram. (c) Free energy diagrams of Fe–N–C, Fe^3+^/Cu–N–C and Fe^2+^/Cu–N–C, respectively. (d) AC-HAADF-STEM image. (e) The corresponding 3D atom overlapping Gaussian function fitting map of panel (d). (f) The enlarged red region of (d) for highlighting the neighboring pairs. (g) Fe–Cu distance of the observed diatomic pair within the red circle of (f) for Fe^2+^/Cu–N–C.

During the ORR process, the transformation of paramagnetic oxygen molecules into diamagnetic hydroxides or water involves spin-related electron transfer, which is directly influenced by the electronic structure of electrocatalysts. Density functional theory (DFT) calculations were performed for the catalysts to elucidate the detailed correlation between spin-orbitals and their electrocatalytic activity. The projected density of states (PDOS) of the Fe-based catalysts exhibits an asymmetric distribution of spin-up and spin-down electron densities around the Fermi level (Fig. 1a). Among them, Fe–N–C shows a high electron density in the spin-down *d_xz_* and *d_yz_* orbitals, demonstrating its strong interaction between the active Fe sites and adsorbates during ORR with high overpotential [25]. Further differences in spin states of three catalysts (Fe–N–C, Fe^3+^/Cu–N–C and Fe^2+^/Cu–N–C) were revealed through a crystal field splitting diagram, which was used to quantify the adsorption strength (Fig. 1b). With the introduction of Cu atoms adjacent to Fe centers in the Fe–N–C configuration, the local ligand field was distorted due to the sharing of nitrogen atoms between metal atoms, leading to the changes in energy levels of d sub-orbitals and population of Fe 3d spin-down channels. Compared with Fe–N–C, Fe/Cu diatomic sites are more favorable to penetrate the antibonding orbital of O_2_, thus facilitating the spin transition from the triplet-state O_2_ to single-state H_2_O and enhancing ORR kinetics. However, both *d_z^2^_* and *d_xz_* orbitals of Fe^3+^/Cu–N–C were partially occupied, which indicates the generation of strong σ bonding and weak π bonding. The presence of σ bonding further strengthens the interaction between the catalyst and *OH intermediates, which hinders the ORR kinetics and weakens the activity. In contrast, for Fe^2+^/Cu–N–C, the *d_z^2^_* orbital was fully occupied and localized within a narrow energy range, which is stable and would not donate or accept electrons. Therefore, *d_z^2^_* electrons in Fe^2+^/Cu–N–C were not involved in bonding interactions with *OH intermediates, further promoting *OH desorption. The calculated Gibbs free energy diagram (Fig. 1c) reveals that the enhanced σ bonding in Fe^3+^/Cu–N–C causes the PDS of the ORR to be the desorption of *OH, with ΔG_*OH_ = 0.23 eV. In contrast, Fe^2+^/Cu–N–C exhibits a higher *OH desorption energy (ΔG_*OH_ = 1.30 eV), as the fully occupied *d_z^2^_* orbital was unable to interact with *OH, thus promoting *OH desorption and enhancing ORR activity. Based on the above theoretical analysis, Fe and Cu diatomic site catalysts with different spin states were prepared to illustrate the contribution of low-spin orbitals to ORR performance.

The microstructures of the as-prepared catalysts were investigated with X-ray diffraction (XRD) patterns. All catalysts show the amorphous nature of the carbon matrix, with no diffraction peaks associated with metal or metal oxides (Fig. S1) [26]. This result is consistent with Raman spectra analysis, showing characteristic D-band (1344 cm^−1^) and G-band (1590 cm^−1^) features for carbon (Fig. S2). Fe^2+^/Cu–N–C presents a high *I*_D_/*I*_G_ ratio of 1.21, indicating the generation of high content structural defects in the carbon matrix. Nitrogen adsorption/desorption measurements show its high surface area of 1485.30 m^2^ g^−1^ with abundant micropores and mesopores (Figs S3 and S4), demonstrating the presence of highly active catalytic sites for ORR. The high-resolution C 1s spectra of X-ray photoelectron spectroscopy (XPS) confirms the presence of C–N bonds (Fig. S5), indicating that nitrogen atoms were incorporated into the carbon framework. Metal–N bonding structures were also clearly identified within N 1s spectra (Fig. S6), including Fe^2+^/Cu–N–C, Fe–N and Cu–N, demonstrating the binding of metal atoms to the carbon matrix. Aberration-corrected high-angle annular dark-field scanning transmission electron microscopy (AC HAADF-STEM) reveals Fe/Cu diatomic sites, characterized by numerous bright spots, were clearly localized on the carbon support [27], indicating the presence of active sites with high density (Fig. 1d). Additionally, 3D atom overlapping Gaussian-function fitting under different color mappings confirms the even distribution of Fe/Cu diatomic sites throughout the carbon matrix (Fig. 1e). Further analysis (Fig. 1f) demonstrates that most metallic atoms exist in the form of atomic pairs with the proportion of 72 vt% (Fig. S7). As mentioned above, the double exchange interaction between Fe–Cu heteronuclear diatomic sites effectively modulates the charge and spin states of the catalyst, which can significantly influence the ORR activity and selectivity. To validate the interaction between bimetallic sites, the distance between paired Fe–Cu atoms is measured and determined to be within the defined cutoff of 2.5 Å (Fig. 1g).

### Electronic structure characterization of diatomic catalysts

**Figure 2.**
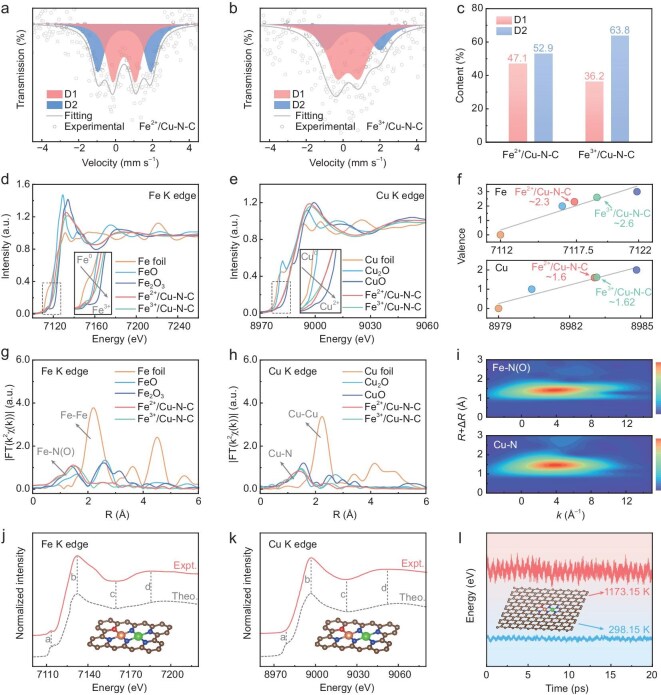
Chemical state and atomic local structure characterization. (a and b) Mössbauer transmission spectra measured at 298 K for Fe^2+^/Cu–N–C and Fe^3+^/Cu–N–C. (c) Comparison of D1 (low/intermediate spin states) and D2 (high spin state) contents for Fe^2+^/Cu–N–C and Fe^3+^/Cu–N–C. (d and e) XANES spectra of the Fe^2+^/Cu–N–C and reference samples for Fe K-edge and Cu K-edge. (f) The fitted average oxidation states of Fe and Cu from XANES spectra. (g and h) FT EXAFS spectra of Fe^2+^/Cu–N–C and reference samples for Fe K-edge and Cu K-edge. (i) WT spectra for Fe^2+^/Cu–N–C at Fe and Cu K-edges. (j and k) Comparison between the experimental K-edge XANES spectra (solid lines) and the theoretical spectra (dashed lines) of Fe and Cu. The brown, blue, red, orange and green balls indicate C, N, O, Fe and Cu atoms, respectively. Vertical dashed lines are provided with typical characteristics labeled at points a–d. (l) AIMD simulations for Fe^2+^/Cu–N–C showing fluctuations of energy at 298.15 and 1173.15 K.

The detailed electronic structure of Fe^2+^/Cu–N–C and Fe^3+^/Cu–N–C were then compared to investigate their difference of electron interactions at atomic sites, which is viewed as one of the critical roles for determining ORR performances. Mössbauer spectroscopy was employed at 298 K to probe the spin states of iron nuclei in Fe–N sites (Fig. 2a and b). The deconvolution of transmission profiles shows the presence of dominant doublets D1 and D2 in both Fe^2+^/Cu–N–C and Fe^3+^/Cu–N–C catalysts, which represent the two differently structured Fe–N moieties of FeN_4_C_12_ and FeN_4_C_10_, respectively [5]. The D1 doublets, characterized by a lower quadrupole splitting (QS) value (∼1.1 mm s^−1^) compared to D2 (1.9–2.6 mm s^−1^), are generally identified as highly active sites for ORR with intermediate or low spin states [11]. A detailed quantitative analysis of the spectra (Fig. 2c) reveals that the D1 site percentage in Fe^2+^/Cu–N–C is 47.1%, significantly higher than in Fe^3+^/Cu–N–C (36.2%). This finding confirms the successful modulation of spin states in our proposed strategy and highlights the potential for enhanced ORR activity in Fe^2+^/Cu–N–C. X-ray absorption spectroscopy (XAS) was then used to further investigate the chemical states and the local coordination environment of catalysts, as it is highly sensitive to weak interactions induced by metal ions [28]. The X-ray absorption near-edge structure (XANES) at Fe K-edge reveals that the absorption edges of Fe^2+^/Cu–N–C and Fe^3+^/Cu–N–C were located between FeO and Fe_2_O_3_, indicating that the Fe valence in both catalysts is between +2 and +3 (Fig. 2d). The highlighted analysis as presented in the insets shows a negative shift in the absorption edge energy of Fe^2+^/Cu–N–C, suggesting its expected lower oxidation state compared to Fe^3+^/Cu–N–C. Notably, the pre-edge peak at ∼7115 eV, corresponding to the 1s to 3d transition [29], indicates that Fe^3+^/Cu–N–C exhibits a centrosymmetric planar *D*_4*h*_ symmetry of the Fe–N_x_ structure [30]. In contrast, the pre-edge peak of Fe^2+^/Cu–N–C shifts slightly to 7113 eV, indicating a departure from the *D*_4*h*_ symmetry. This deviation introduces asymmetry in the Fe–N coordination environment, which enhances the orbital overlap between Fe centers and adsorbed oxygen intermediates, thereby facilitating efficient electron transfer for high ORR kinetics. The corresponding Cu K-edge XANES spectra (Fig. 2e) show Fe^2+^/Cu–N–C and Fe^3+^/Cu–N–C present the same absorption edge energy between Cu_2_O and CuO, suggesting that the valences of Cu species in both catalysts are between +1 and +2. Meanwhile, due to the fully occupied 3d orbitals of Cu, no 1s to 3d transition, as observed for Fe sites, appears in the pre-edge region. The average Fe valence state in Fe^2+^/Cu–N–C is approximately +2.3, which is lower than that of Fe^3+^/Cu–N–C with an average valence of +2.6. The average Cu valence state in both catalysts is nearly identical and unchanged, with an approximate value of +1.6 (Fig. 2f). Consistent with Mössbauer spectroscopy results, the induced lower valence state of Fe in the rationally designed Fe^2+^/Cu–N–C is essential for promoting the adsorption and activation of oxygen intermediates. The low-spin configuration of Fe can also stabilize d orbitals and enhance electronic coupling with oxygen species, promoting efficient charge transfer within catalysts. Therefore, the combined effects between the low valence state and spin-state modulation in Fe^2+^/Cu–N–C can optimize its ORR kinetics for significantly boosting the catalytic performances.

The Fourier transform (FT) of K^2^-weighted χ(k)-function from the extended X-ray absorption fine structure (EXAFS) spectrum at Fe K-edge was then collected (Fig. 2g). The result shows a dominant peak at around 1.5 Å (without phase correction) for Fe^2+^/Cu–N–C, which corresponds to the coordination of the first-shell Fe–N(O) bond, confirming the presence of the Fe–N(O) scattering path. Notably, no peak corresponding to Fe–Fe scattering at 2.2 Å is detected, which further confirms the absence of iron particles in either Fe^2+^/Cu–N–C or Fe^3+^/Cu–N–C. Interestingly, the primary peak of Fe^2+^/Cu–N–C at 1.5 Å exhibits a slight shift relative to Fe^3+^/Cu–N–C, confirming the changes of coordination environment discussed above. Furthermore, quantitative least-squares EXAFS curve fitting analysis reveals that the first-shell fitting requires multiple scattering paths, yielding a lower R-factor (Table S1). The optimized fitting parameters for Fe^2+^/Cu–N–C also demonstrate an average coordination number (CN) of 3 for Fe–N (∼1.95 Å) and a short length of 1 for Fe–O (∼1.87 Å) within the structure, corresponding to an FeN_3_O_1_ configuration, which is different from the common FeN_4_ configuration in Fe^3+^/Cu–N–C and most Fe-related single atomic catalysts. For the EXAFS spectrum of Cu K-edge, the dominant peak at around 1.5 Å that corresponds to the Cu–N scattering path can be found (Fig. 2h). In contrast to the Cu–Cu peak at 2.2 Å for Cu foil, a longer Fe–Cu scattering path at 2.3 Å in Fe^2+^/Cu–N–C confirms the presence of Fe–Cu coordination. The above analysis supports the configuration model of FeN_3_O_1_–CuN_4_ (Figs S8–S17). The wavelet transform (WT) contour plots of EXAFS oscillations at Fe and Cu K-edges can provide detailed insights into both R-space and k-space [31], enabling a high-resolution analysis of the backscattering contributions from Fe–N(O) and Cu–N paths (Fig. 2i). The WT of Fe and Cu in Fe^2+^/Cu–N–C shows an intensity maximum at around 4.0 Å^−1^, corresponding to the dominant scattering paths of Fe–N(O) and Cu–N. Meanwhile, it also demonstrates the absence of Fe–Fe and Cu–Cu scattering, as evidenced by the lack of corresponding signals in reference samples such as Fe and Cu foils, as well as oxides (Figs S18 and S19). To further confirm the local coordination structure of Fe^2+^/Cu–N–C, finite difference method near edge structure (FDMNES) simulations for XANES spectra were then performed using DFT-guided multiple scattering theory for the samples [32,33]. The calculated spectra of both Fe and Cu K-edge show high similarity of peak features to experimental results, particularly for the position and shape of characteristic peaks marked with dotted lines (Fig. 2j and k, Fig. S20). All results shown above confirm the well-defined FeN_3_O_1_–CuN_4_ coordination structures within Fe^2+^/Cu–N–C. Finally, the dynamic stability of the Fe^2+^/Cu–N–C theoretical model is evaluated by AIMD simulation with canonical (NVT) ensemble (Fig. 2l). The results show that the energy oscillates in a small range at the temperatures of 298.15 K and 1173.15 K. Moreover, the catalyst’s structure, particularly the geometry of the active center, remained unchanged after 20 ps, indicating the high thermodynamic stability of Fe^2+^/Cu–N–C.

### ORR activity and fuel cell performances

**Figure 3.**
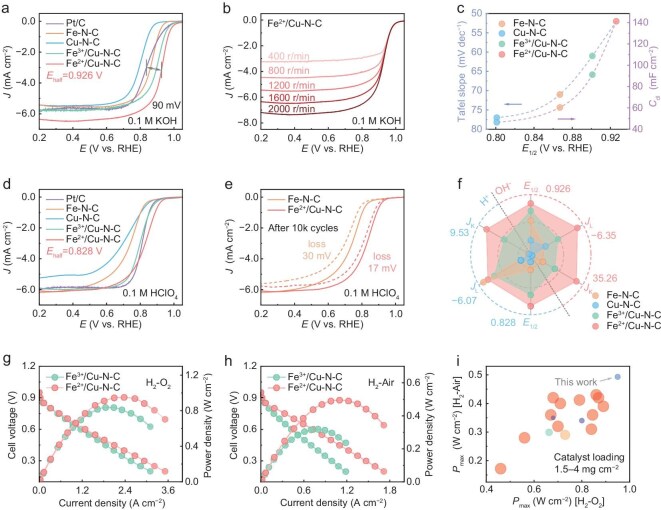
Electrochemical oxygen reduction and PEMFC performance measurements. (a) ORR polarization curves of Fe^2+^/Cu–N–C and reference samples in O_2_-saturated 0.1 M KOH solution. (b) ORR polarization curves of Fe^2+^/Cu–N–C at different rotating rates. (c) The corresponding Tafel slope and *C*_dl_. (d) ORR polarization curves in O_2_-saturated 0.1 M HClO_4_ solution. (e) Stability test with ORR polarization curves before and after 10 000 cycles in O_2_-saturated 0.1 M HClO_4_ solution. (f) The summary of *E*_1/2_, *J*_K_ and limiting current density (*J*_L_) for all samples. (g and h) The polarization and power density curves in H_2_–O_2_ and H_2_-air PEMFCs of Fe^2+^/Cu–N–C and Fe^3+^/Cu–N–C. (i) P_max_ at 1.0 bar of Fe^2+^/Cu–N–C and the literature-reported catalysts in H_2_–O_2_ and H_2_-air PEMFCs.

The ORR performances of the synthesized catalysts and commercial Pt/C as the benchmark were evaluated using the rotating ring-disk electrode (RRDE) measurements with a typical three-electrode configuration in O_2_-saturated 0.1 M KOH solution. Compared with the situation in the N_2_-saturated electrolyte, the cyclic voltammetry (CV) redox peak for Fe^2+^/Cu–N–C in the O_2_-saturated electrolyte appears positively at 0.90 V (Fig. S21), indicating its prominent ORR activity. Linear sweep voltammetry (LSV) polarization curves (Fig. 3a) also exhibit the remarkable onset potential (*E*_onset_) of 1.05 V and half-wave potential (*E_${\frac{1}{2}}$_*) of 0.926 V for Fe^2+^/Cu–N–C, much higher than its peer sample Fe^3+^/Cu–N–C (*E*_onset_: 1.01 V, *E_${\frac{1}{2}}$_*: 0.901 V) and other controls including Fe–N–C (*E*_onset_: 0.965 V, *E_${\frac{1}{2}}$_*: 0.867 V), Cu–N–C (*E*_onset_: 0.905 V, *E_${\frac{1}{2}}$_*: 0.80 V), and commercial Pt/C (20 wt%, *E*_onset_: 1.005 V, *E_${\frac{1}{2}}$_*: 0.837 V). This outstanding ORR performance is among the highest values for all reported M–N–C catalysts (Table S2). Moreover, the ORR pathway was analyzed through LSV curves at various rotating rates and the linear Koutecký–Levich (K–L) plots in the potential range of 0.2–0.6 V confirms the first-order reaction kinetics of Fe^2+^/Cu–N–C (Fig. 3b). The electron transfer numbers (*n* = 4) also confirm its high ORR selectivity in alkaline conditions (Figs S22 and S23). Moreover, Fe^2+^/Cu–N–C exhibited the smallest Tafel slope (52 mV dec^−1^) among all samples, outperforming other catalysts such as Fe^3+^/Cu–N–C (61 mV dec^−1^), Fe–N–C (71 mV dec^−1^), Cu–N–C (77 mV dec^−1^), and Pt/C (111 mV dec^−1^) (Fig. 3c, and Fig. S24a). Fe^2+^/Cu–N–C also achieved the highest kinetic current density (*J*_K_) of 35.26 mA cm^−2^ at 0.85 V, which is 1.9, 3.8 and 34.9 times higher than those of Fe^3+^/Cu–N–C (18.59 mA cm^−2^), Fe–N–C (9.35 mA cm^−2^), and Cu–N–C (1.01 mA cm^−2^), respectively, confirming its highly accelerated ORR process (Fig. S24b). The electrochemically active surface area (ECSA) of Fe^2+^/Cu–N–C shows the highest double-layer capacitance (C_dl_) value of 141.2 mF cm^−2^ for full exposure of electrochemically active sites, surpassing Fe^3+^/Cu–N–C (91.2 mF cm^−2^), Fe–N–C (60.4 mF cm^−2^), and Cu–N–C (46.5 mF cm^−2^) (Fig. 3c, Figs S25 and S26). Further analysis of the LSV curves normalized by the ECSA reveals that the Fe^2+^/Cu–N–C catalyst exhibited higher intrinsic activities than Fe^3+^/Cu–N–C, Fe–N–C and Cu–N–C (Fig. S27). The ORR performance was also evaluated in an O_2_-saturated 0.1 M HClO_4_ acidic electrolyte, which presents a more challenging environment for ORR but is crucial for practical fuel cell applications. Consistent with its performances in the alkaline conditions, Fe^2+^/Cu–N–C demonstrates the highest ORR activity among all catalysts, with the most positive redox peak centered at 0.81 V in CV curves (Fig. S28). The LSV curves further confirm its superior performance with an *E_onset_* of 0.90 V and *E_${\frac{1}{2}}$_* of 0.828 V, which is much higher than those of Fe^3+^/Cu–N–C (*E_${\frac{1}{2}}$_*: 0.80 V), Fe–N–C (*E_${\frac{1}{2}}$_*: 0.738 V), Cu–N–C (*E_${\frac{1}{2}}$_*: 0.718 V) and Pt/C (*E_${\frac{1}{2}}$_*: 0.812 V) (Fig. 3d). This result also far exceeds most of the state-of-the-art atomic catalysts (Table S3). Fe^2+^/Cu–N–C also demonstrates its highest intrinsic activity and rapid reaction kinetics in acidic media with exceptional Tafel slope, *J*_K_ and ECSA values (Figs S29–S34). The subsequent long-term stability test shows that Fe^2+^/Cu–N–C experienced only a 17 mV negative shift of *E*_1/2_, even after 10 000 cycles in 0.1 M HClO_4_, demonstrating its excellent stability with no apparent performance decay (Fig. 3e, Fig. S35). In comparison, Fe–N–C exhibited a 30 mV negative shift. The analysis of *in situ* Raman spectra also shows that no significant fluctuation was observed for D and G bands of Fe^2+^/Cu–N–C, confirming its stabilized carbon structure during ORR (Fig. S36). The comparison of pre- and post-cycling XPS results reveal that the M–N (M=Fe and Cu) bonds of Fe^2+^/Cu–N–C exhibit exceptional stability for the retention of active sites, with only a 2.2% content change, which is much less than those in Fe–N–C (7.4%) and Cu–N–C (4.1%) (Figs S37–S39 and Table S4). Additionally, the inductively coupled plasma mass spectrometry (ICP-MS) analysis reveals that Fe^2+^/Cu–N–C exhibits the lowest metal dissolution into the electrolyte among all samples (Table S5). Overall, the key parameters of ORR are summarized in Fig. 3f to highlight the excellent ORR activity of Fe^2+^/Cu–N–C, with high selectivity and robust stability in both alkaline and acidic media, retaining performance efficiency across pH conditions.

The potential application of the catalysts was further evaluated through the PEMFC tests with membrane electrode assemblies (MEAs) constructed using Fe^2+^/Cu–N–C and Fe^3+^/Cu–N–C as cathode catalysts. The activity of MEAs was investigated under minimal mass transfer resistance with H_2_–O_2_ pressures of 1.0 bar and a catalyst loading of 2.0 mg cm^−2^. The Fe^2+^/Cu–N–C cathode achieved a maximum power density (*P*_max_) of 0.95 W cm^−2^, superior to Fe^3+^/Cu–N–C (0.84 W cm^−2^) (Fig. 3g). Notably, Fe^2+^/Cu–N–C also delivered a high *P*_max_ of 0.49 W cm^−2^ under practical H_2_–air conditions, while the value for Fe^3+^/Cu–N–C is only 0.32 W cm^−2^ (Fig. 3h). In addition, we evaluated the durability test for Fe^2+^/Cu–N–C at a constant voltage of 0.60 V for 100 h. The fuel cell maintained 89.5% of its initial current density, which demonstrates the satisfactory durability of the catalyst under operating conditions (Fig. S40). The MEA performance of Fe^2+^/Cu–N–C in both O_2_ and air under a low catalyst loading surpasses that of most previously reported state-of-the-art catalysts (Fig. 3i and Table S6), highlighting its strong potential for practical use.

### ORR mechanism investigation of diatomic catalysts

**Figure 4.**
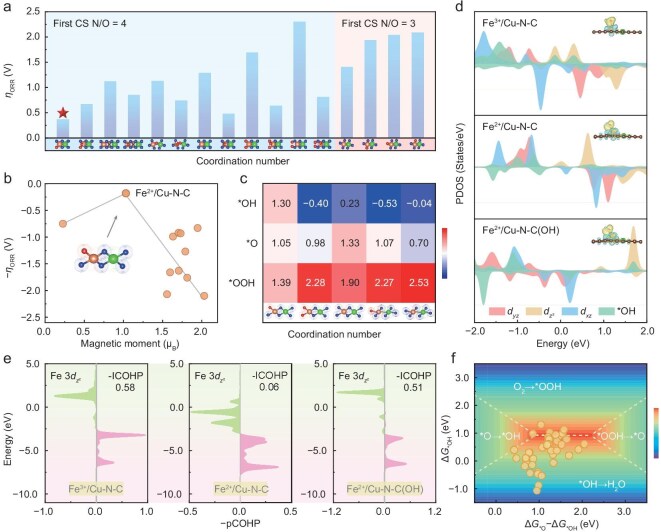
Theoretical mechanism analysis of catalytic activity. (a) Theoretical overpotentials η for Fe/Cu diatomic catalysts with different coordination structures. (b) Volcano plot of negative ORR overpotential with the function of spin moments on the reaction center atoms of Fe/Cu diatomic catalysts. (c) Heat map of coefficient matrix at 0 V among Fe/Cu diatomic catalysts with different coordination structures: FeN_3_O_1_CuN_4_C_10_ (Fe^2+^/Cu–N–C), FeN_2_O_2_CuN_4_C_10_, FeN_4_CuN_4_C_10_ (Fe^3+^/Cu–N–C), FeN_2_O_1_CuN_3_C_10_ and FeN_3_CuN_3_C_10_. (d) The PDOS for Fe 3d orbitals and *OH species of Fe^3+^/Cu–N–C, Fe^2+^/Cu–N–C and Fe^2+^/Cu–N–C(OH), respectively. The inset shows the charge density difference of adsorbed *OH onto the Fe site. Isosurface value is 0.002 e bohr^−3^. (e) COHP plots of Fe 3*d*_*z*^2^_ and O 2p orbitals of *OH on Fe^3+^/Cu–N–C, Fe^2+^/Cu–N–C and Fe^2+^/Cu–N–C(OH) with corresponding ICOHP values listed. (f) The colored contour map of the ORR activity volcano represents Fe/Cu diatomic catalysts as a function of Gibbs free energies.

To probe into the relationship between the spin states of coordination structure and ORR pathway, Fe/Cu diatomic models with different molecular configurations in the diatomic center, the first coordination sphere (CS) and N/O composition were calculated (Figs S41 and S42, Tables S7–S9) with the detailed theoretical overpotential (η) presented in Fig. 4a. As confirmed by the aforementioned structural characterizations, Fe^2+^/Cu–N–C shows the configuration of FeN_3_O_1_CuN_4_C_10_ and its corresponding model exhibits the lowest η of 0.37 V among all Fe/Cu diatomic configurations, which establishes the basis for high ORR performances. Further spin magnetic moment analysis reveals that FeN_3_O_1_CuN_4_C_10_ has a moderate spin magnetic moment (∼1.0 μ_B_), attributed to the introduction of asymmetric FeN_3_O_1_ and CuN_4_ structures, which facilitate charge delocalization, mitigates the strong intermediate adsorption caused by localized charge in symmetric FeN_4_ sites and further reduces the theoretical overpotential of ORR kinetics (Fig. 4b). The calculated Gibbs free energy diagram at equilibrium potential (*U* = 0 V) for FeN_3_O_1_CuN_4_C_10_ also shows ΔG_*OOH_, ΔG_*O_ and ΔG_*OH_ values of 1.39, 1.05 and 1.30 eV (Fig. 4c, and Fig. S43), respectively, indicating that the Δ*G* values of each intermediate are close to the ideal 1.23 eV, which thereby results in a lower theoretical overpotential for the catalyst. Notably, Fe^2+^/Cu–N–C (FeN_3_O_1_CuN_4_C_10_) reveals the step of *O to form *OH as the PDS with the smallest ΔG_*O_ values among the three intermediates, while other Fe–Cu configurations including Fe^3+^/Cu–N–C (FeN_4_CuN_4_C_10_) with the minimum ΔG_*OH_ values suggest the PDS is desorption of *OH to form H_2_O. Specifically, a linear relationship between η and ΔG_*OH_ was established, which suggests that the desorption of *OH is a critical step in reducing the η of Fe/Cu diatomic catalysts (Fig. S44). Furthermore, we also investigate the electronic interaction between the Fe center and the reaction intermediates for Fe^3+^/Cu–N–C (with the model of FeN_4_CuN_4_C_10_) and Fe^2+^/Cu–N–C (FeN_3_O_1_CuN_4_C_10_) along with its configuration with adsorbed OH as the catalyst ligand [FeN_3_O_1_CuN_4_C_10_(OH)] (Fig. 4d, Figs S45–S48). A hybrid state formed near the Fermi level of Fe^3+^/Cu–N–C due to the hybridization between Fe 3*d*_*z*^2^_ and O 2p orbitals was observed, leading to the strong σ-bond formation between Fe and *OH and therefore high energy barrier for ORR kinetics. In contrast, the designed Fe^2+^/Cu–N–C configurations with/without OH ligand exhibit smooth PDOS near the Fermi level with the easy desorption of *OH and thus promoted ORR reaction rates. The projected crystal orbital Hamilton population (COHP) between Fe 3*d*_*z*^2^_ and O 2p orbital of *OH was then calculated to analyze the Fe–O bonding strength (Fig. 4e). Notably, the area of anti-bonding states (green) of Fe^2+^/Cu–N–C presents the largest occupation area, revealing its highest anti-bonding state energy [34,35]. Correspondingly, the integrated COHP (ICOHP) values for Fe–O bonds of Fe^3+^/Cu–N–C, Fe^2+^/Cu–N–C and Fe^2+^/Cu–N–C(OH) are 0.58, 0.06 and 0.51, respectively. This result demonstrates the weaker binding ability of Fe^2+^/Cu–N–C and Fe^2+^/Cu–N–C(OH) than that of Fe^3+^/Cu–N–C, which is consistent with the PDOS result and favorable for *OH desorption. Additionally, the four-electron process of Fe/Cu diatomic catalysts was further deeply plumbed by constructing a 2D active volcano map using the variables ΔG_*O_−ΔG_*OH_ and ΔG_*OH_ (Fig. 4f, Fig. S49) based on the linear relationship between the Gibbs free energy of *OOH versus *OH and *O versus *OH (Fig. S50) [13]. It can be clearly observed that most site data for Fe/Cu diatomic catalysts fall within the *OH → H_2_O region (Fig. 4f), suggesting that the desorption of *OH would be one of the crucial steps for promoting the overall ORR kinetics of the catalysts. Unlike other Fe/Cu diatomic catalysts, Fe^2+^/Cu–N–C breaks the conventional scaling relationship, thereby shifting the PDS from *OH desorption to the *O → *OH conversion process.

### *Operando* XAS analysis of diatomic catalysts

**Figure 5.**
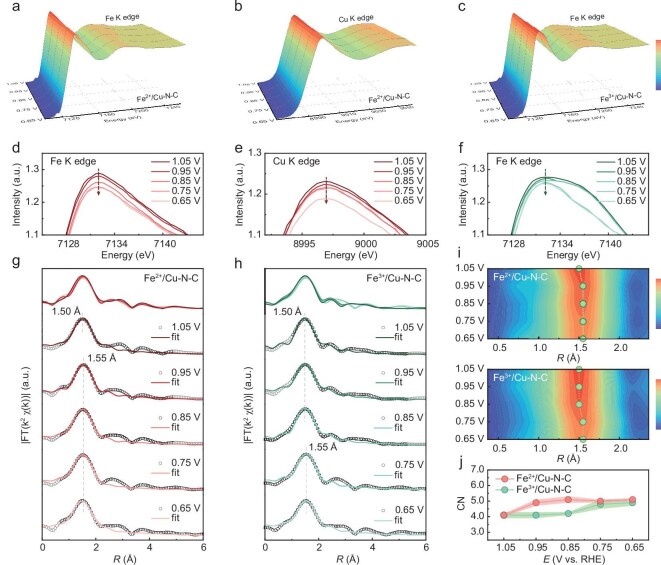
*In situ operando* electrochemical spectroscopy measurements. (a–c) *In situ* XANES spectra changes of Fe K-edge for Fe^2+^/Cu–N–C, Cu K-edge for Fe^2+^/Cu–N–C and Fe K-edge for Fe^3+^/Cu–N–C. (d–f) The corresponding local magnifications of the white-line peaks. (g and h) The first-shell fitting of EXAFS spectra under 1.05–0.65 V versus RHE working conditions for Fe^2+^/Cu–N–C and Fe^3+^/Cu–N–C. (i) The dominant peak counter maps of Fe K-edge EXAFS spectrum for Fe^2+^/Cu–N–C and Fe^3+^/Cu–N–C under 1.05–0.65 V versus RHE. (j) The fitting results of CN.

*Operando* XAFS spectra were recorded with a specially designed electrochemical cell in O_2_-saturated electrolytes to further investigate the dynamic evolution of ligand field profiles and active sites within Fe^2+^/Cu–N–C and Fe^3+^/Cu–N–C catalysts during the ORR process (Fig. S51). The detailed spectra of metal K-edge for Fe^2+^/Cu–N–C and Fe^3+^/Cu–N–C were recorded between 1.05 and 0.65 V versus reversible hydrogen electrode (RHE) (Fig. 5a–c), which visually reflects the remarkable changes in electronic structures of the active sites under ORR conditions. Specifically, the localized magnifications of Fe K-edge XANES spectra for Fe^2+^/Cu–N–C show the intensity increase of the white-line peaks as the oxygen reduction potential shifts negatively, indicating the decrease in the valence state of Fe (Fig. 5d). The apparent intensity changes from the potential of 0.95 to 0.75 V indicate the formation of metastable near-neutral Fe^(2−δ)+^ sites [36], which is beneficial for facilitating ORR kinetics. Its Cu K-edge XANES spectra also show a similar trend for valence state change, demonstrating the successful construction of diatomic active centers for adsorption/desorption of oxygen species during ORR (Fig. 5e). In contrast, the Fe K-edge spectra of Fe^3+^/Cu–N–C show nearly no change in the range of 1.05–0.75 V, which indicates its weak oxygen binding ability and sluggish ORR reaction kinetics (Fig. 5f).

The coordination configuration evolution for Fe sites in catalysts was further investigated using *operando* EXAFS spectra to explore their ORR behavior difference at various potentials [37]. No dominant peak position change (at ∼1.5 Å) is found at 1.05 V from the pristine sample for both Fe^2+^/Cu–N–C and Fe^3+^/Cu–N–C, indicating no adsorption of oxygen intermediates occurs under initial ORR working conditions (Fig. 5g). Subsequently, the dominant peak intensity decreased with the slightly shifted peak center by 0.05 to 1.55 Å as the applied potential decreased from 0.95 to 0.65 V versus RHE for Fe^2+^/Cu–N–C, indicating the onset of oxygen intermediate adsorption and dissociation. The second-shell Fe–N–Cu coordination at 2.8 Å in the FT Fe K-edge EXAFS spectra shifted to 2.6 Å, confirming significant modifications in the local electronic structure and coordination environment. This shift indicates that the induced perturbation propagates through the shared N/C framework of the diatomic sites (Fig. S52) [38]. Further quantitative fitting of FT-Fe K-edge EXAFS spectra reveals the appearance of an additional Fe–O coordination with the CN change from the original 1.0 to 2.0 at 0.95 V (Fig. S53 and Table S10), indicating the adsorption of O species on the catalyst surface and the initiation of the ORR reaction. In contrast, the dominant peak of Fe^3+^/Cu–N–C at 1.5 Å shows no significant change within the applied potential range of 0.95–0.85 V, while the peaks begin to shift at 0.75 V (Fig. 5h). Correspondingly, the fitting results at 0.75 V also confirm the formation of the additional Fe–O coordination which is not seen in the high applied potential (Fig. S54 and Table S11). To visually show the valance state and coordination structure change difference between Fe^2+^/Cu–N–C and Fe^3+^/Cu–N–C, the peak position contour map and corresponding CN change are presented in Fig. 5i and j. Collectively, these results demonstrate the enhanced potential response and accelerated four-electron ORR kinetics of Fe^2+^/Cu–N–C relative to its counterpart.

### *Operando* attenuated total reflectance surface-enhanced infrared absorption spectra (ATR-SEIRAS) study of the solvation-mediated ORR pathway

**Figure 6.**
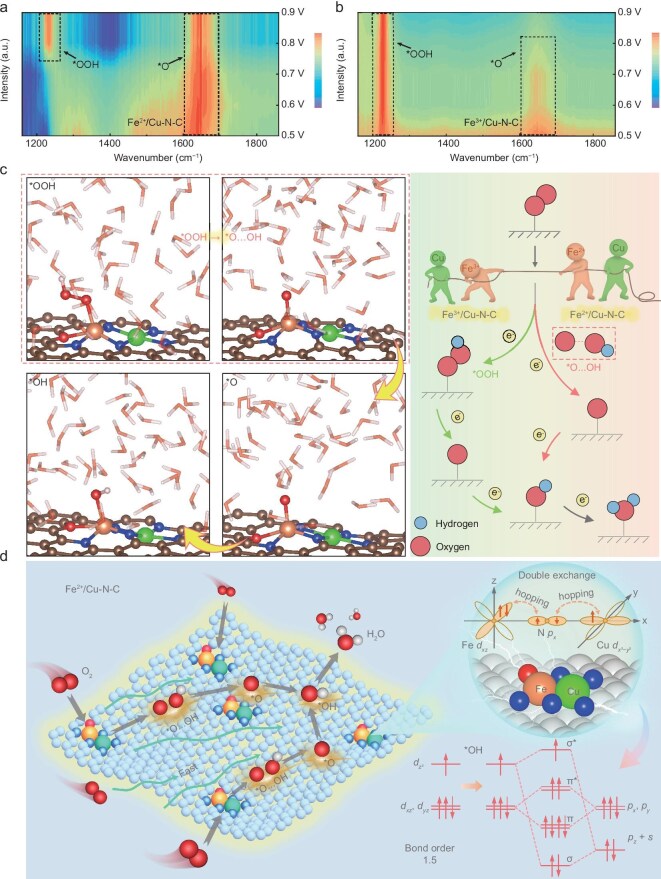
*Operando* ATR-SEIRAS and AIMD snapshots of reaction intermediate transformation and release path. (a and b) *In situ* ATR-SEIRAS spectra recorded in the potential range of 0.5–0.9 V versus RHE for Fe^2+^/Cu–N–C and Fe^3+^/Cu–N–C. (c) *OOH dissociation pathway for the rapid conversion to *O···OH on Fe^2+^/Cu–N–C catalyst. Comparison of the ORR process for Fe^2+^/Cu–N–C (red) and Fe^3+^/Cu–N–C (green) catalysts is listed in the right side. (d) Schematic diagram of ORRs for Fe^2+^/Cu–N–C.

To probe into the detailed reaction mechanism of Fe^2+^/Cu–N–C, we collected *in situ* ATR-SEIRAS at different applied potentials to monitor the change of oxygen intermediate contents [39]. The main absorption bands at 1232 and 1648 cm^−1^ are attributed to the superoxide (*OOH) and adsorbed oxygen atom (*O) intermediates [40,41], respectively (Fig. 6a and b). It can be clearly observed that the *OOH absorption band for Fe^2+^/Cu–N–C gradually disappeared during the negative-going potential steps (0.7–0.5 V versus RHE) accompanied by the increased signal of *O absorption band at 1648 cm^−1^ (Fig. 6a), arising from the destabilization of the O–O bond by the oxygen species bound to Fe sites. This rapid *OOH dissociation pathway in the controlling reactive kinetics region for direct conversion of *O is favorable for the desorption of ORR intermediates. In contrast, Fe^3+^/Cu–N–C did not show the significant changes of *OOH absorption bands through the applied potential from 0.9–0.5 V versus RHE, demonstrating its slow dissociation kinetics on the surface of catalysts (Fig. 6b). Correspondingly, the absorption band of *O species become even stronger. The ATR-SEIRAS analysis confirms the promoted ORR reaction kinetics of Fe^2+^/Cu–N–C over its counterparts could be attributed to the rapid dissociation of *OOH intermediate induced by the tuned electronic states of Fe^2+^ center.

The AIMD simulations were also conducted for further study of the dynamic behavior of the electrochemical interface of Fe^2+^/Cu–N–C on a larger spatial scale during the ORR in aqueous solution (Fig. 6c). A detailed new dissociation mechanism of *OOH, which rapidly releases the hydroxide anion on the surface of Fe^2+^/Cu–N–C, is illustrated. It is observed in the AIMD simulations that the *OOH species is unstable on Fe^2+^/Cu–N–C and solvated by surrounding water molecules, which weakens the O–O bond. This observation is consistent with thermodynamic calculations, in which the ΔG_*OOH_ values (1.39 eV in vacuum and 1.67 eV with solvation) are close to the equilibrium potential of 1.23 eV. The small deviation suggests that *OOH dissociation requires only a minimal external energy input. Unlike the traditional ORR working mechanism that follows *O_2_ → *OOH → *O → *OH → H_2_O, in which *OOH dissociation is the one of the primary rate-limiting steps with high energy barrier, herein *OOH follows the first proton-coupled electron transfer (PCET) step to form *O···OH [OH pseudo-adsorbed with oxygen species (*O)] and then rapidly converted to *O [42]. In other words, the typically sluggish *OOH dissociation step in the conventional ORR pathway is effectively bypassed in our Fe^2+^/Cu–N–C catalyst through the rapid formation of *O···OH intermediates, which promotes O–O bond cleavage and remarkedly enhances the overall ORR kinetics (Fig. 6d). The double exchange interaction occurring through the Fe–N–Cu bridge whereby the spin-down Fe *d_xz_* electrons are allowed to hop through N to the empty orbitals of Cu. The spin conservation is maintained during the process, which strengthens the interaction between the metal sites and N atoms, as evidenced by the broadened Fe *d_xz_* orbitals in the PDOS. This effect helps mitigate demetallation and contributes positively to enhancing the durability of the catalyst. Meanwhile, the calculated bond order of *OH adsorbed on Fe^2+^ (1.5) is notably lower than that on Fe^3+^ (2.0), indicating a weaker binding strength and thereby facilitating *OH desorption—another critical step in optimizing ORR kinetics. In conjunction with the promoted *OOH dissociation pathway, these findings suggest that Fe^2+^/Cu–N–C effectively modulates both the transformation and release of key intermediates. This dual regulation significantly reduces the overall reaction energy barrier, thereby accelerating the oxygen reduction process.

## CONCLUSION

In summary, a low-spin Fe^2+^/Cu–N–C dual-metal catalyst with fully occupied *d_z^2^_* orbitals was successfully developed by controlling the valence state of the Fe precursor. *Operando* XAFS and ATR-SEIRAS analyses reveal that the designed catalyst initiates the oxygen reduction reaction at a higher potential and follows a distinct reaction pathway compared with the commonly reported Fe^3+^ peer counterpart. Unlike the conventional four-electron route (*O_2_ → *OOH → *O → *OH → H_2_O), in which *OOH dissociation is one of the most challenging steps, the Fe^2+^/Cu–N–C catalyst enables rapid O–O bond cleavage of *OOH via the formation of the *O···OH intermediate, which readily transforms into *O accompanied by easy *OH desorption, thereby significantly accelerating the overall ORR kinetics. The catalyst therefore exhibits impressive ORR activity and durability, with half-wave potentials of 0.926 V in alkaline and 0.828 V in acidic media, and a minimal degradation of only 17 mV after 10 000 cycles. Furthermore, it also achieves an exceptional peak power density of 0.95 W cm^−2^ in PEMFC tests. This work not only provides new mechanistic insights into ORR catalysis but also offers an effective strategy for the rational design of high-performance electrocatalysts.

## Supplementary Material

nwaf490_Supplemental_File
