# Supplementary material for: Low-spin state design of highly active diatomic catalysts for oxygen reduction reaction
Source: Natl Sci Rev. 2025 Nov 7;13(2):nwaf490. doi: 10.1093/nsr/nwaf490 (PMC12839530; doi:10.1093/nsr/nwaf490)
Supplement: nwaf490_Supplemental_File [file nwaf490_supplemental_file.pdf]

Supplementary Information

Low-Spin State Design of Highly Active Diatomic Catalysts for Oxygen Reduction Reaction

Hongguan Li[1,2], Zhongbiao Li[1,2], Jian Zeng[1,2], Zhihao Liu[3], Shuanlong Di[3], Xinglong Li[3], Jing Wang[4,*], Shulan Wang[3], Li Li[1,2,*]

[1]School of Metallurgy, Northeastern University, Shenyang 110819, China.

[2]Foshan Graduate School of Innovation, Northeastern University, Foshan 528311, China.

[3]Department of Chemistry, College of Science, Northeastern University, Shenyang 110819, China.

[4]State Key Laboratory of Metastable Materials Science and Technology, Key Laboratory of Heavy Metal Deep-Remediation in Water and Resource Reuse, Yanshan University, Qinhuangdao 066004, China.

[*]Corresponding authors. E-mail: jwang6027@ysu.edu.cn; lilicmu@alumni.cmu.edu

This file includes:

Materials and Methods

Figures S1-S54

Tables S1-S11

References (1-49)

Materials

Ferric chloride anhydrous ($FeCl_3$, ≥99.9%), copper nitrate trihydrate ($Cu(NO_3)_2 \cdot 3H_2O$, ≥99.9%), chitosan ($(C_6H_{11}NO_4)_n$, deacetylated ≥95%), ammonium chloride (NH_4Cl, ≥99.5%) and potassium hydroxide (KOH ≥85%) were purchased from Shanghai Aladdin Biochemical Technology Co., Ltd. Hydrochloric acid (HCl 37%) was purchased from Xi'an Sanpu Chemical Reagent Co., Ltd. Perchloric acid ($HClO_4$ 70%) and Nafion solution (5.0 wt%) were purchased from Sigma-Aldrich. Commercial Pt/C catalyst (20 wt% Pt on Vulcan XC-72 carbon) was purchased from Johnson Matthey. Deionized water was used in all experiments.

Methods

Synthesis of Fe^{2+}/Cu-N-C

Firstly, 0.15 g ferric chloride anhydrous was dissolved in 25 mL of deionized water with 200 μL hydrochloric acid added for pH adjustment. Two copper foil pieces (16 mm in diameter) were then immersed into the solution with stirring for 20 minutes until its color change from yellow to light green. 0.8 g pretreated chitosan and 1 g ammonium chloride were then added. After thorough stirring, the copper foil was removed and the mixture was rapidly freeze-dried. The resulting powder was uniformly ground and carbonized in a nitrogen atmosphere at 750 ℃ for 4 hours with a heating rate of 3 ℃ min^{-1}. After carbonization, the sample was acid-washed to remove metal oxides and carbides formed during synthesis following with water washing to neutralize the pH value to 7. After suction filtration and drying, the product was annealed at 900 ℃ for 2 hours under N_2 with a heating rate of 5 ℃ min^{-1}. The final product was designated as Fe^{2+}/Cu-N-C.

Synthesis of Fe^{3+}/Cu-N-C

0.15 g ferric chloride anhydrous and 0.2 g copper nitrate trihydrate were dissolved in 25 mL of deionized water following with the addition of 0.8 g pretreated chitosan and

1 g ammonium chloride. The solution was then stirred evenly and freeze-dried to obtain the powder. Its subsequent pyrolysis, acid etching and annealing processes were same with those of Fe^{2+}/Cu-N-C and the final obtained product was denoted as Fe^{3+}/Cu-N-C.

Synthesis of Fe-N-C and Cu-N-C

The control samples Fe-N-C and Cu-N-C were prepared with a similar process to that used for Fe^{3+}/Cu-N-C. The only difference is the precursor used by excluding copper nitrate trihydrate for Fe-N-C and ferric chloride anhydrous for Cu-N-C.

Materials characterization

AC HAADF-STEM images were recorded on the aberration-corrected FEI Titan Themis operating at 200 kV. XRD patterns were recorded on a Bruker D8 Advance with Cu Kα radiation. ATR-SEIRAS were collected using KBr pellets as support on a Bruker VERTEX 70v spectrometer. Raman spectra were recorded on a HORIBA LabRAM HR Evolution spectrometer with a 633 nm laser excitation. XPS measurements were collected on a Thermo Fisher Scientific using a monochromatic Al Kα X-ray beam. The Brunauer-Emmett-Teller (BET) and Barrett-Joyner-Halenda (BJH) methods were used to measure specific surface area. The metal contents in the electrolytes were determined using ICP-MS with Agilent 7800. Mössbauer spectra were recorded by using a Topologic 500A spectrometer and a proportional counter. The velocity was calibrated with a standard α-iron foil. The spectra were fitted with the appropriate superpositions of Lorentzian lines using the MössWinn 3.0i software. XAS were collected by employing at 1W1B beamlines of the Beijing Synchrotron Radiation Facility (BSRF), which operated at 2.5 GeV with a maximum current of 250 mA. The data were collected using a 19-element Ge solid-state detector.

Electrochemical measurements

The performance and stability of catalysts were evaluated by Shanghai Chenhua

CHI760E electrochemical workstation in a three-electrode configuration. The three electrodes consisted of a working electrode (RRDE, PINE), reference electrodes (Hg/HgO for alkaline conditions and Ag/AgCl for acidic conditions), and a counter electrode (Pt foil). Catalyst ink was prepared by dispersing 5 mg sample in 1 mL of a solution composed of ethanol (700 μL), isopropyl alcohol (280 μL) and Nafion (20 μL). The mixture was ultrasonically dispersed and then drop-cast onto a glassy carbon electrode. The electrolyte was saturated with N_2 and O_2 prior to testing. CV and LSV curves were obtained in the potential interval (0.3 ~ −0.8 V for alkaline media and 1 ~ −0.25 V for acidic media) with a sweep rate of 50 and 10 mV s^{-1}, respectively. The working electrode scans at a speed of 10 mv^{-1} and rotates at speeds ranging from 400 rpm to 2000 rpm. According to the Koutecky-Levich equation [1], the number of transferred electrons is calculated using the slope of linear fit lines.

Computational method

DFT calculations were performed using Vienna Ab Initio Package (VASP) within the projector augmented wave (PAW) method [2-4]. The generalized gradient approximation (GGA) Perdew-Burke-Ernzerhof (PBE) functional for the exchange correction potential were used to describe core-valence interactions [5]. A 6×6 graphene supercell and Fe, Cu were employed as atomic sites to model the catalyst structure. A vacuum of 20 Å along c-axis was utilized to prevent interlayer interactions. The cut-off energy was set to be 650 eV. For structure optimization in the Brillouin zone, the Monkhorst-Pack method with a 3×3×1 K-point grid was applied, while a 7×7×1 K-point grid was used for property calculation. The energy convergence criterion was 10^{-5} eV, and the force certification is at 0.01 eV $Å^{-1}$. Van der Waals interaction is studied using the semi-empirical DFT-D2 field method.

The AIMD simulations were performed using the CP2K package and sampled by the canonical (NVT) ensemble employing Nose-Hoover thermostats with a time step

of 1.0 fs at a finite temperature of 298.15 and 1173.15 K for more than 20 ps [6]. The Goedecker-Teter-Hutter (GTH) pseudopotentials were chosen for describing core electrons. The wave functions were expanded in optimized double-ζ Gaussian basis sets. A box containing 310 H_2O molecular was used to simulate the solid-liquid interface.

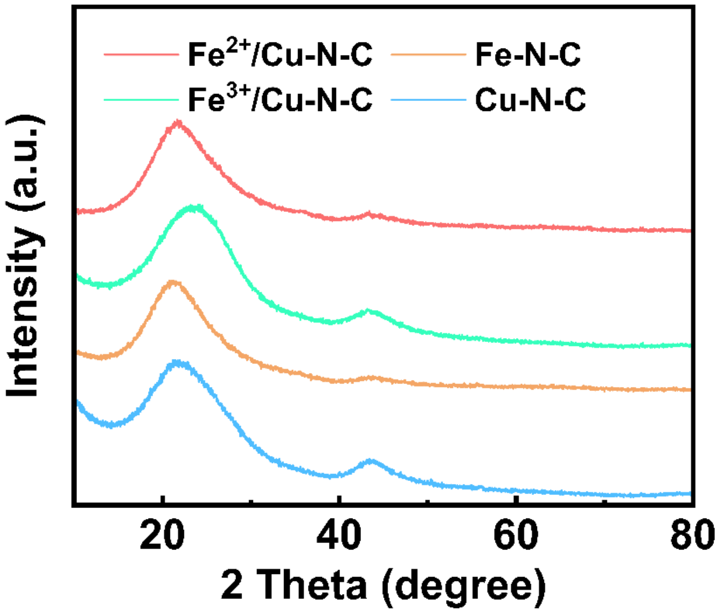

Fig. S1: XRD patterns of Fe^{2+}/Cu-N-C, Fe^{3+}/Cu-N-C, Fe-N-C and Cu-N-C.

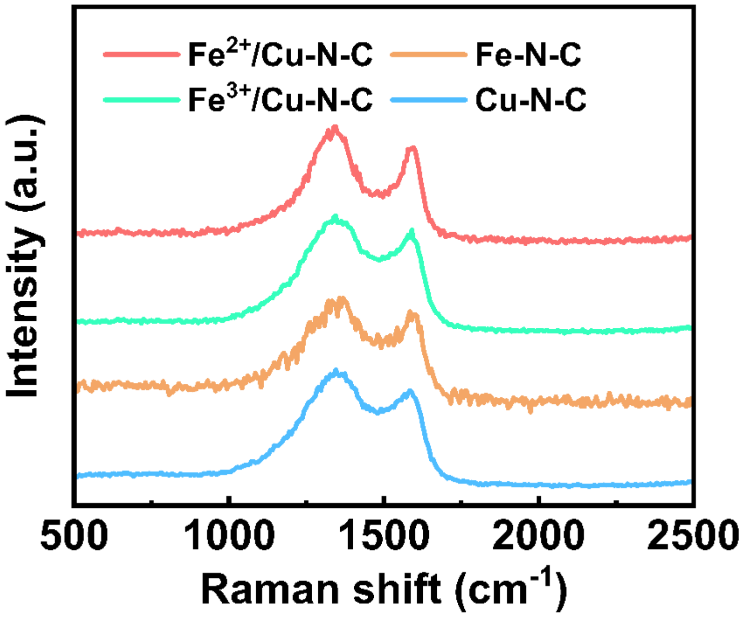

Fig. S2: Raman spectra of Fe^{2+}/Cu-N-C, Fe^{3+}/Cu-N-C, Fe-N-C and Cu-N-C.

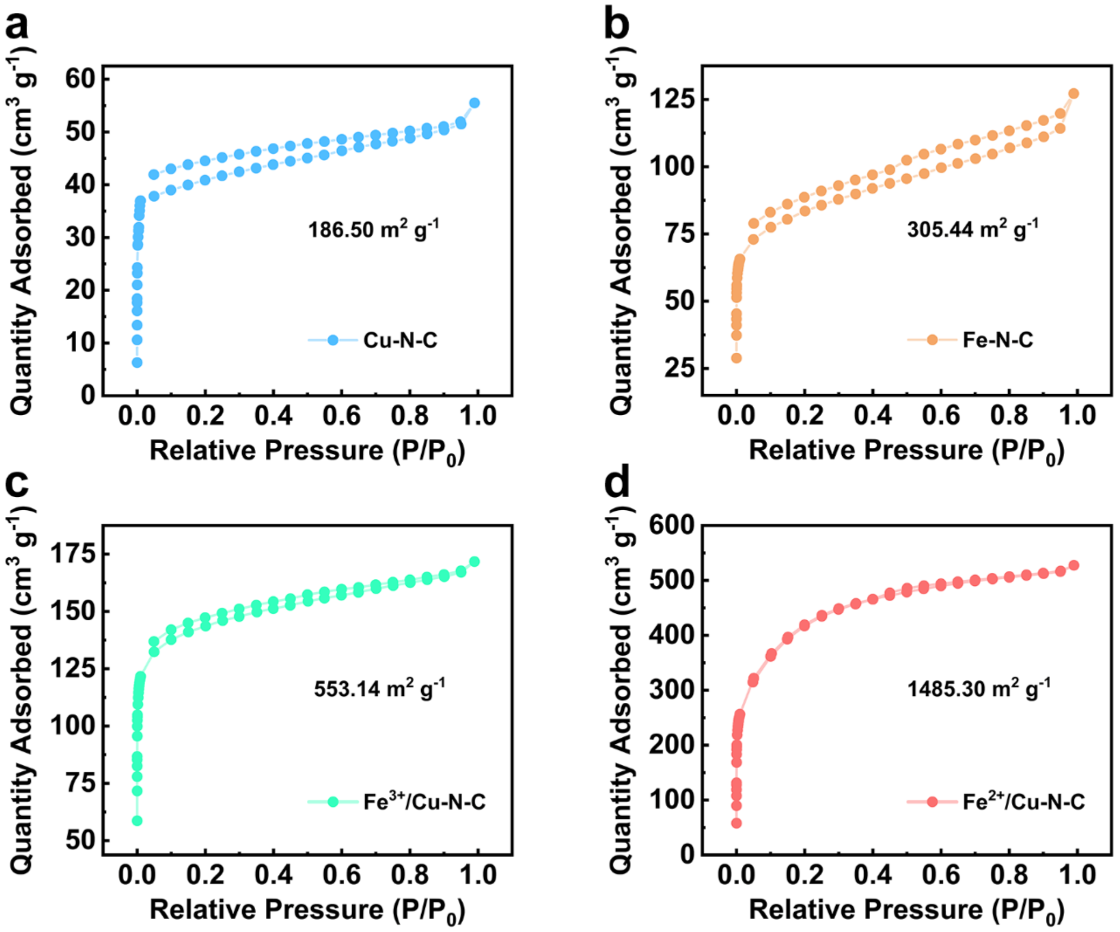

Fig. S3: N_2 adsorption/desorption isotherms of (a) Cu-N-C, (b) Fe-N-C, (c) Fe^{3+}/Cu-N-C and (d) Fe^{2+}/Cu-N-C.

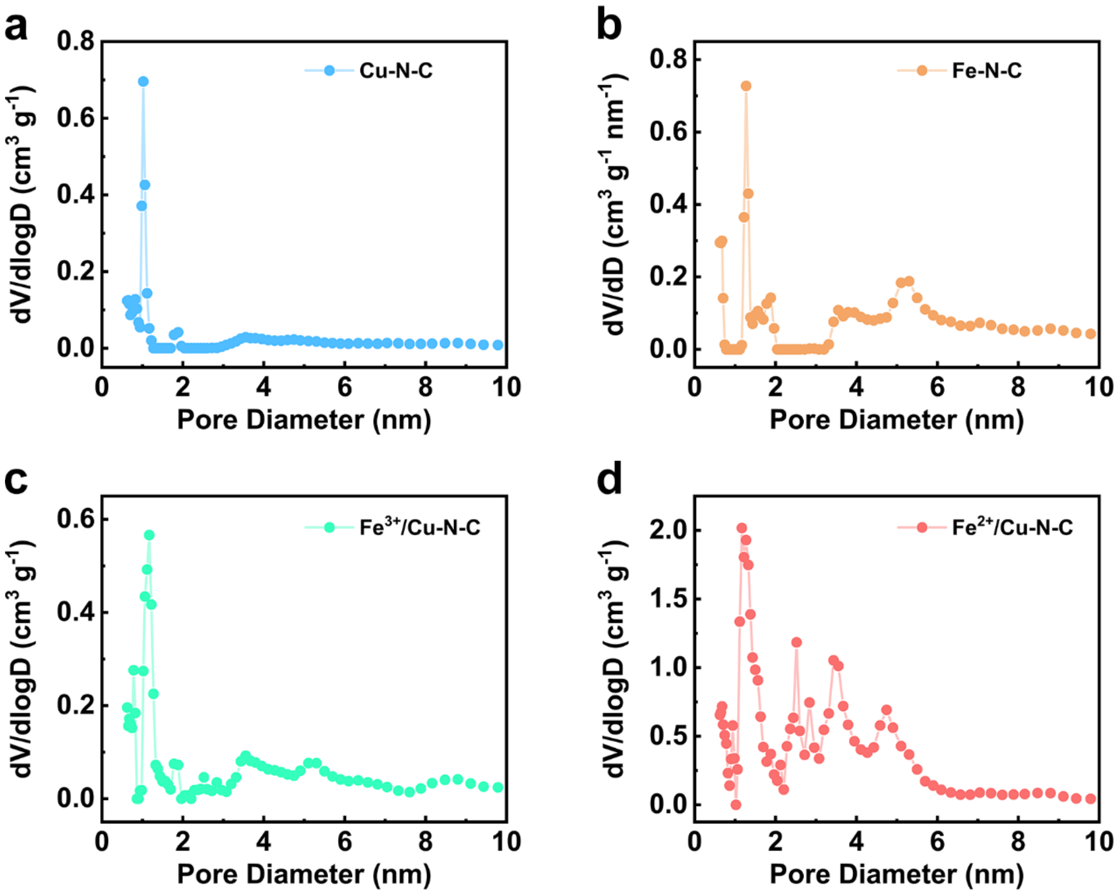

Fig. S4: Pore size distribution of (a) Cu-N-C, (b) Fe-N-C, (c) Fe^{3+}/Cu-N-C and (d) Fe^{2+}/Cu-N-C.

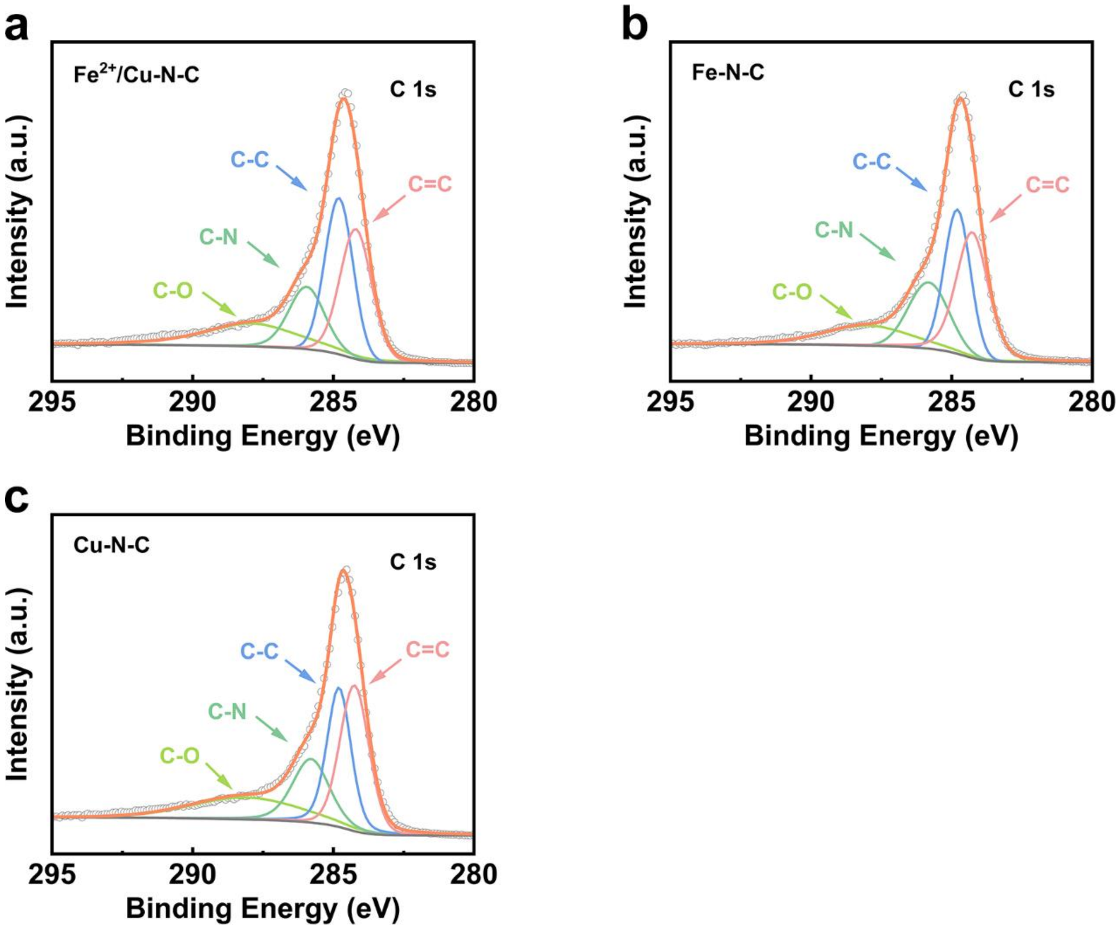

Fig. S5: High-resolution XPS spectra of C 1s for (a) Fe^{2+}/Cu-N-C, (b) Fe-N-C and (c) Cu-N-C.

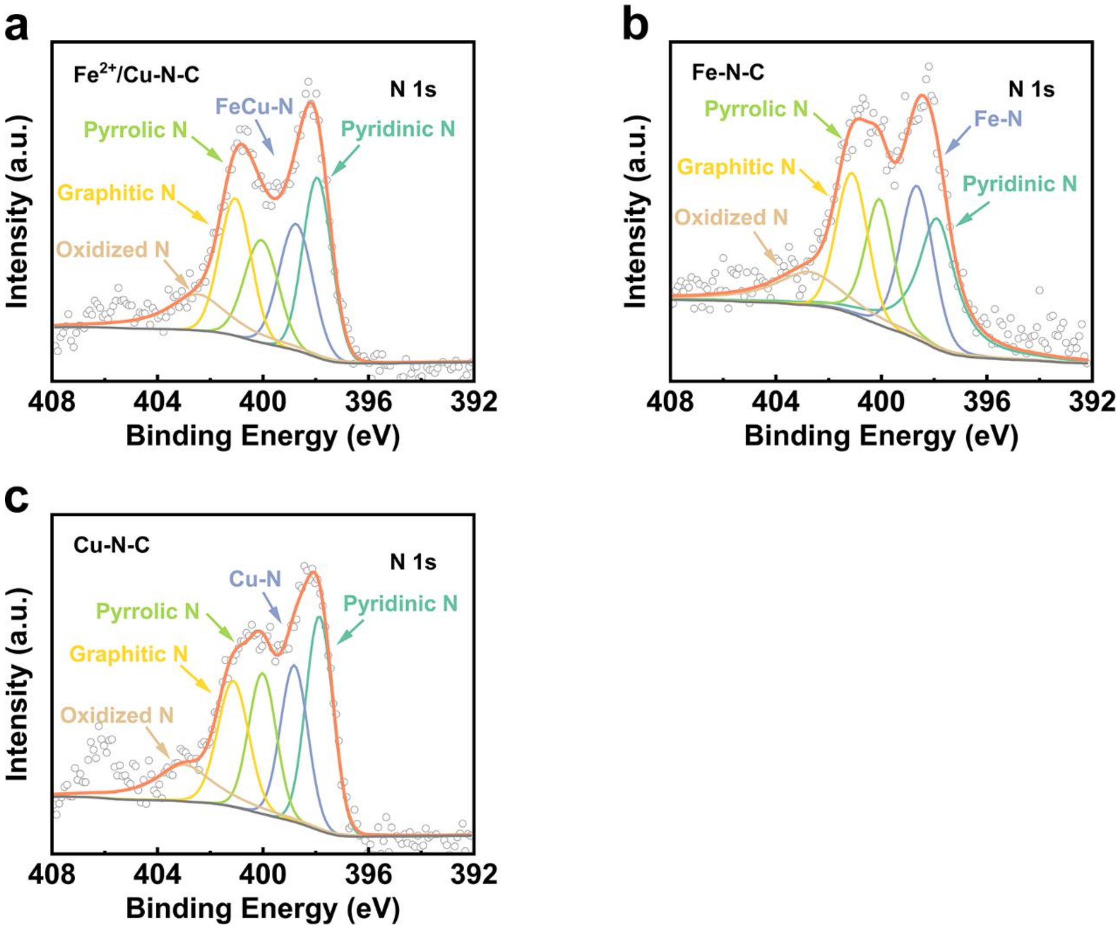

Fig. S6: High-resolution XPS spectra of N 1s for (a) Fe^{2+}/Cu-N-C, (b) Fe-N-C and (c) Cu-N-C.

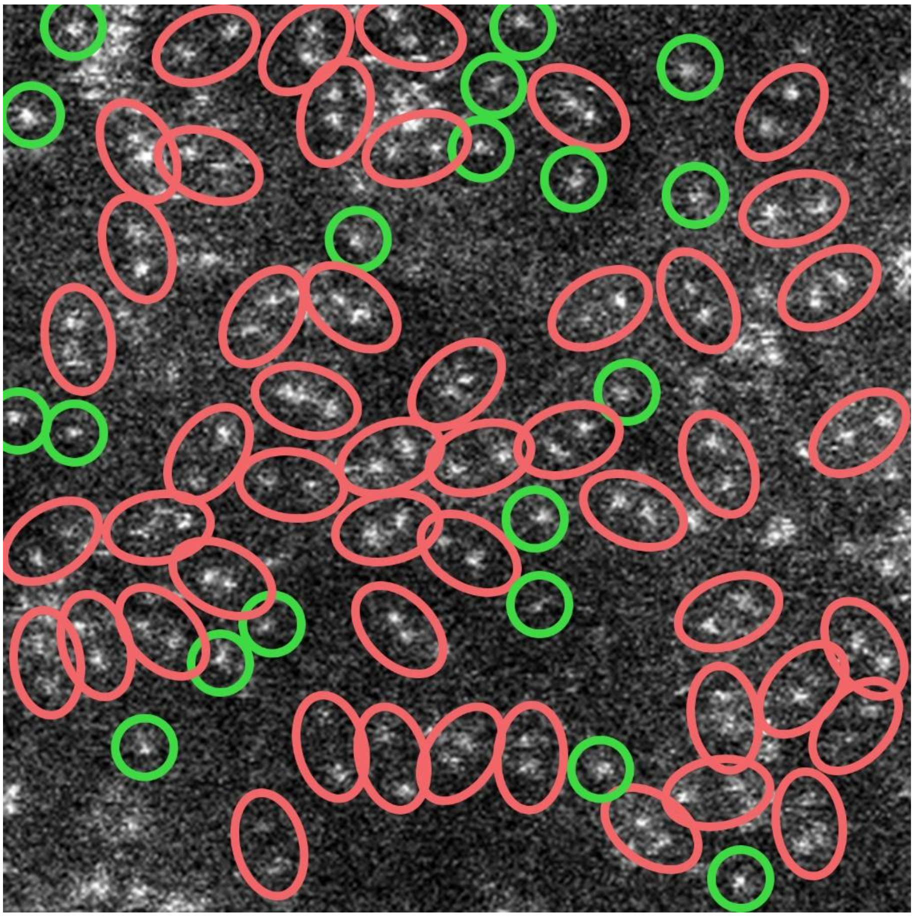

Fig. S7: Magnified atomic-resolution HAADF-STEM image of the red frame area. Fe/Cu atomic pair and individual Fe or Cu atoms are marked with red and green circles, respectively.

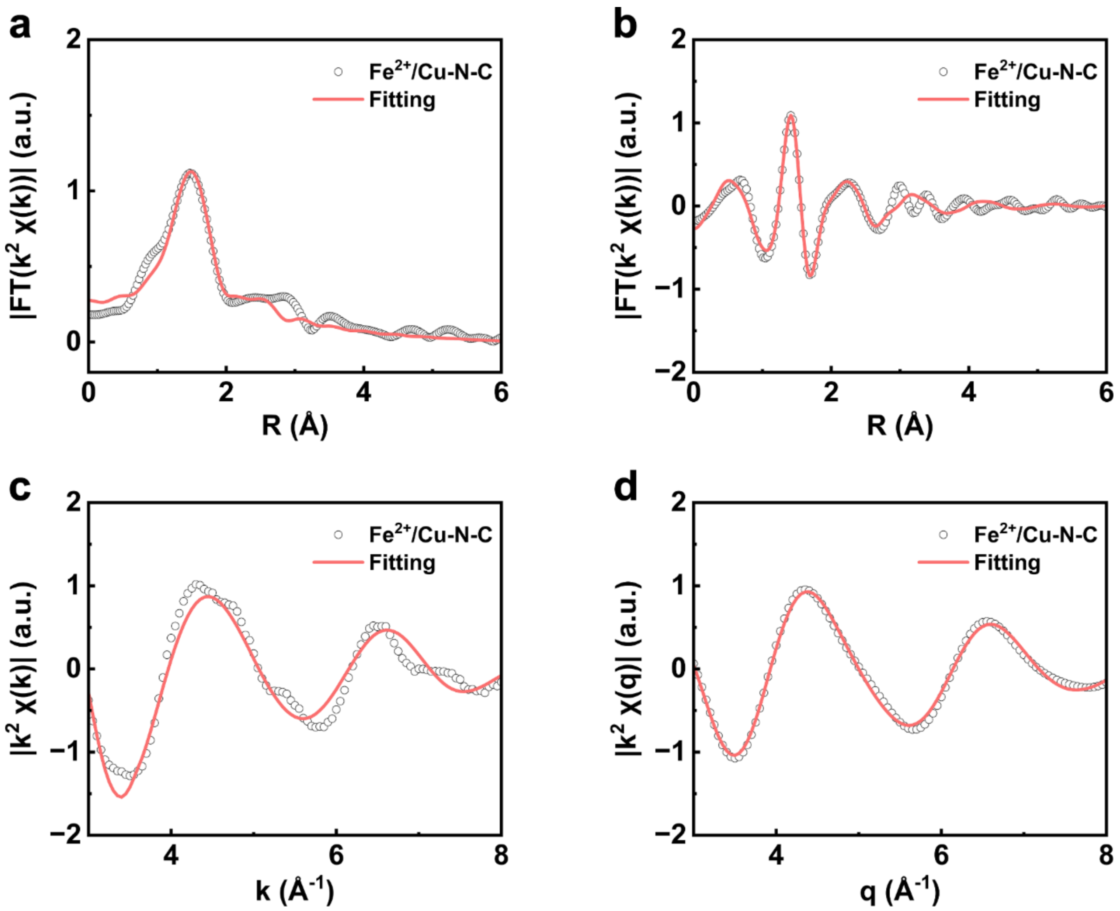

Fig. S8: Fe K-edge EXAFS and curves fit of Fe^{2+}/Cu-N-C under ex situ for (a) FT magnitude, (b) imaginary component of R-space, (c) k-space and (d) q-space. The data are K^2-weighted without phase-correction.

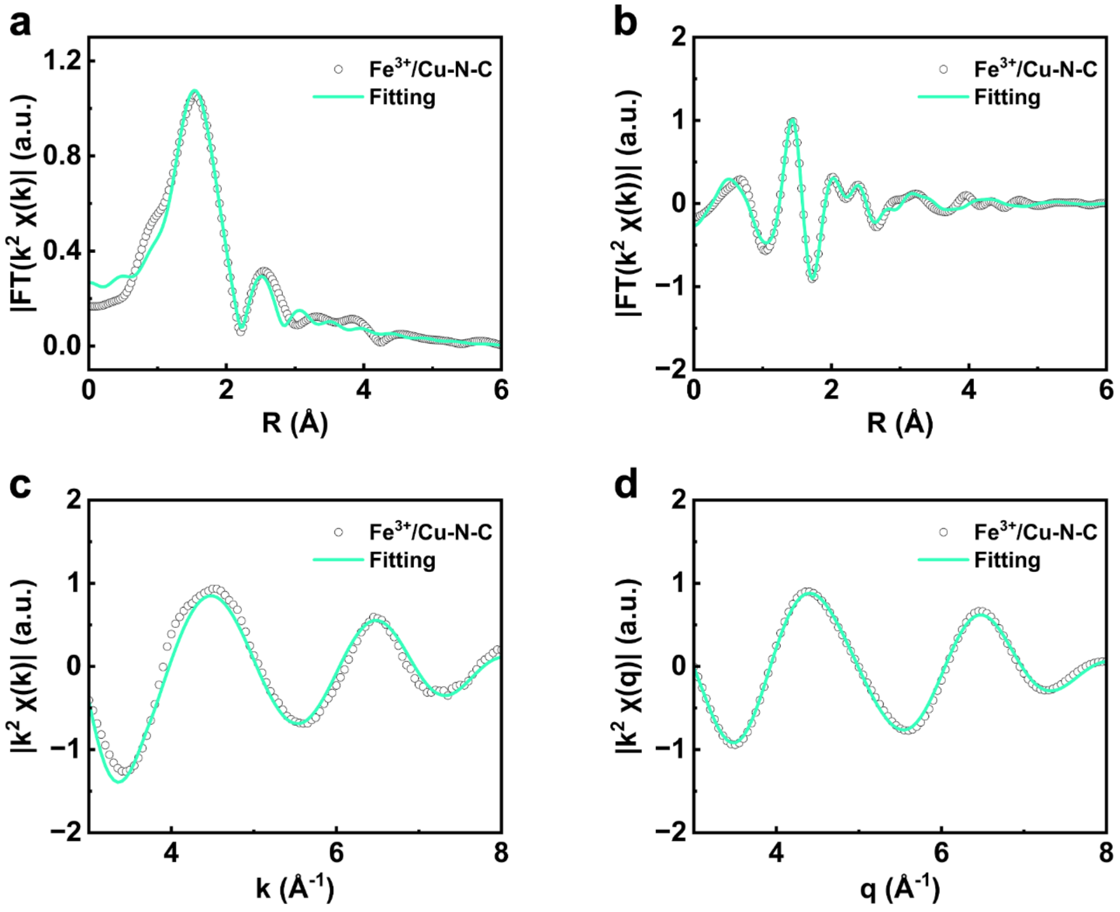

Fig. S9: Fe K-edge EXAFS and curves fit of Fe^{3+}/Cu-N-C under ex situ for (a) FT magnitude, (b) imaginary component of R-space, (c) k-space and (d) q-space. The data are K^2-weighted without phase-correction.

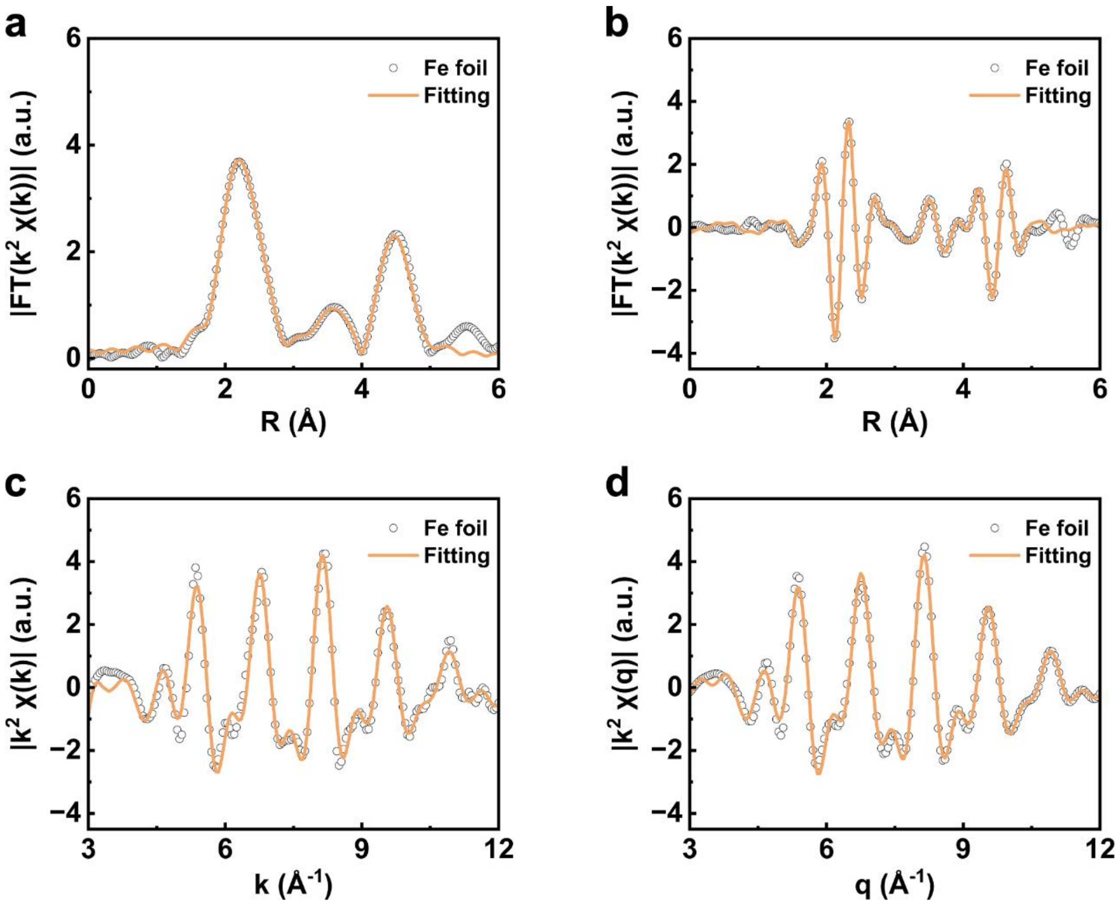

Fig. S10: Fe-K edge EXAFS and curves fit of Fe foil under ex situ for (a) FT magnitude, (b) imaginary component of R-space, (c) k-space and (d) q-space. The data are K^2-weighted without phase-correction.

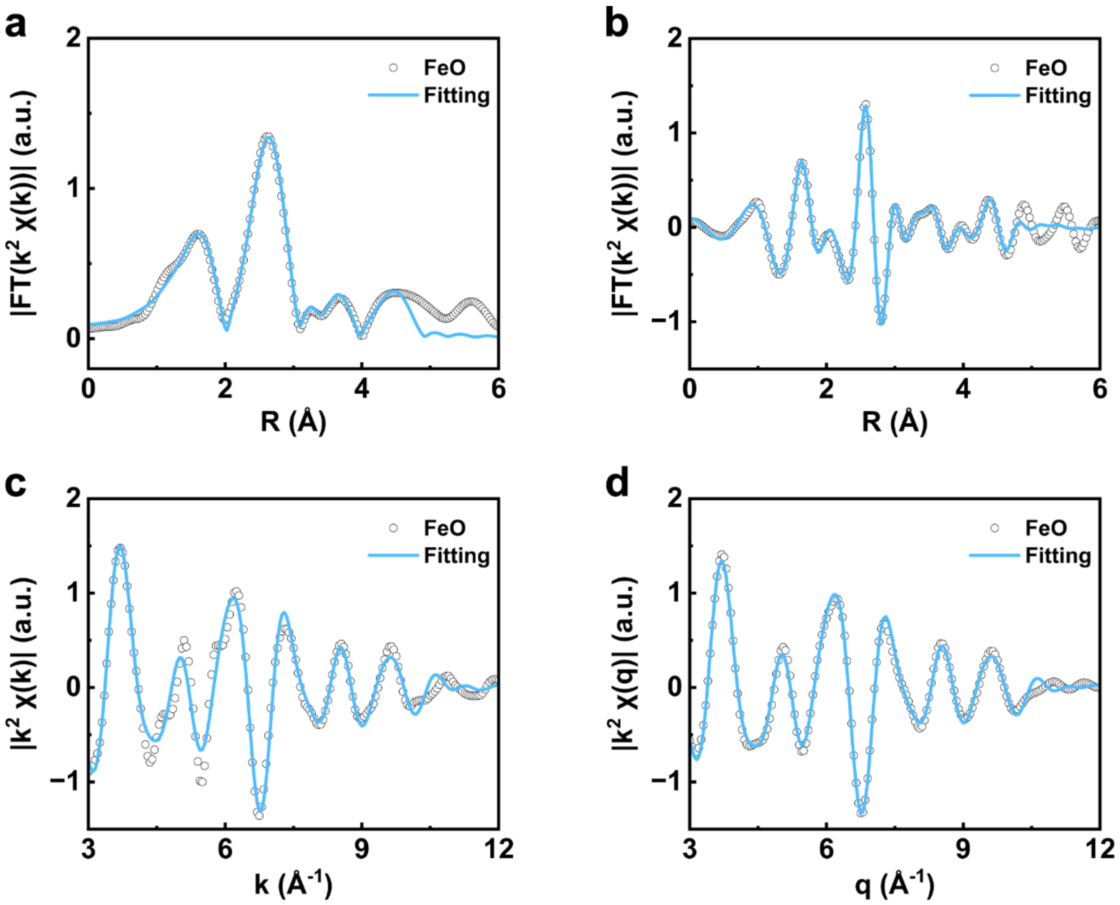

Fig. S11: Fe K-edge EXAFS and curves fit of FeO under ex situ for (a) FT magnitude, (b) imaginary component of R-space, (c) k-space and (d) q-space. The data are K^2-weighted without phase-correction.

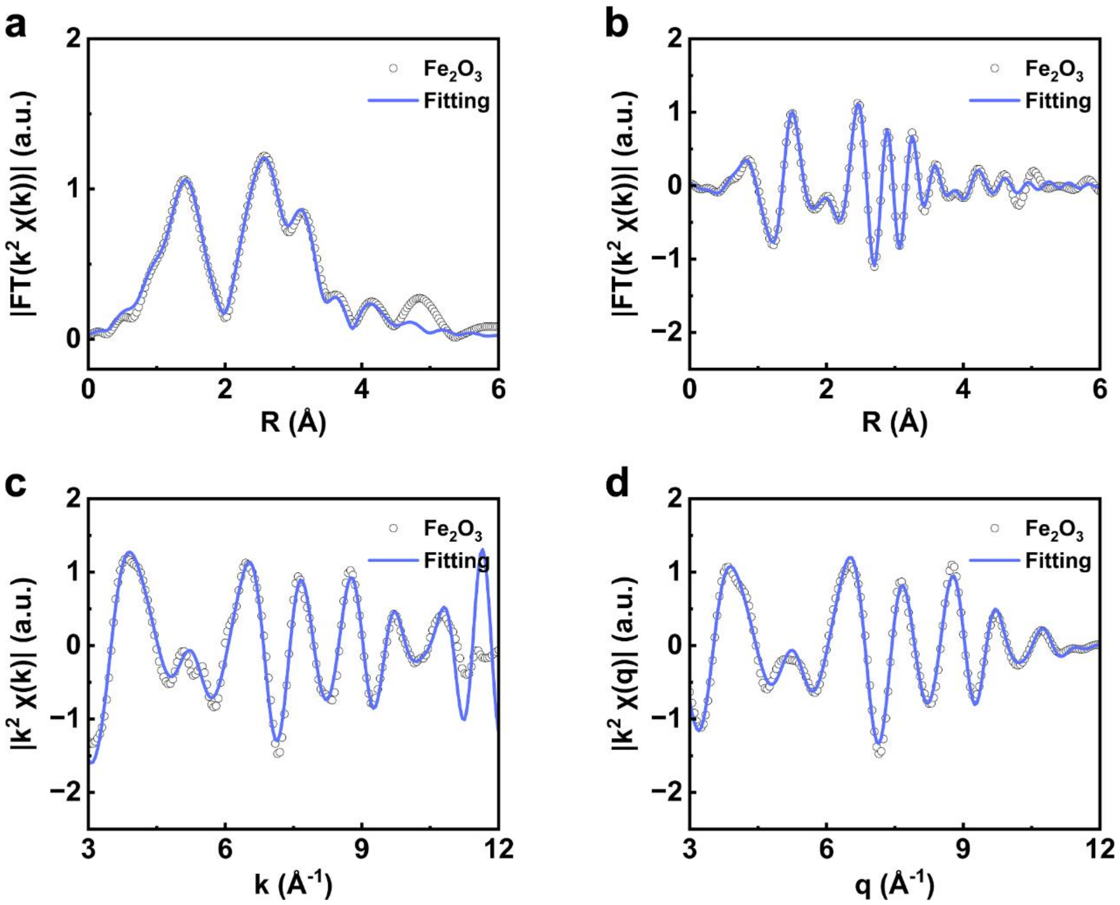

Fig. S12: Fe K-edge EXAFS and curves fit of Fe_2O_3 under ex situ for (a) FT magnitude, (b) imaginary component of R-space, (c) k-space and (d) q-space. The data are K^2-weighted without phase-correction.

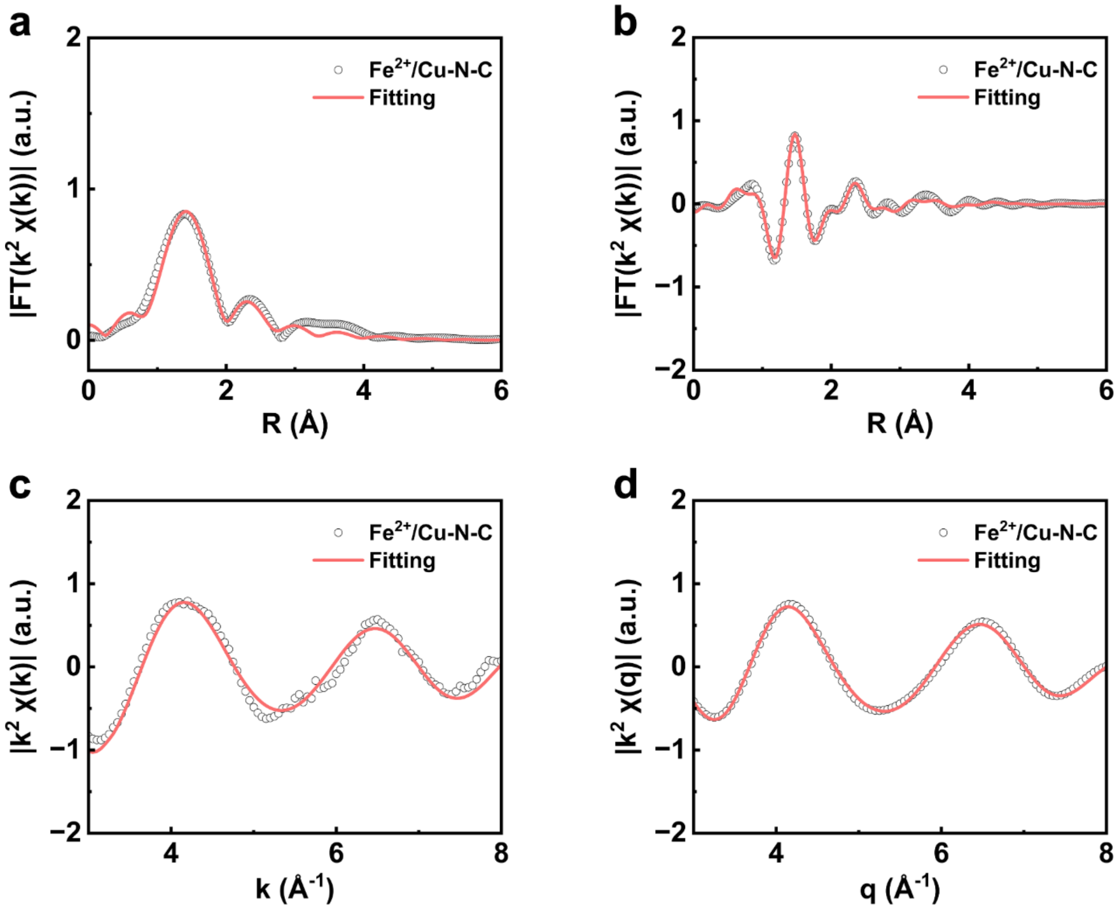

Fig. S13: Cu-K edge EXAFS and curves fit of Fe^{2+}/Cu-N-C under ex situ for (a) FT magnitude, (b) imaginary component of R-space, (c) k-space and (d) q-space. The data are K^2-weighted without phase-correction.

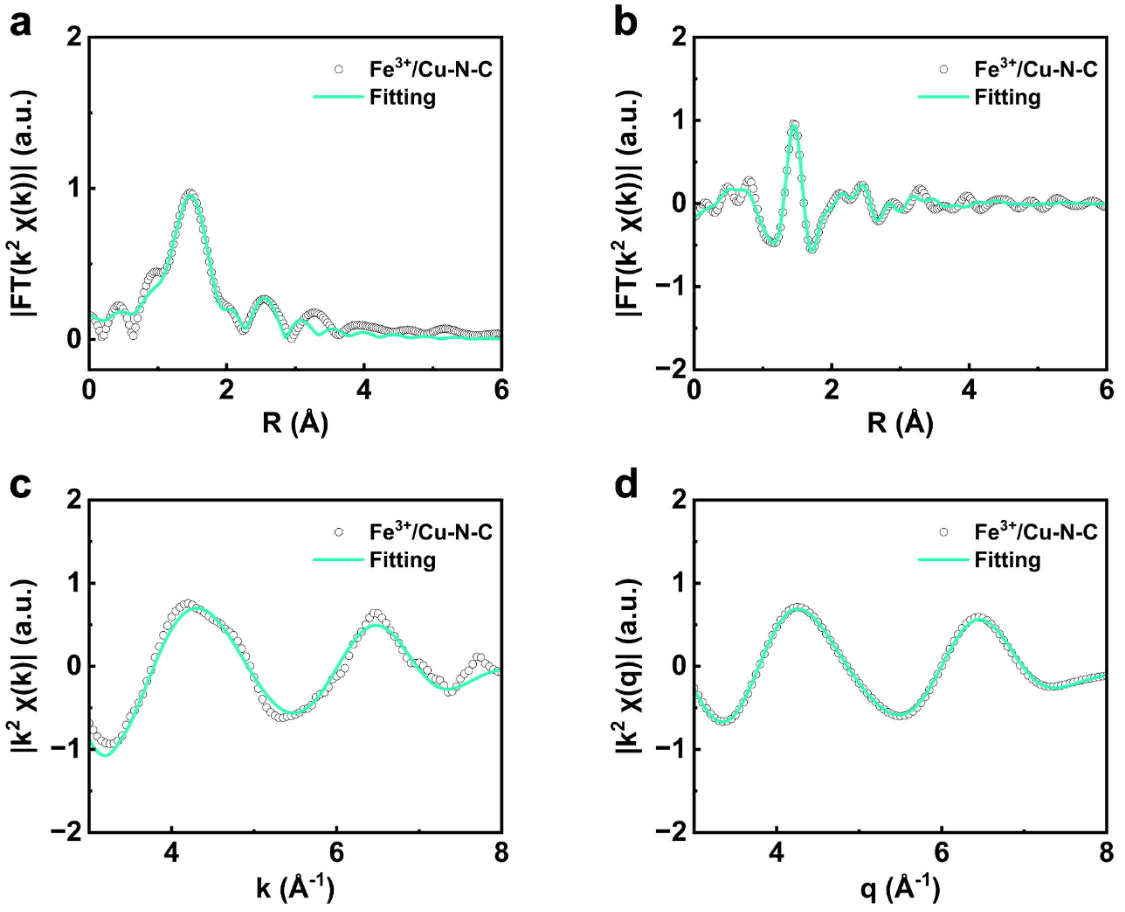

Fig. S14: Cu-K edge EXAFS and curves fit of Fe^{3+}/Cu-N-C under ex situ for (a) FT magnitude, (b) imaginary component of R-space, (c) k-space and (d) q-space. The data are K^2-weighted without phase-correction.

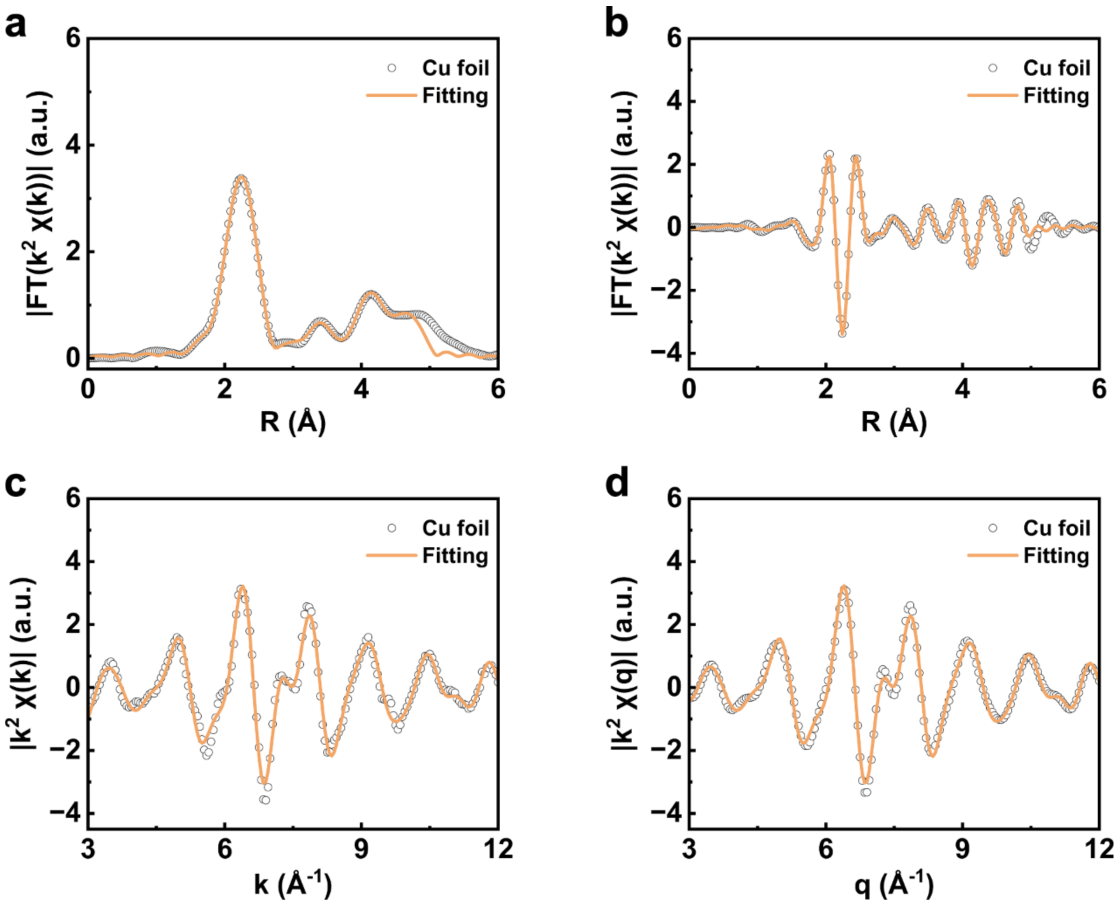

Fig. S15: Cu K-edge EXAFS and curves fit of Cu foil under ex situ for (a) FT magnitude, (b) imaginary component of R-space, (c) k-space and (d) q-space. The data are K^2-weighted without phase-correction.

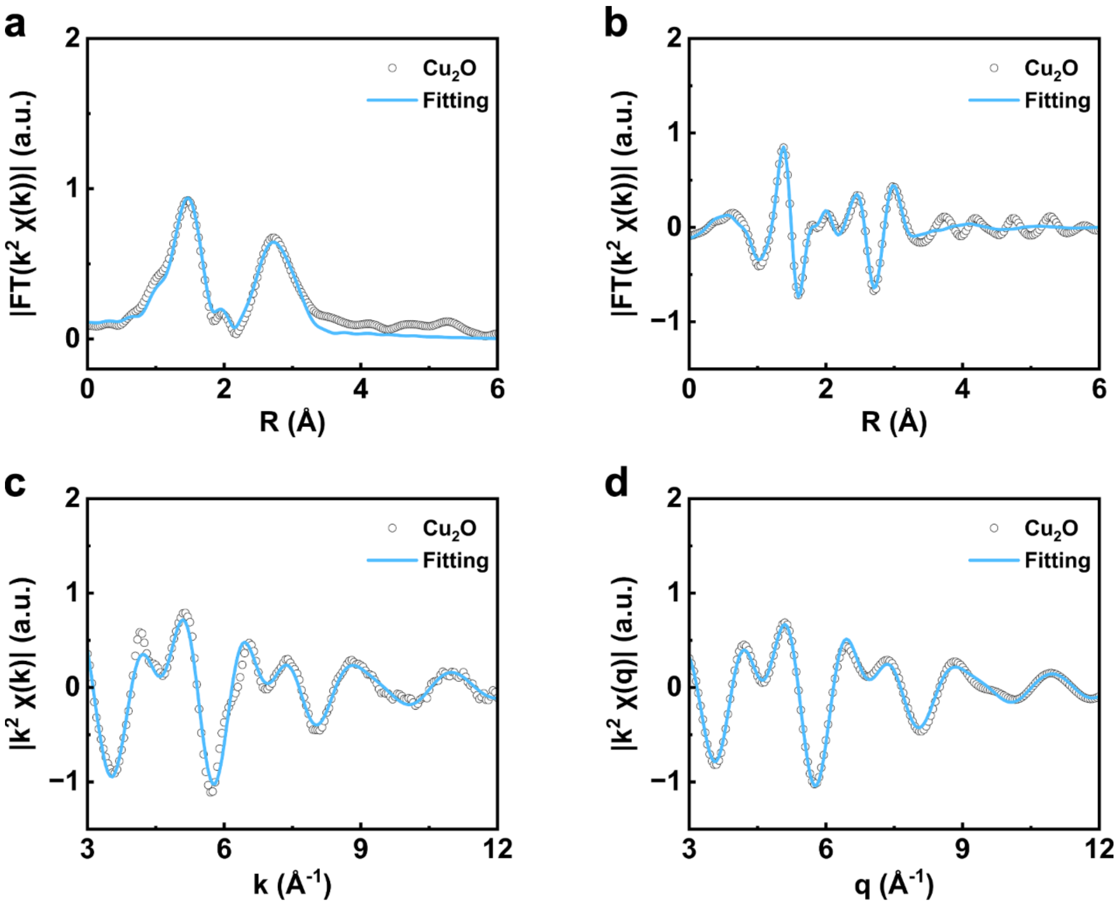

Fig. S16: Cu-K edge EXAFS and curves fit of Cu_2O under ex situ for (a) FT magnitude, (b) imaginary component of R-space, (c) k-space and (d) q-space. The data are K^2-weighted without phase-correction.

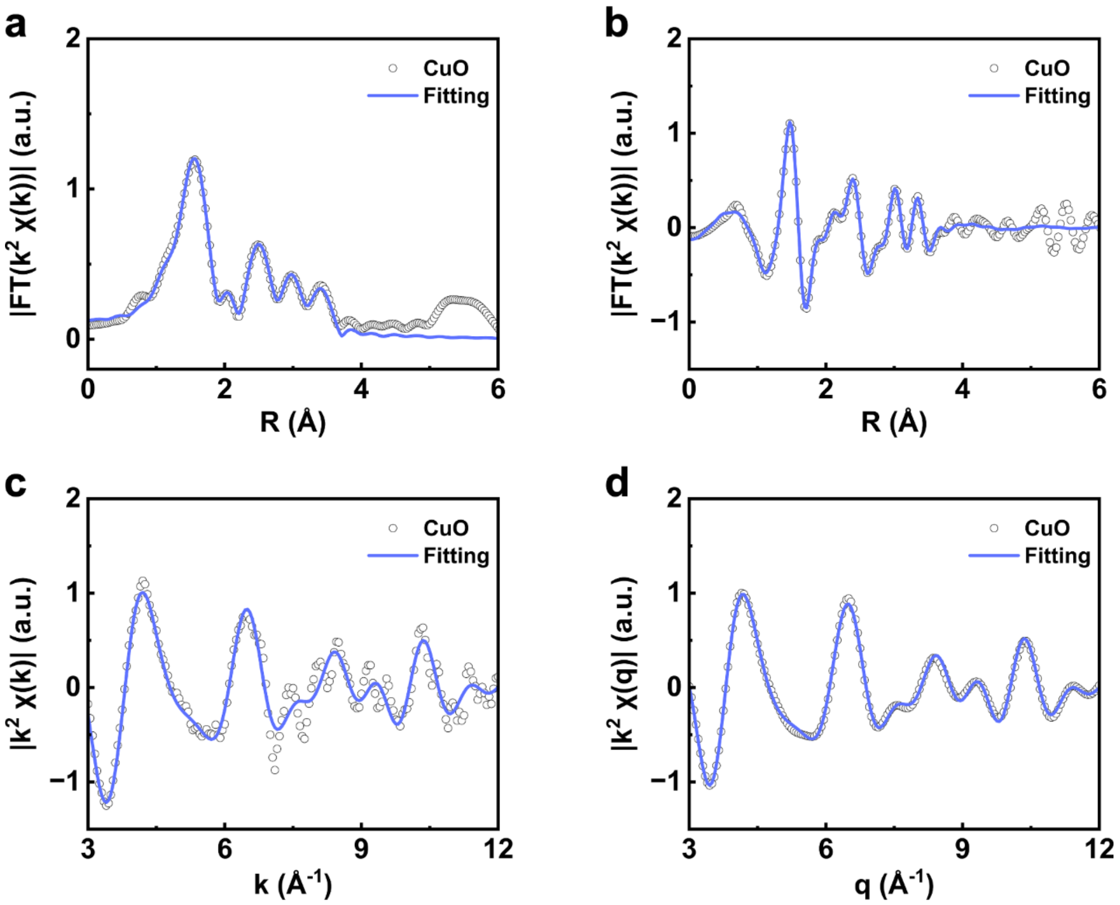

Fig. S17: Cu-K edge EXAFS and curves fit of CuO under ex situ for (a) FT magnitude, (b) imaginary component of R-space, (c) k-space and (d) q-space. The data are K^2-weighted without phase-correction.

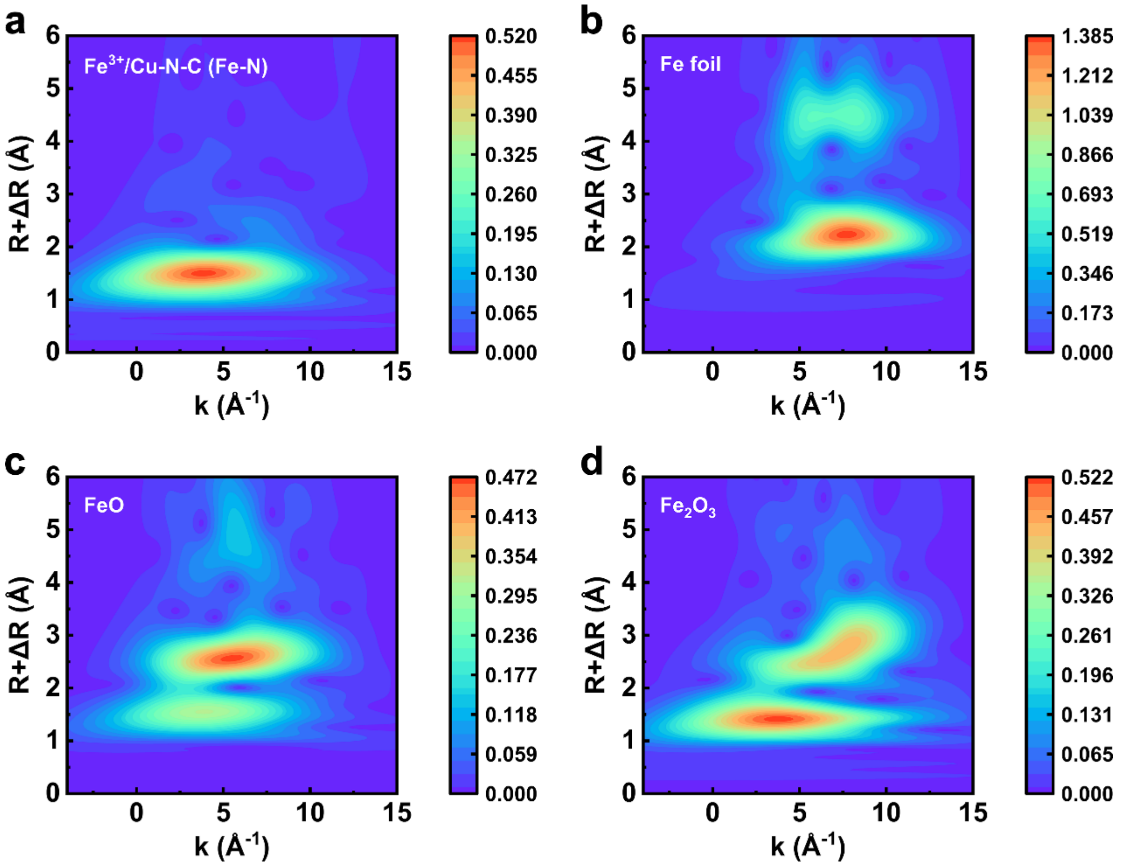

Fig. S18: EXAFS wavelet transforms of Fe K-edge for (a) Fe^{3+}/Cu-N-C, (b) Fe foil, (c) FeO and (d) Fe_2O_3.

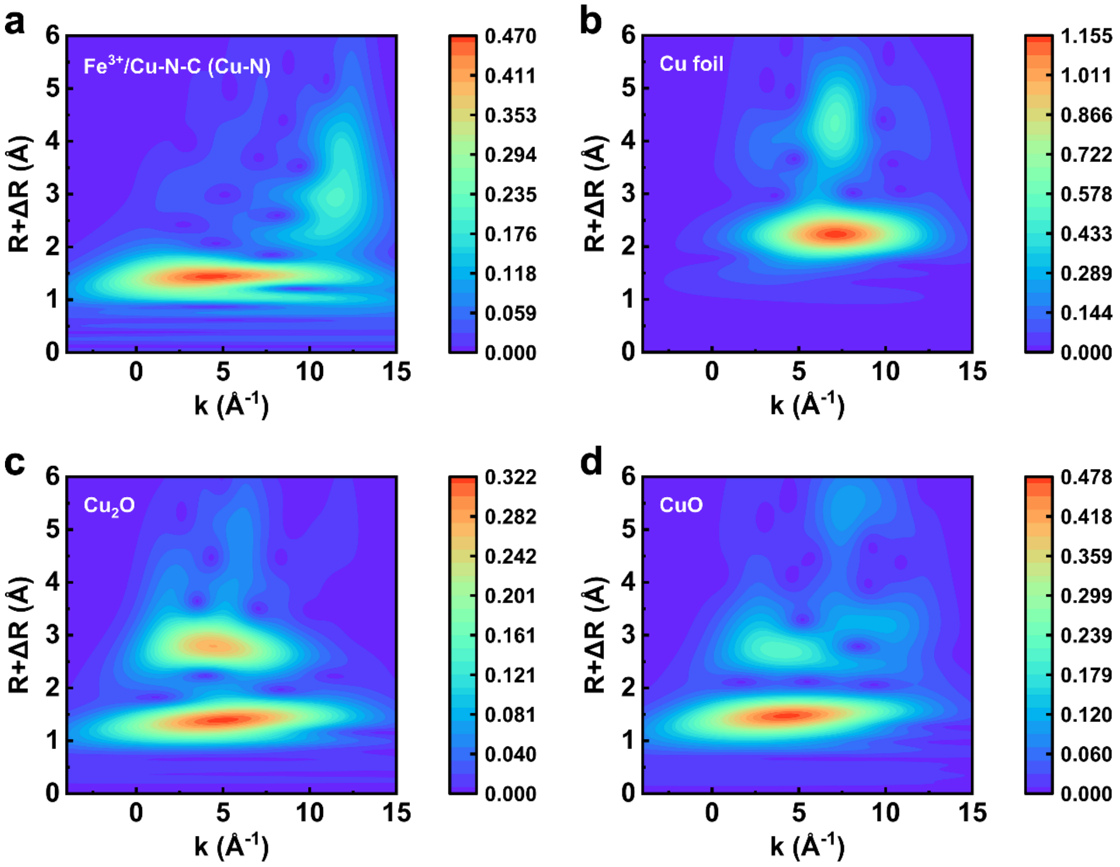

Fig. S19: EXAFS wavelet transforms of Cu K-edge for (a) Fe^{3+}/Cu-N-C, (b) Cu foil, (c) Cu_2O and (d) CuO.

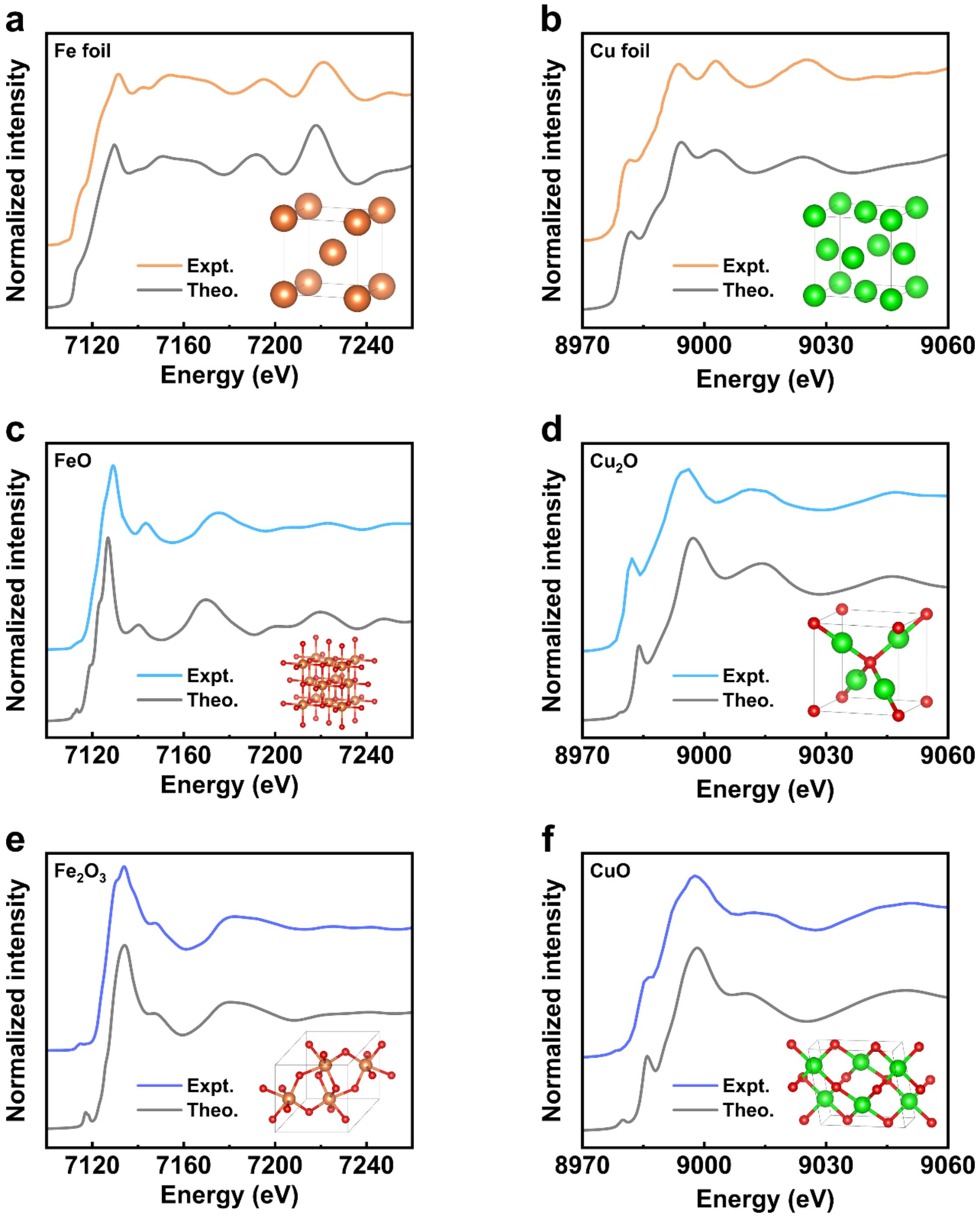

Fig. S20: Comparison between the experimental Fe and Cu K-edge XANES spectra for (a) Fe foil (b) Cu foil (c) FeO (d) Cu_2O (e) Fe_2O_3 and (f) CuO. The corresponding theoretical spectra calculated from different depicted structures were listed in the insets.

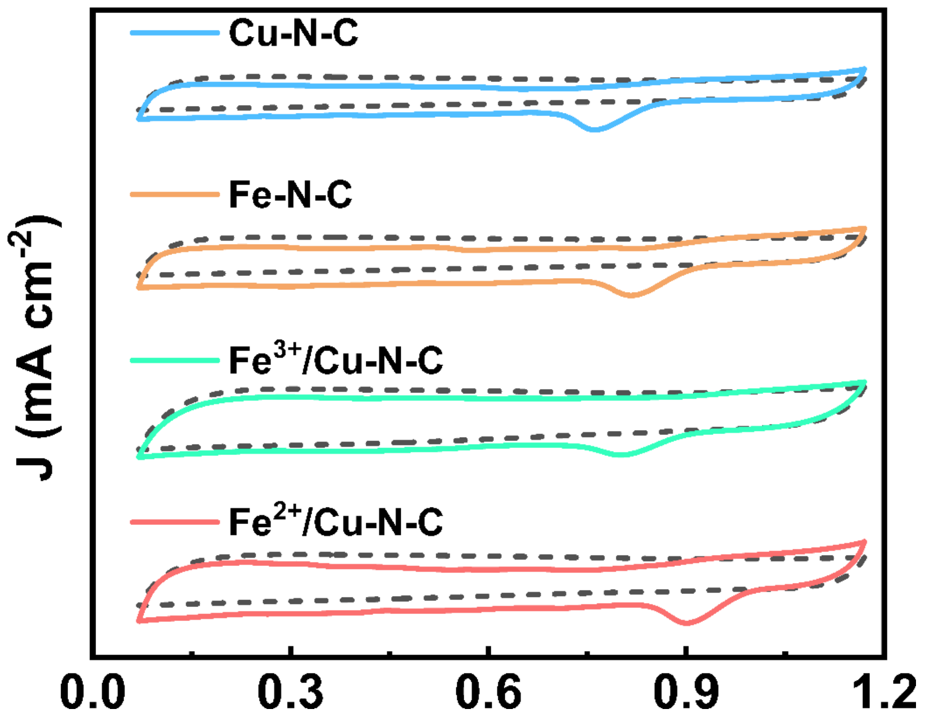

Fig. S21: CV curves of Cu-N-C, Fe-N-C, Fe^{3+}/Cu-N-C and Fe^{2+}/Cu-N-C in 0.1 M KOH at a scan rate of 50 mV s^{-1} saturated with N_2 (dashed curves) or O_2 (solid curves).

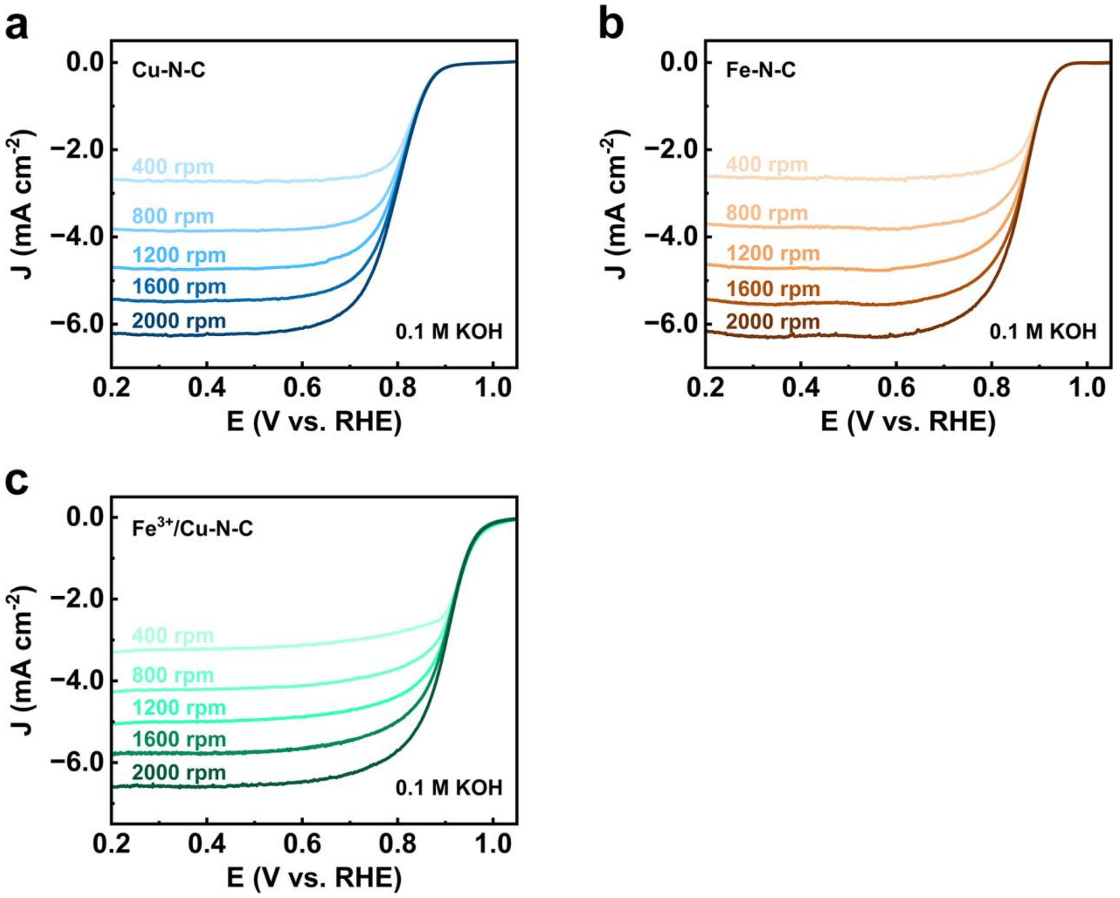

Fig. S22: Fitting the number of transferred electrons： LSV curves at different rotation rates in rpm for (a) Cu-N-C, (b) Fe-N-C and (c) Fe^{3+}/Cu-N-C.

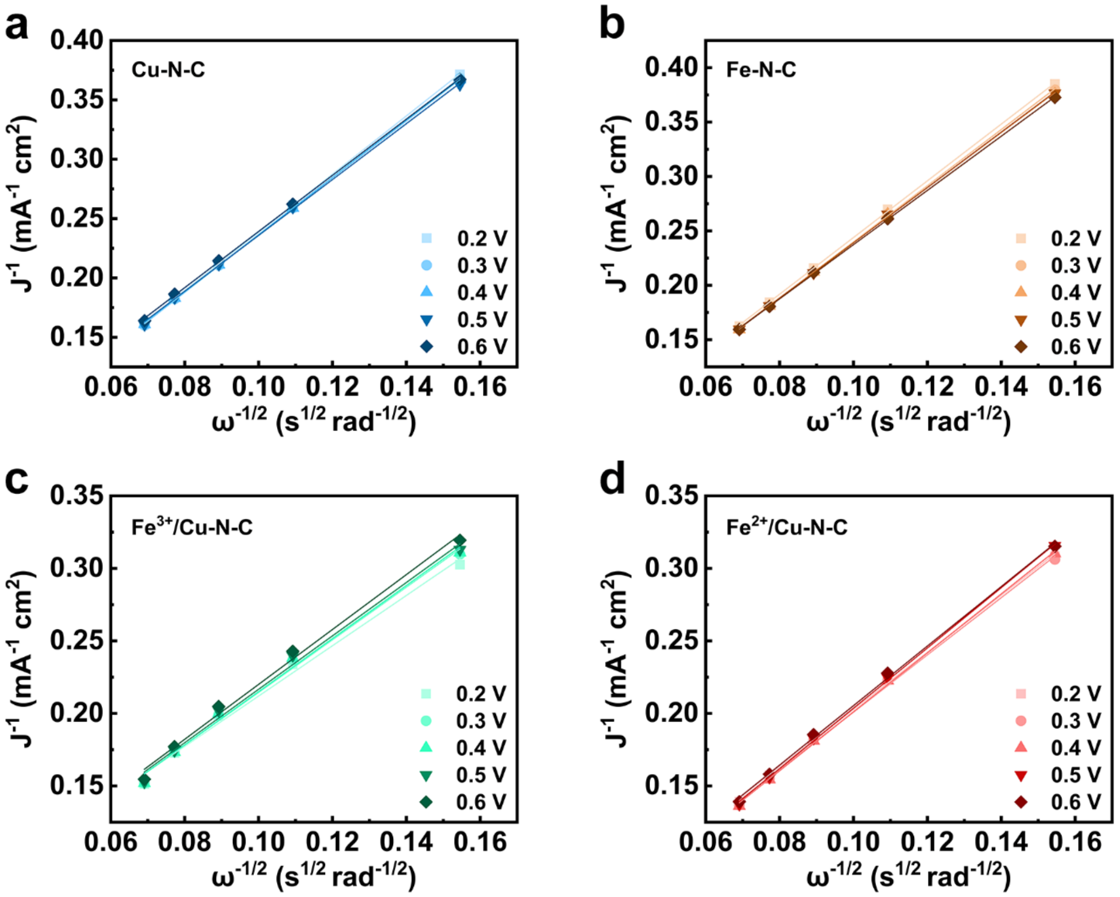

Fig. S23: Koutecky-Levich plots at different applied potentials for (a) Cu-N-C, (b) Fe-N-C, (c) Fe^{3+}/Cu-N-C and (d) Fe^{2+}/Cu-N-C.

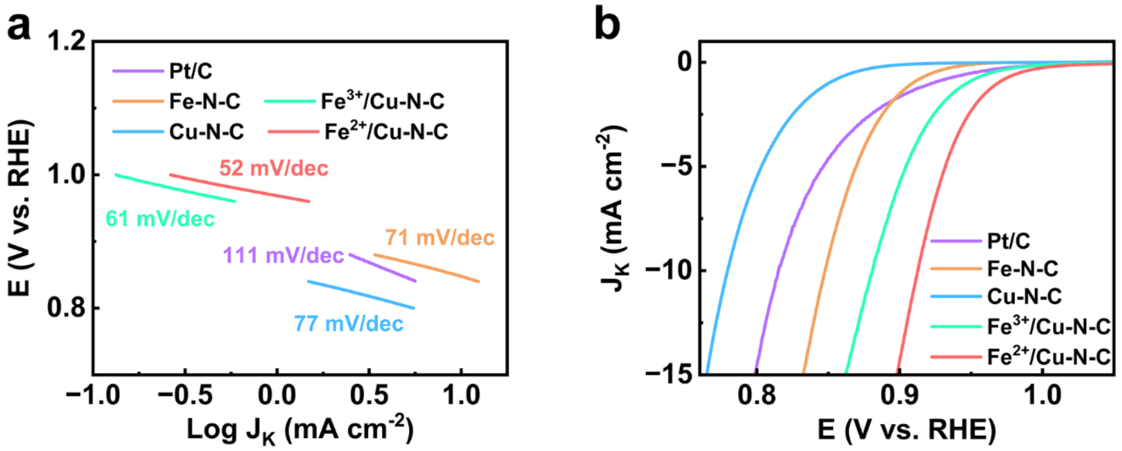

Fig. S24: ORR kinetics study of the as-synthesized catalysts in O_2-saturated 0.1 M KOH solution for (a) the Tafel slopes and (b) kinetic current densities.

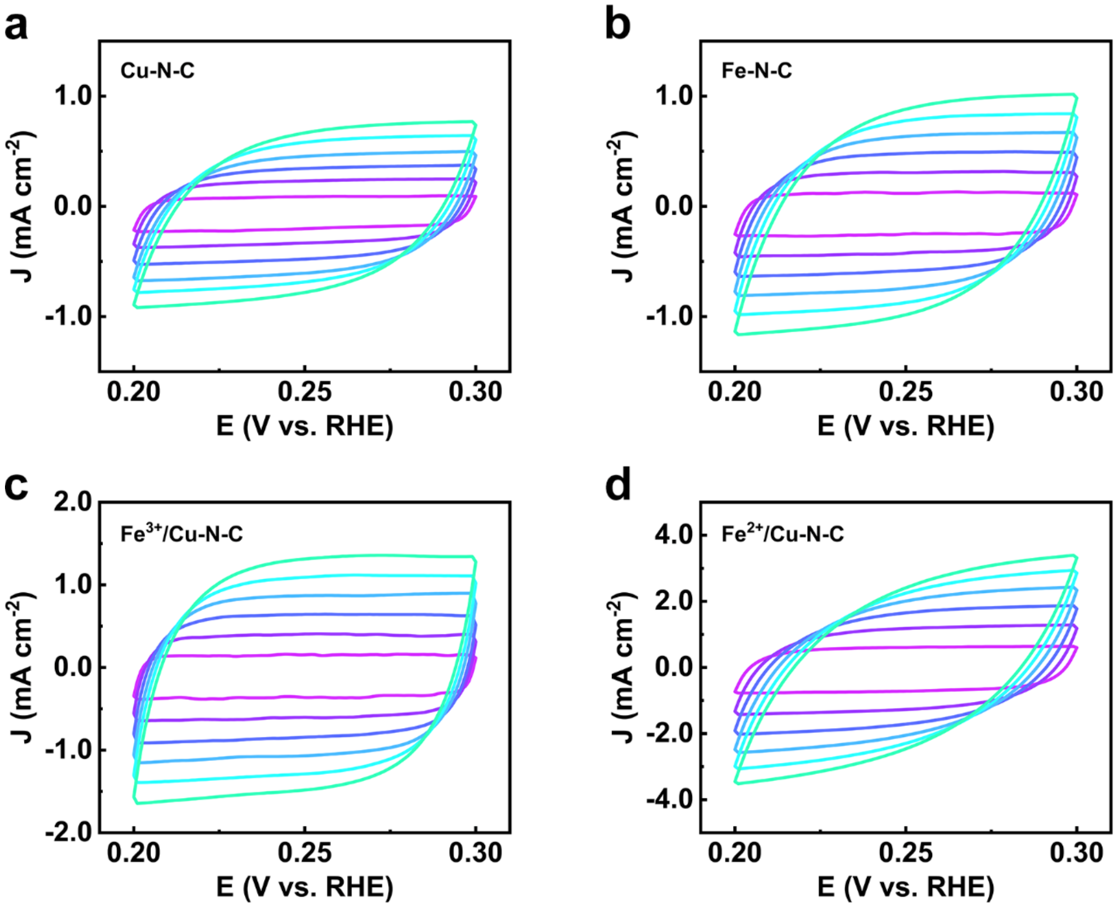

Fig. S25: CV curves in the double layer region at scan rates of 5, 10, 15, 20, 25 and 30 mV s^{-1} in 0.1 M KOH for (a) Cu-N-C, (b) Fe-N-C, (c) Fe^{3+}/Cu-N-C and (d) Fe^{2+}/Cu-N-C.

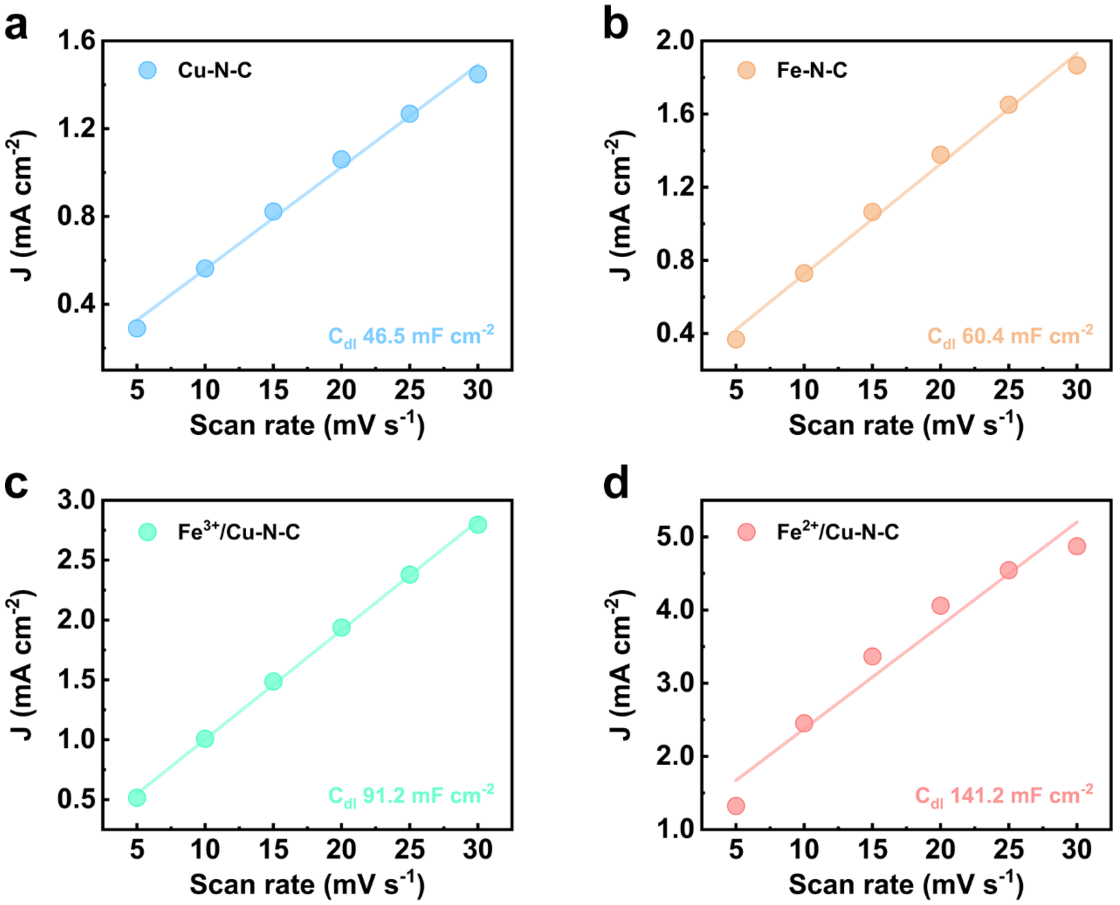

Fig. S26: The differences in the current densities plotted against the scan rates. The slope of the fitting line is equal to the geometric double layer capacitance (C_{dl}) for (a) Cu-N-C, (b) Fe-N-C, (c) Fe^{3+}/Cu-N-C and (d) Fe^{2+}/Cu-N-C.

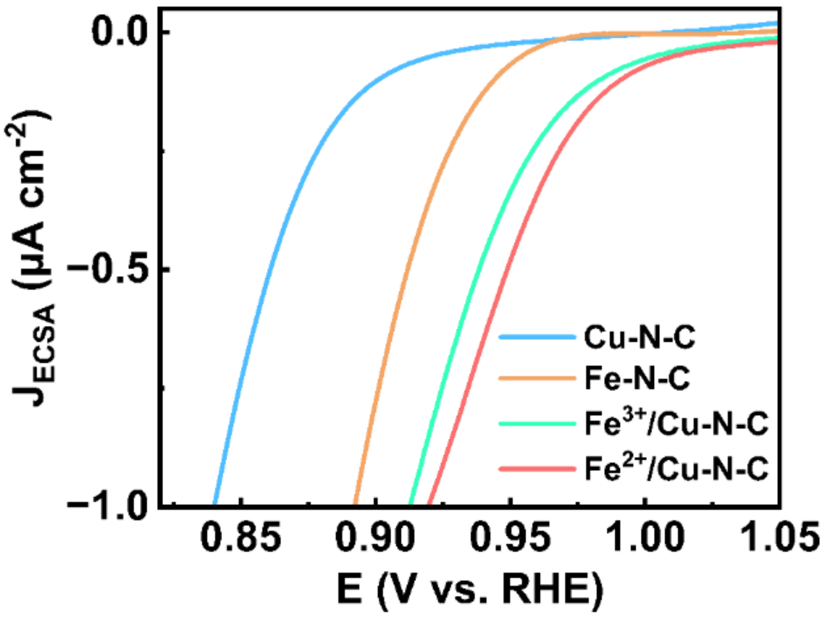

Fig. S27. ECSA-normalized LSV curves of Cu-N-C, Fe-N-C, Fe^{3+}/Cu-N-C and Fe^{2+}/Cu-N-C in 0.1 M KOH.

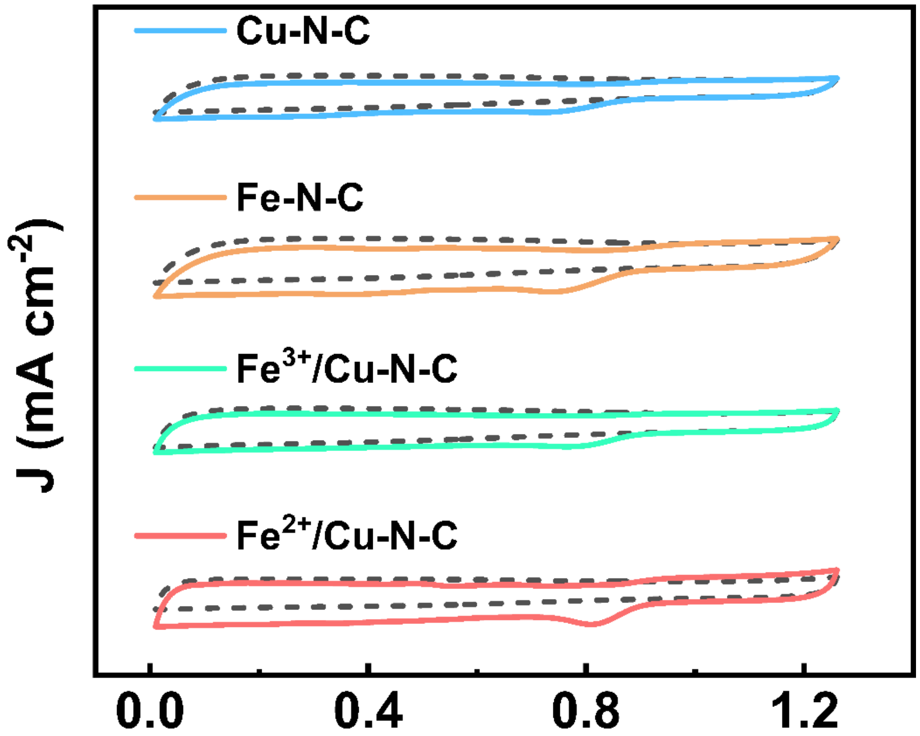

Fig. S28: CV curves of Cu-N-C, Fe-N-C, Fe^{3+}/Cu-N-C and Fe^{2+}/Cu-N-C in 0.1 M $HClO_4$ at a scan rate of 50 mV s^{-1} saturated with N_2 (dashed curves) or O_2 (solid curves).

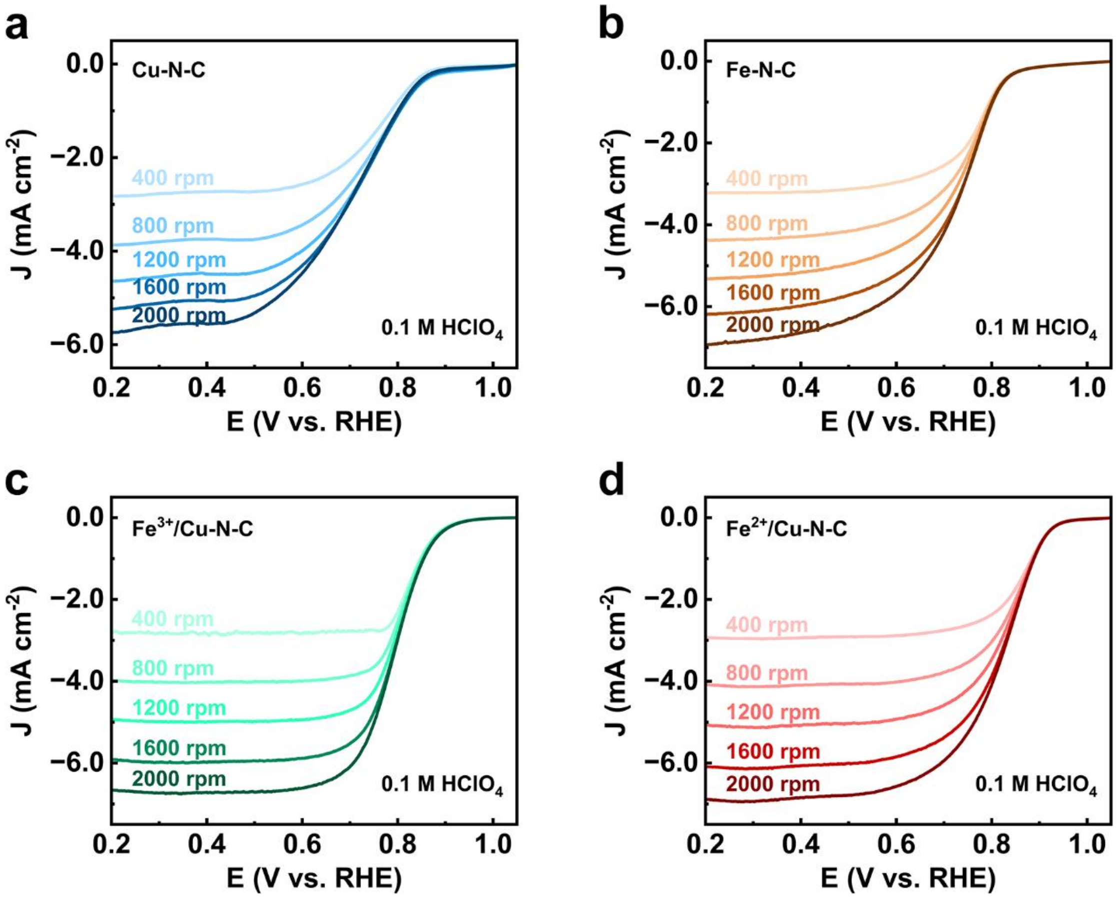

Fig. S29: LSV curves at different rotation rates in rpm for (a) Cu-N-C, (b) Fe-N-C, (c) Fe^{3+}/Cu-N-C and (d) Fe^{2+}/Cu-N-C.

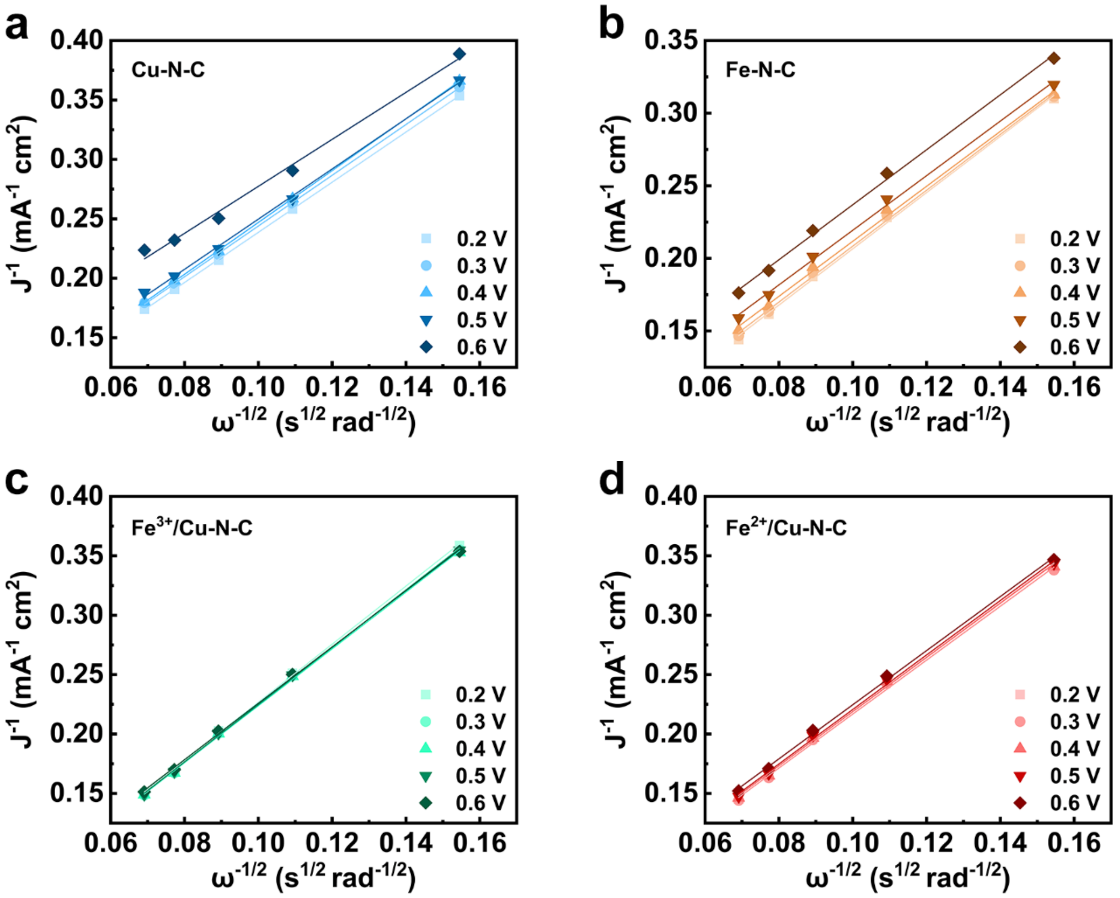

Fig. S30: Koutecky-Levich plots at different applied potentials for (a) Cu-N-C, (b) Fe-N-C, (c) Fe^{3+}/Cu-N-C and (d) Fe^{2+}/Cu-N-C.

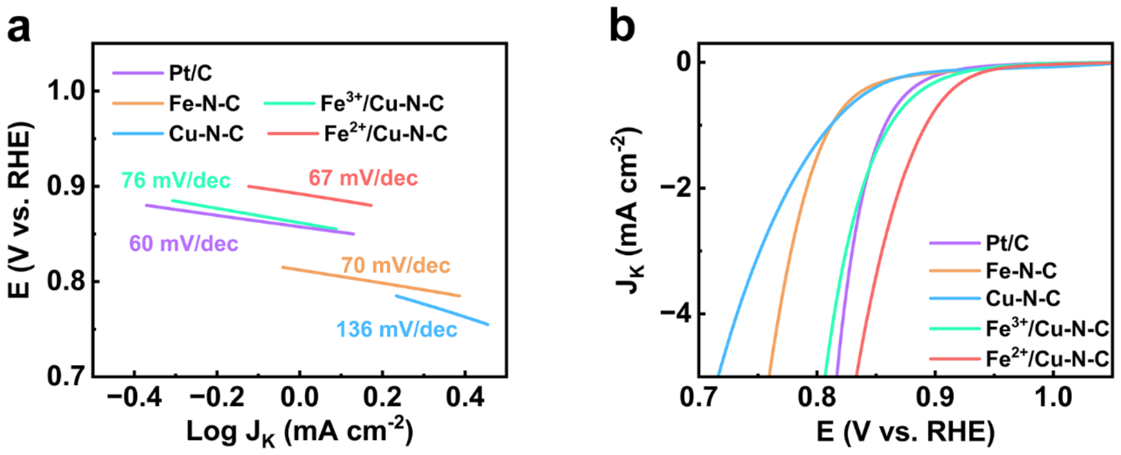

Fig. S31: ORR kinetics study of the as-synthesized catalysts in O_2-saturated 0.1 M $HClO_4$ solution for (a) the Tafel slopes and (b) kinetic current densities.

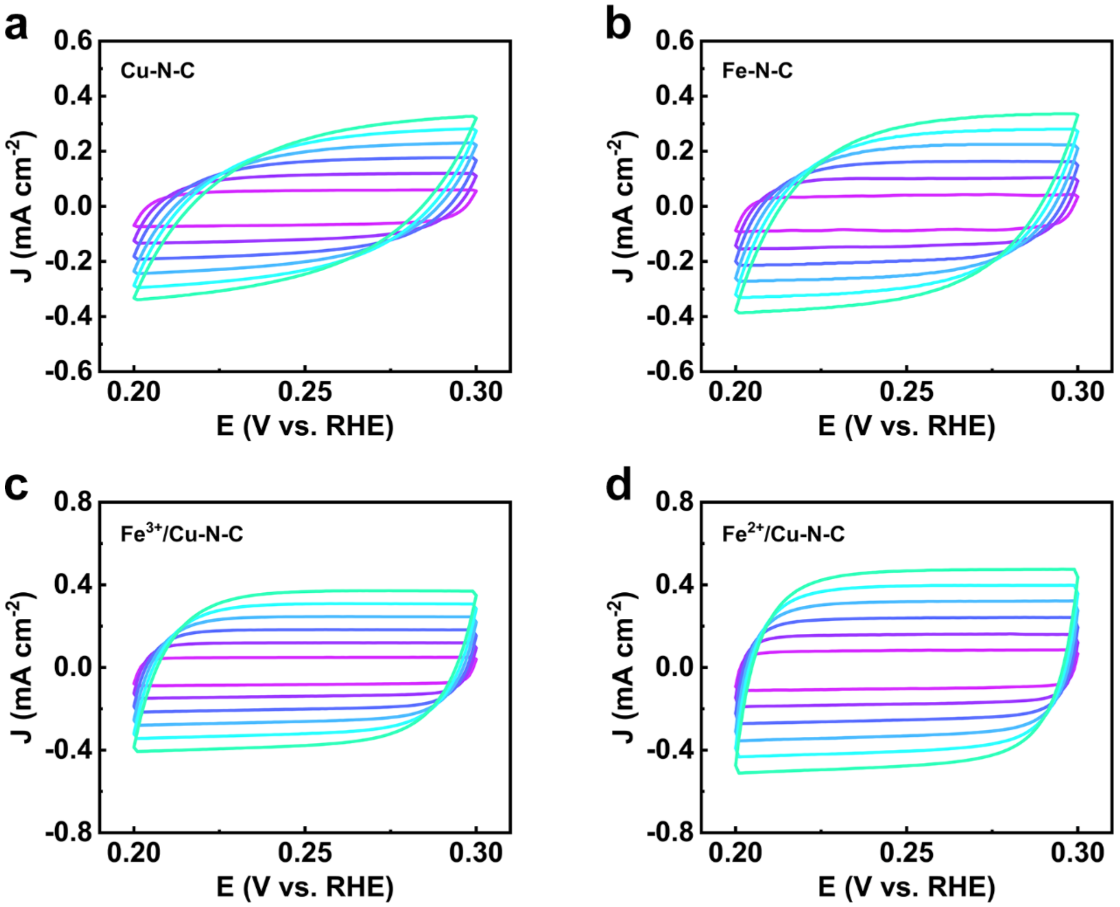

Fig. S32: CV curves in the double layer region at scan rates of 5, 10, 15, 20, 25 and 30 mV s^{-1} in 0.1 M $HClO_4$ for (a) Cu-N-C, (b) Fe-N-C, (c) Fe^{3+}/Cu-N-C and (d) Fe^{2+}/Cu-N-C.

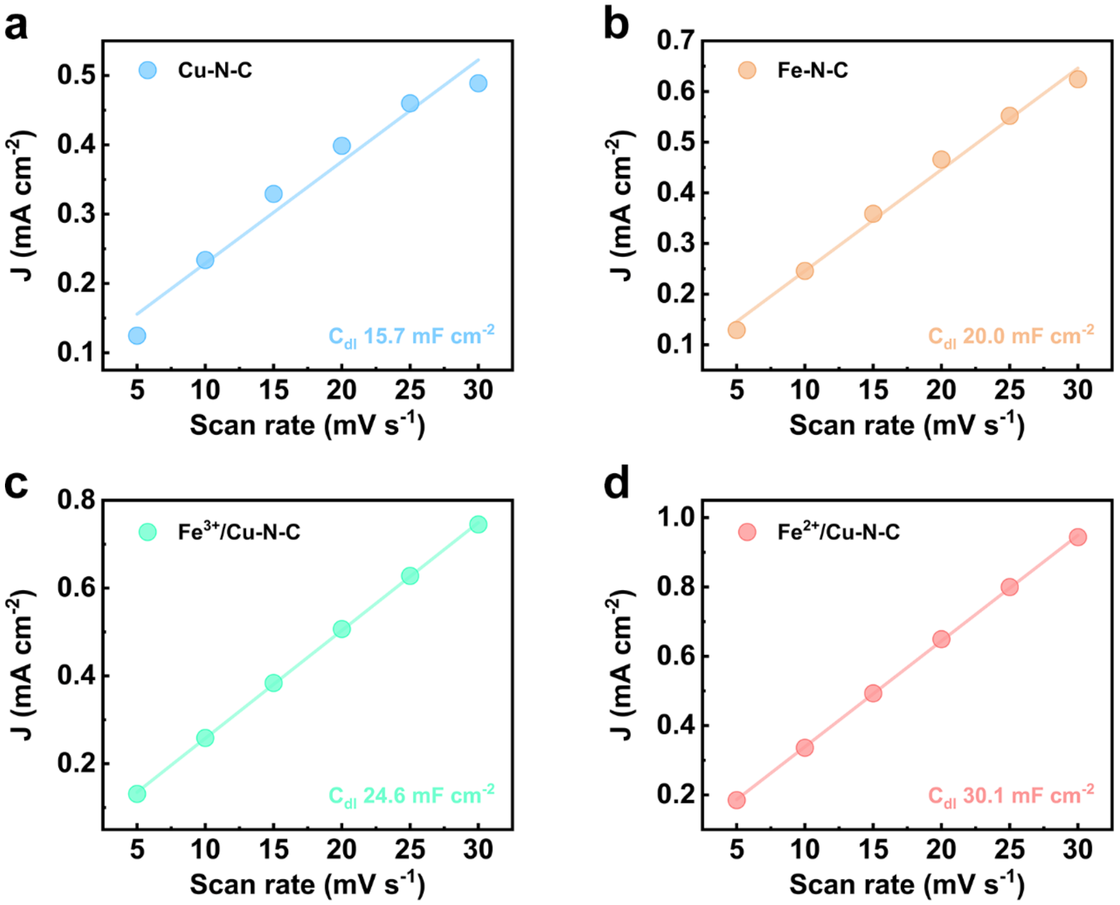

Fig. S33: The differences in the current densities plotted against the scan rates. The slope of fitting line is equal to the geometric double layer capacitance (C_{dl}) for (a) Cu-N-C, (b) Fe-N-C, (c) Fe^{3+}/Cu-N-C and (d) Fe^{2+}/Cu-N-C.

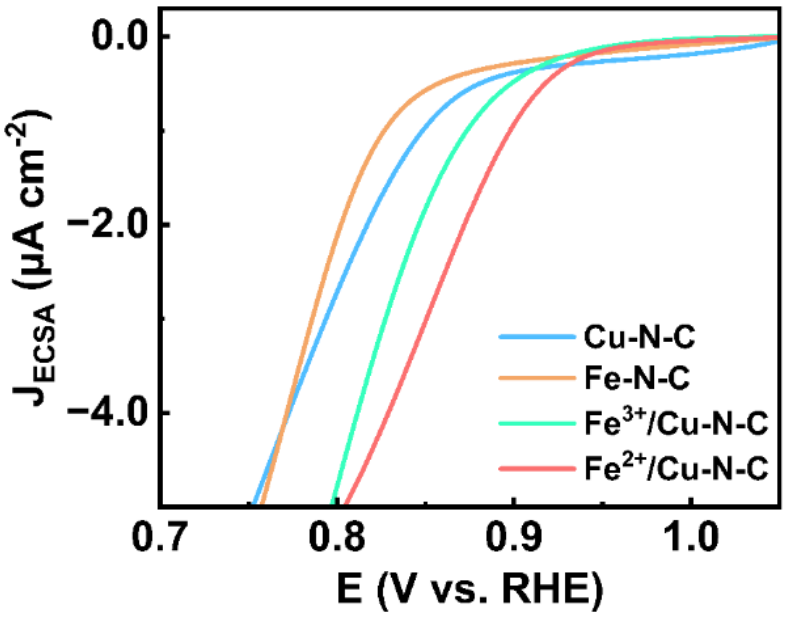

Fig. S34. ECSA-normalized LSV curves of Cu-N-C, Fe-N-C, Fe^{3+}/Cu-N-C and Fe^{2+}/Cu-N-C in 0.1 M $HClO_4$.

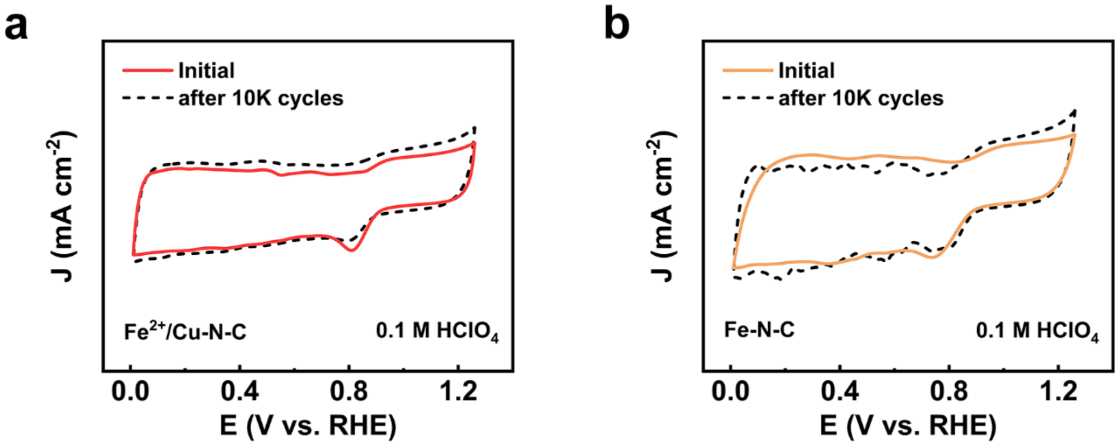

Fig. S35: CV curves before and after 10000 potential cycles at the scan rate of 50 mV s^{-1} in O_2-saturated 0.1 M $HClO_4$ solution for (a) Fe^{2+}/Cu-N-C and (b) Fe-N-C.

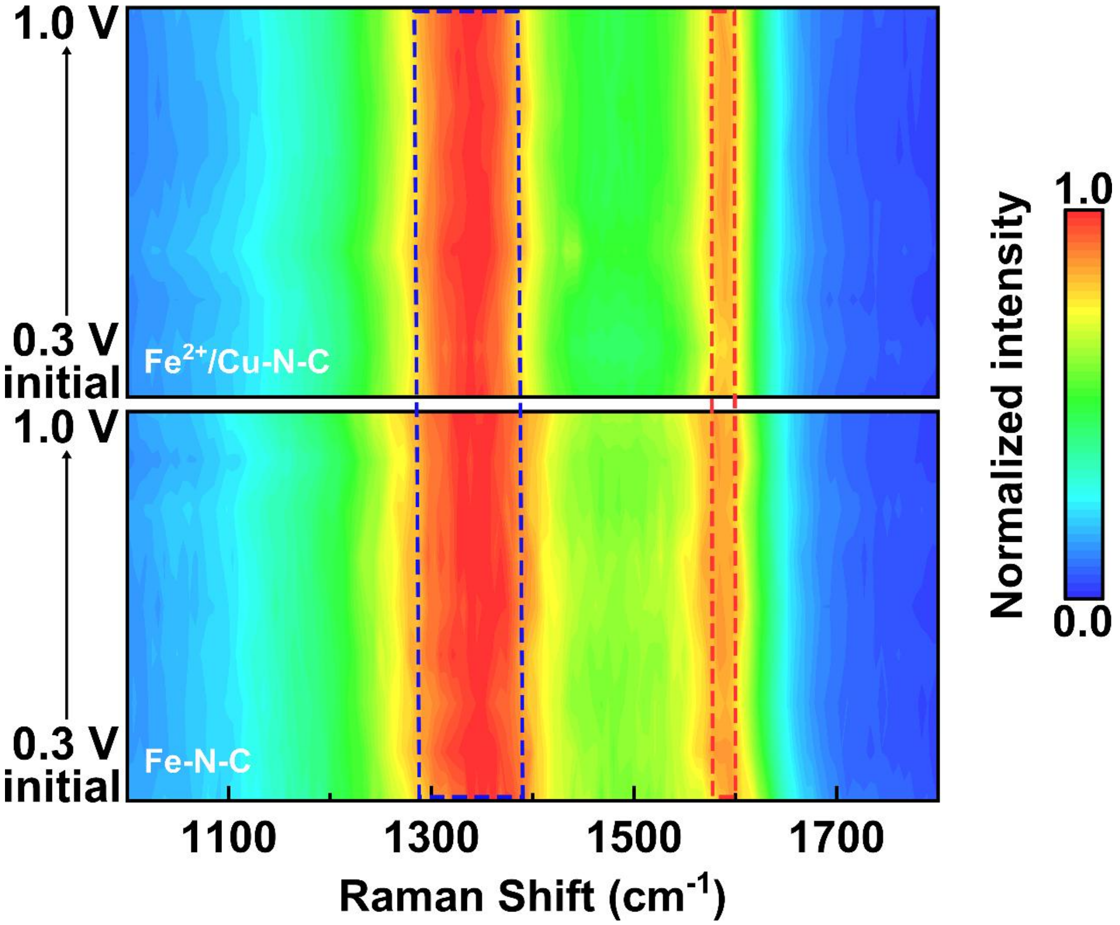

Fig. S36: In situ Raman test of Fe^{2+}/Cu-N-C and Fe-N-C under different applied potentials in O_2-saturated 0.1 M $HClO_4$ solution.

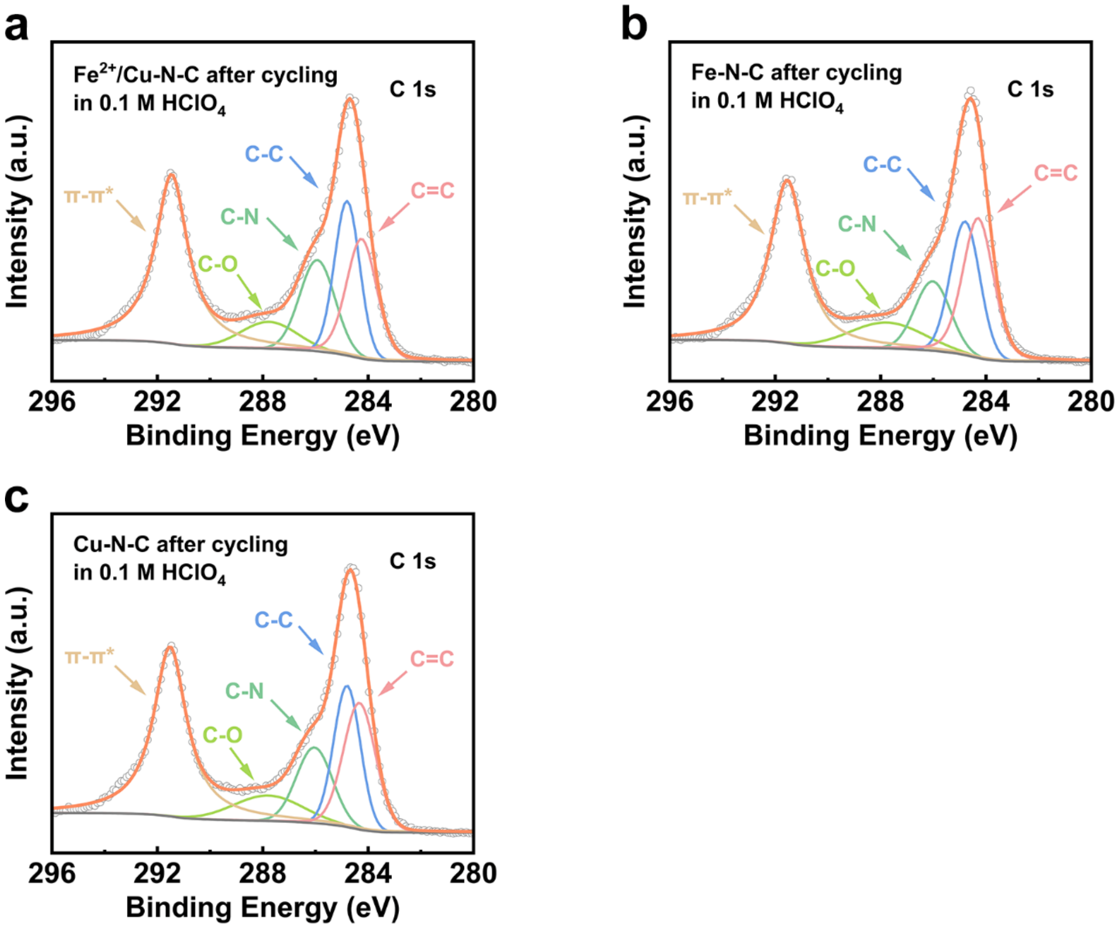

Fig. S37: High-resolution XPS spectra of C 1s after 10000 potential cycles for (a) Fe^{2+}/Cu-N-C, (b) Fe-N-C and (c) Cu-N-C.

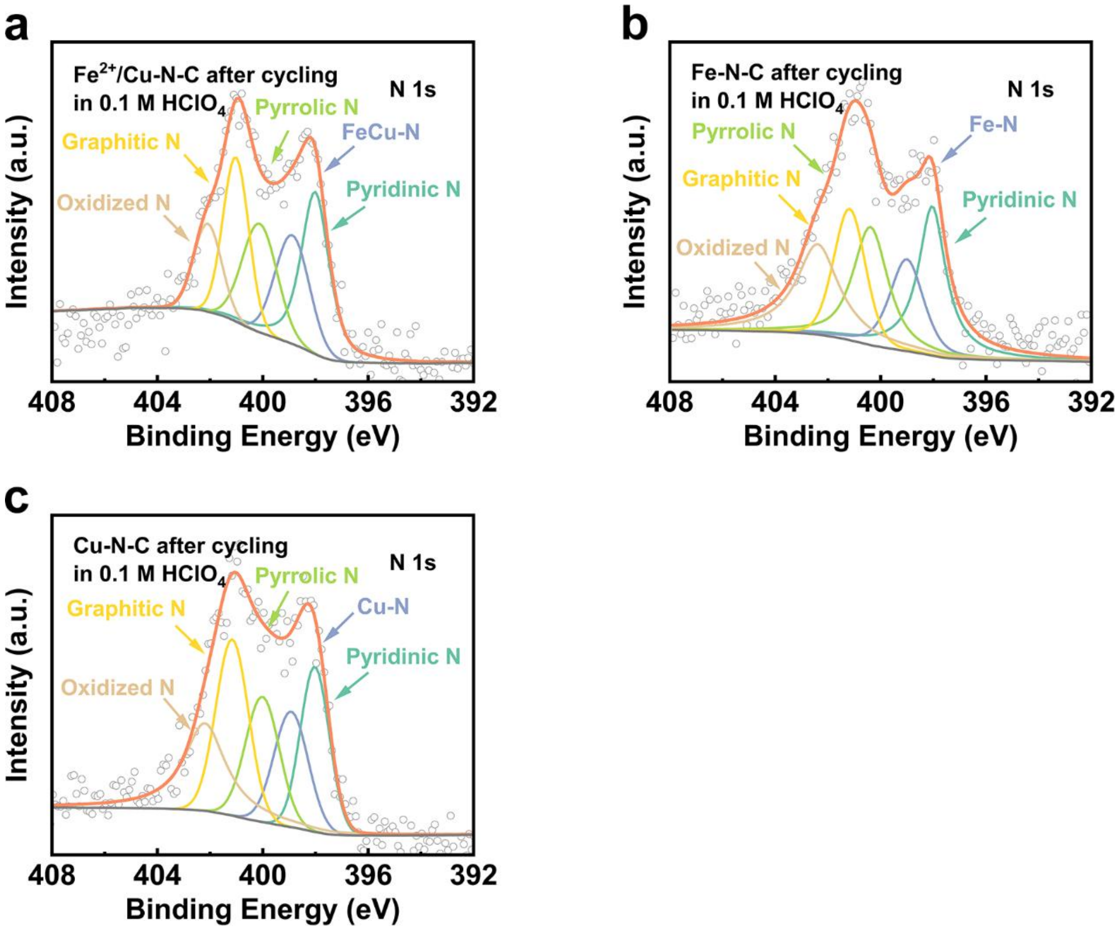

Fig. S38: High-resolution XPS spectra of N 1s after 10000 potential cycles for (a) Fe^{2+}/Cu-N-C, (b) Fe-N-C and (c) Cu-N-C.

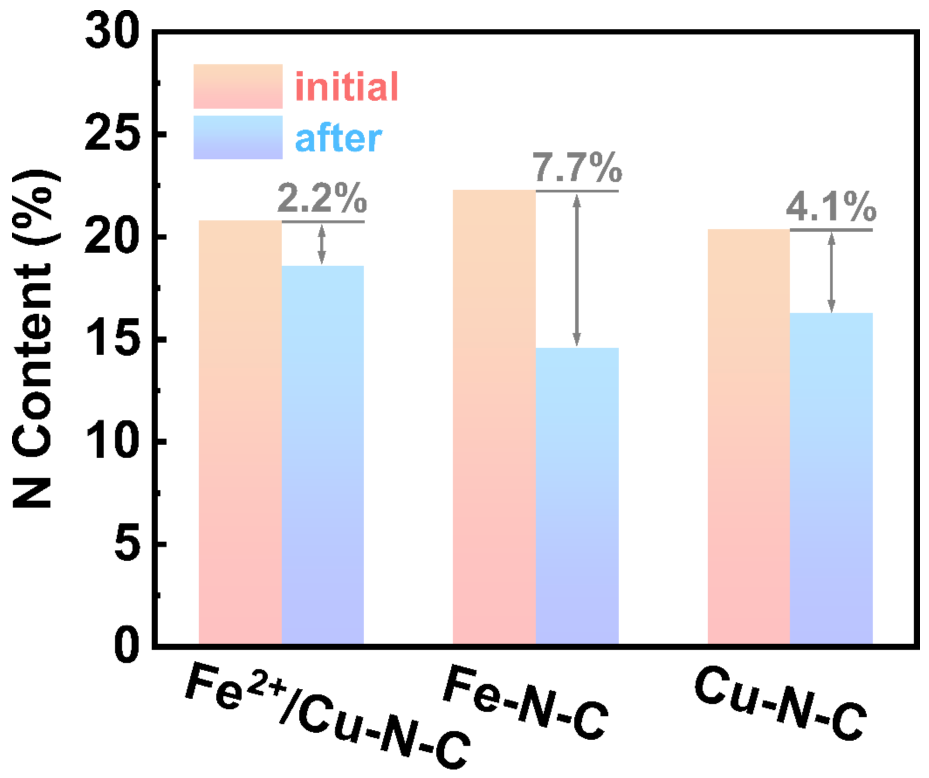

Fig. S39: The difference of M-N contents for Fe^{2+}/Cu-N-C, Fe-N-C and Cu-N-C in the initial and after the cycles.

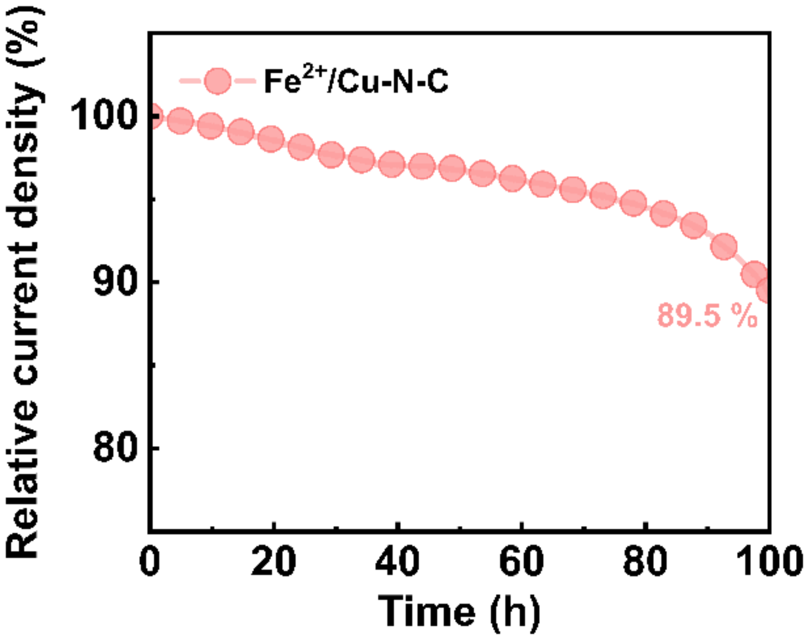

Fig. S40: Stability test of Fe^{2+}/Cu-N-C at a constant cell voltage of 0.6 V.

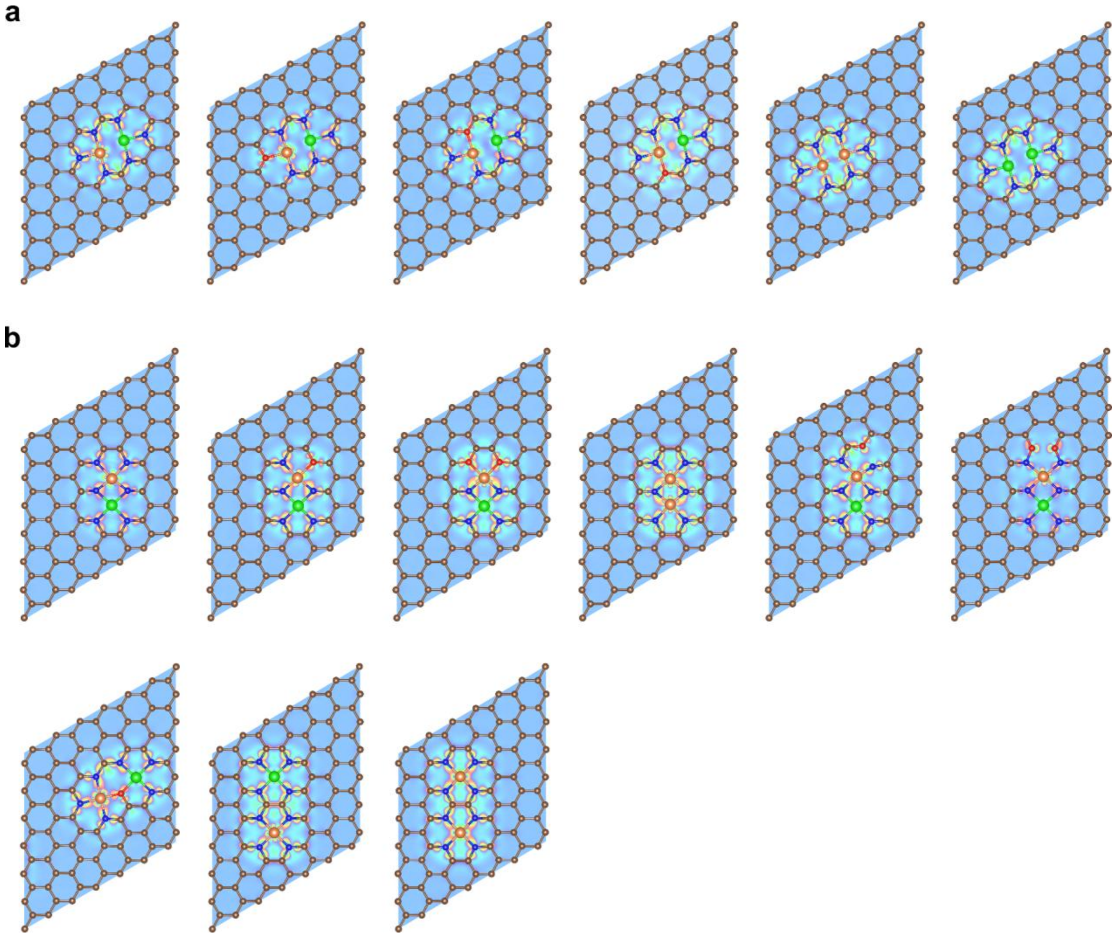

Fig. S41: The charge density difference of Fe/Cu diatomic models for (a) coordination sphere (CS) = 3 and (b) CS = 4. C: brown, N: blue, O: red, Fe: orange, Cu: green. The isosurface value is 0.002 e $bohr^{-3}$.

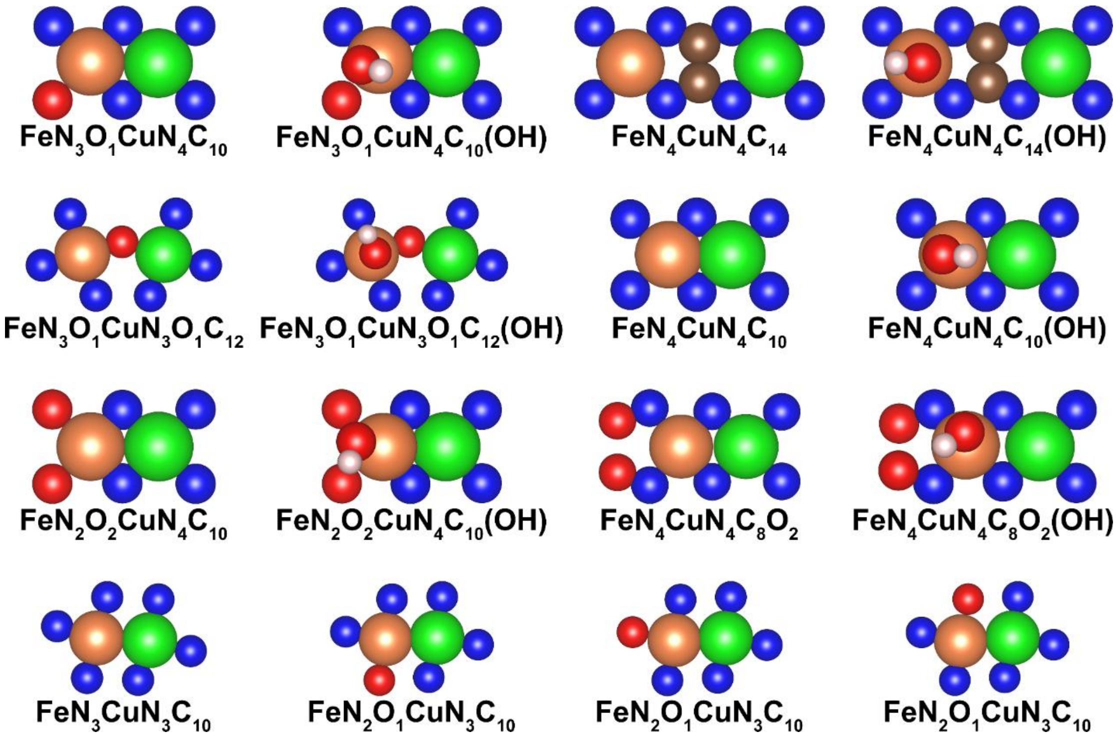

Fig. S42: The active centers of the Fe/Cu diatomic configurations with different coordination spheres (CS = 3 and 4).

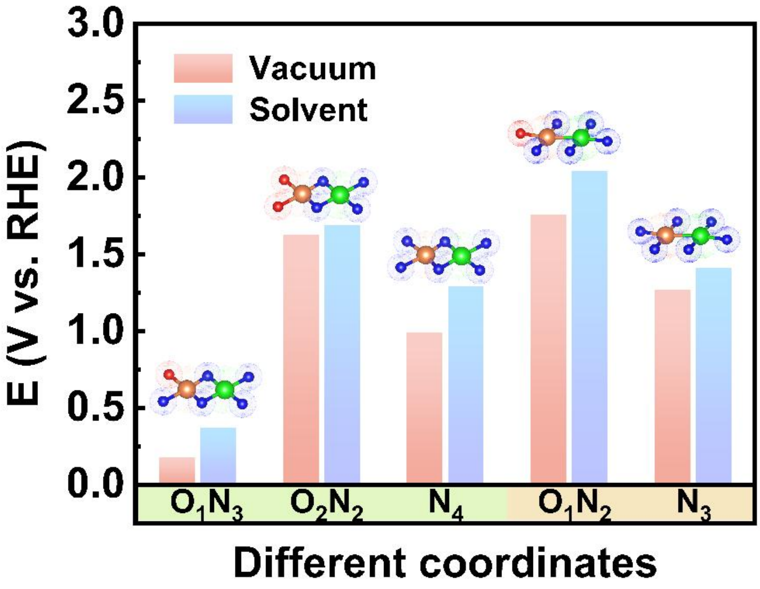

Fig. S43: Theoretical overpotential of different configurations for vacuum and solvent models, respectively.

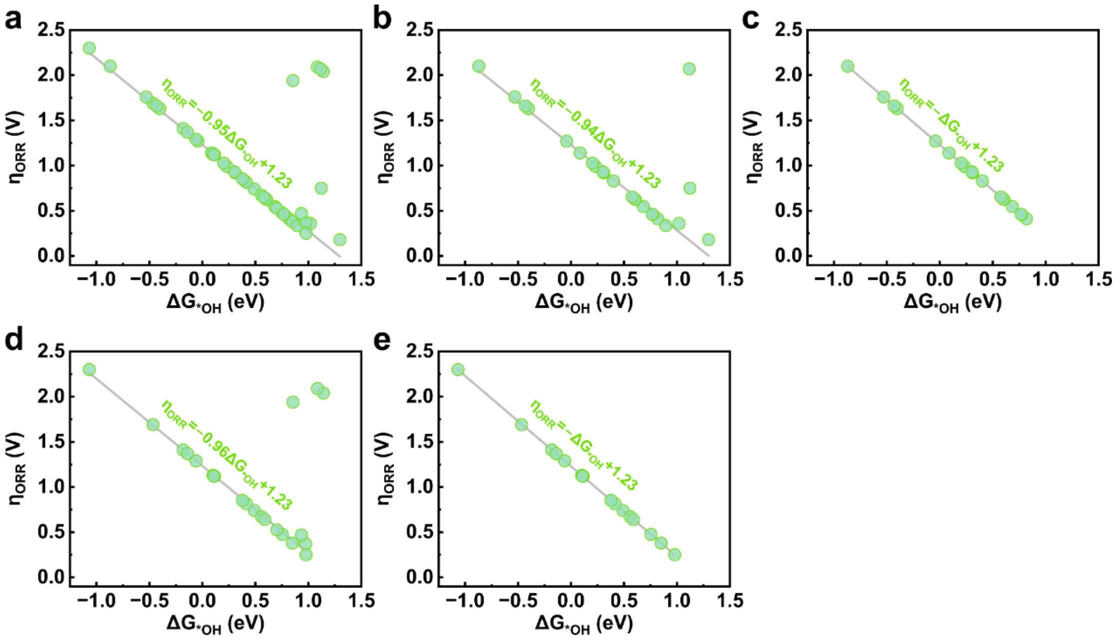

Fig. S44: The scaling relationships between ΔG_{*OH} and theoretical overpotentials η for (a) all the models we considered, (b) vacuum models, (c) vacuum models of hydroxyl modification, (d) solvent models and (e) solvent models of hydroxyl modification, respectively.

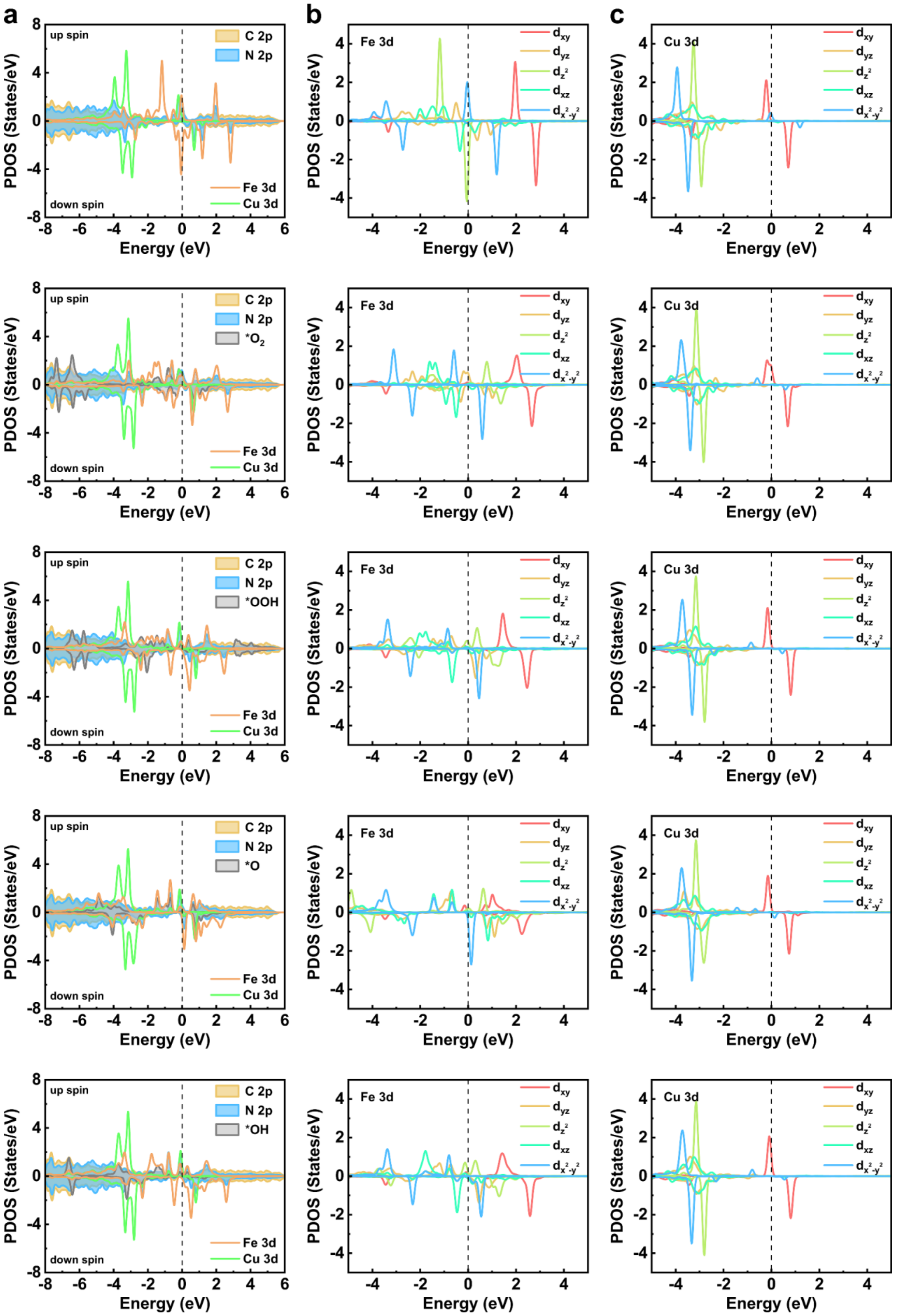

Fig. S45: The projected density of states (PDOS) for (a) $FeN_4CuN_4C_{10}$ and adsorption configurations models ($^*O_2$, $^*OOH$, $^*O$ and $^*OH$), (b) Fe 3d and (c) Cu 3d orbits.

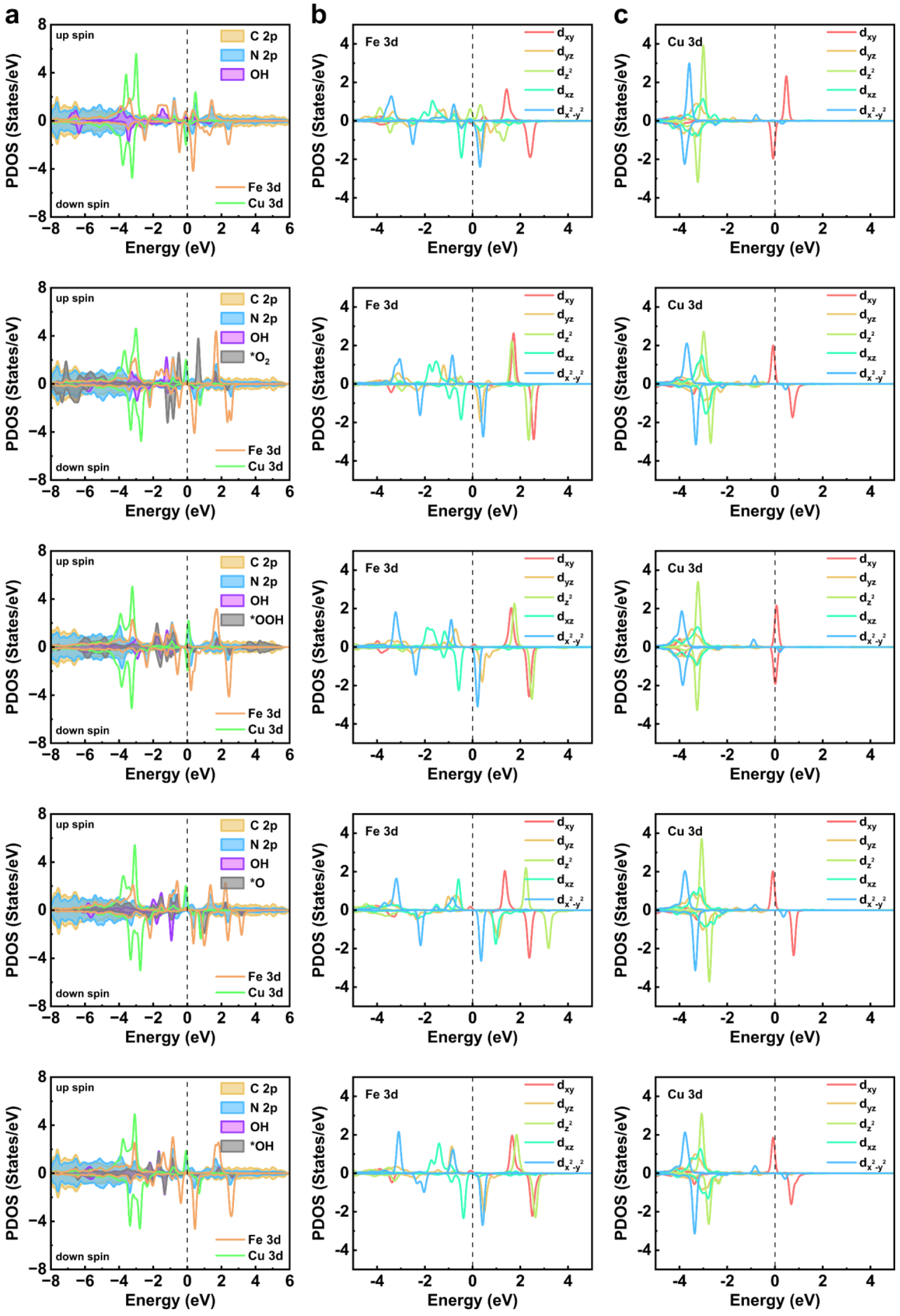

Fig. S46: The PDOS for (a) $FeN_4CuN_4C_{10}(OH)$ and adsorption configurations models ($^*O_2$, $^*OOH$, $^*O$ and $^*OH$), (b) Fe 3d and (c) Cu 3d orbits.

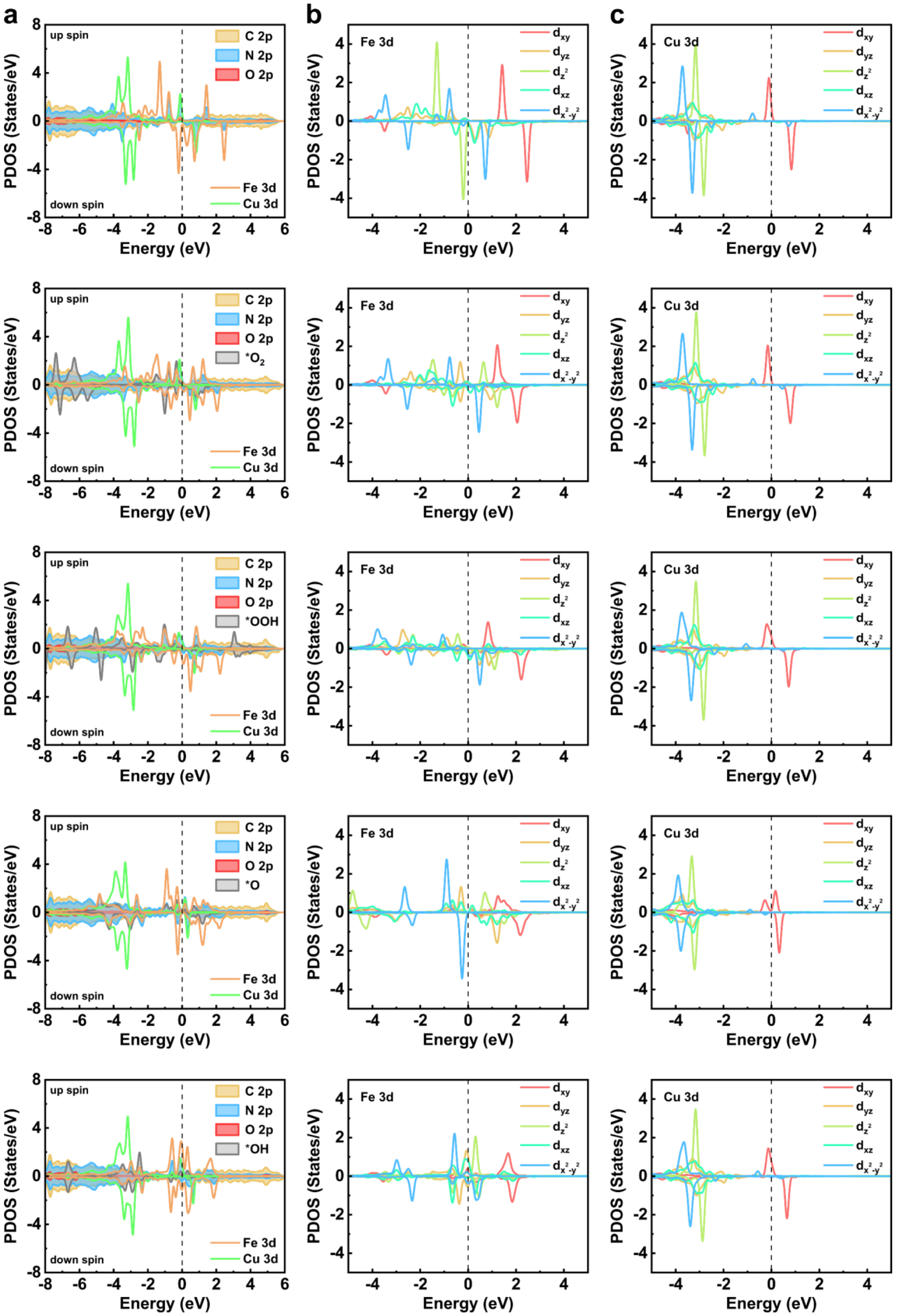

Fig. S47: The PDOS for (a) $FeN_3O_1CuN_4C_{10}$ and adsorption configurations models ($^*O_2$, $^*OOH$, $^*O$ and $^*OH$), (b) Fe 3d and (c) Cu 3d orbits.

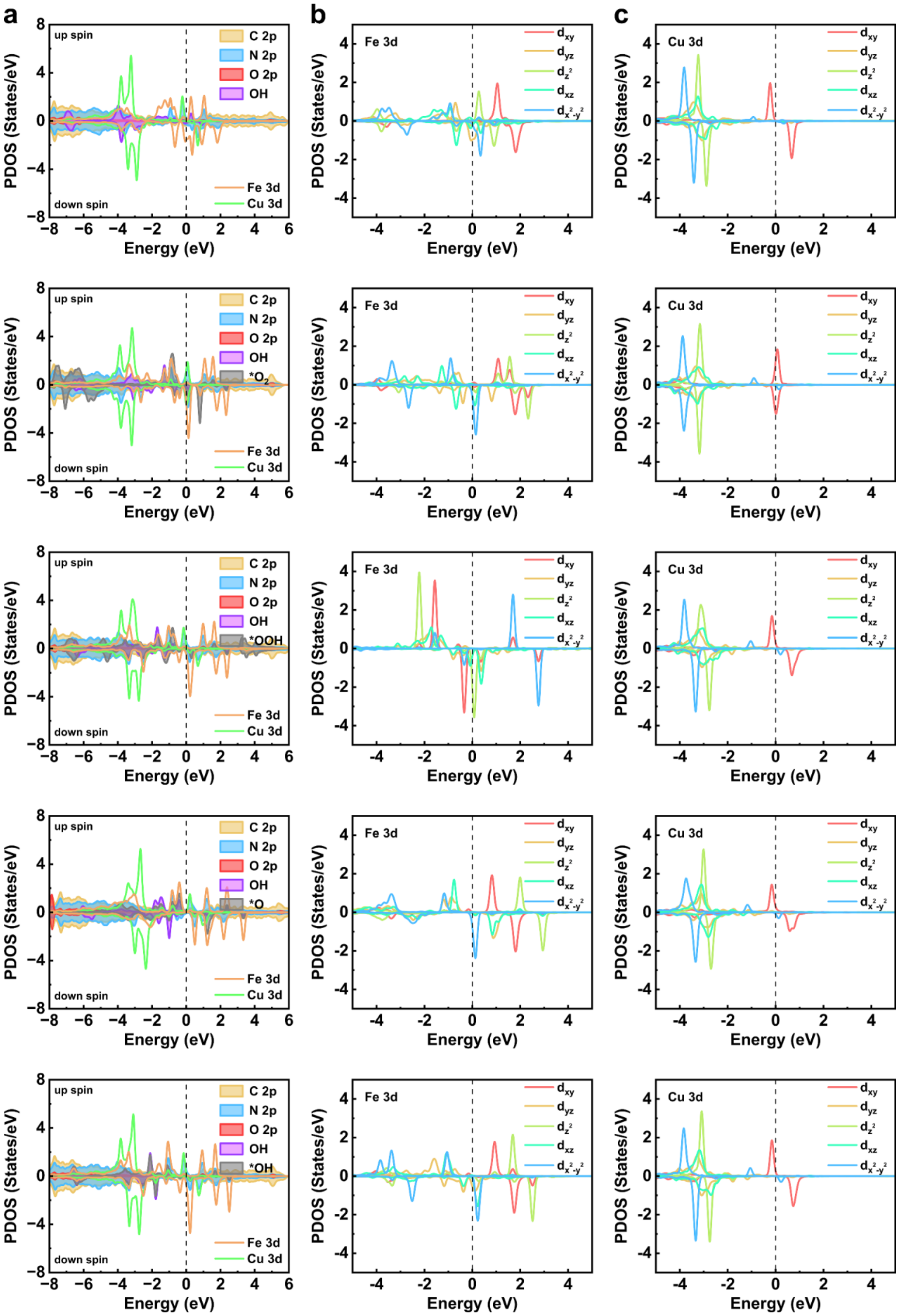

Fig. S48: The DOS for (a) $FeN_3O_1CuN_4C_{10}(OH)$ and adsorption configurations models ($^*O_2$, $^*OOH$, $^*O$ and $^*OH$), (b) Fe 3d and (c) Cu 3d orbits.

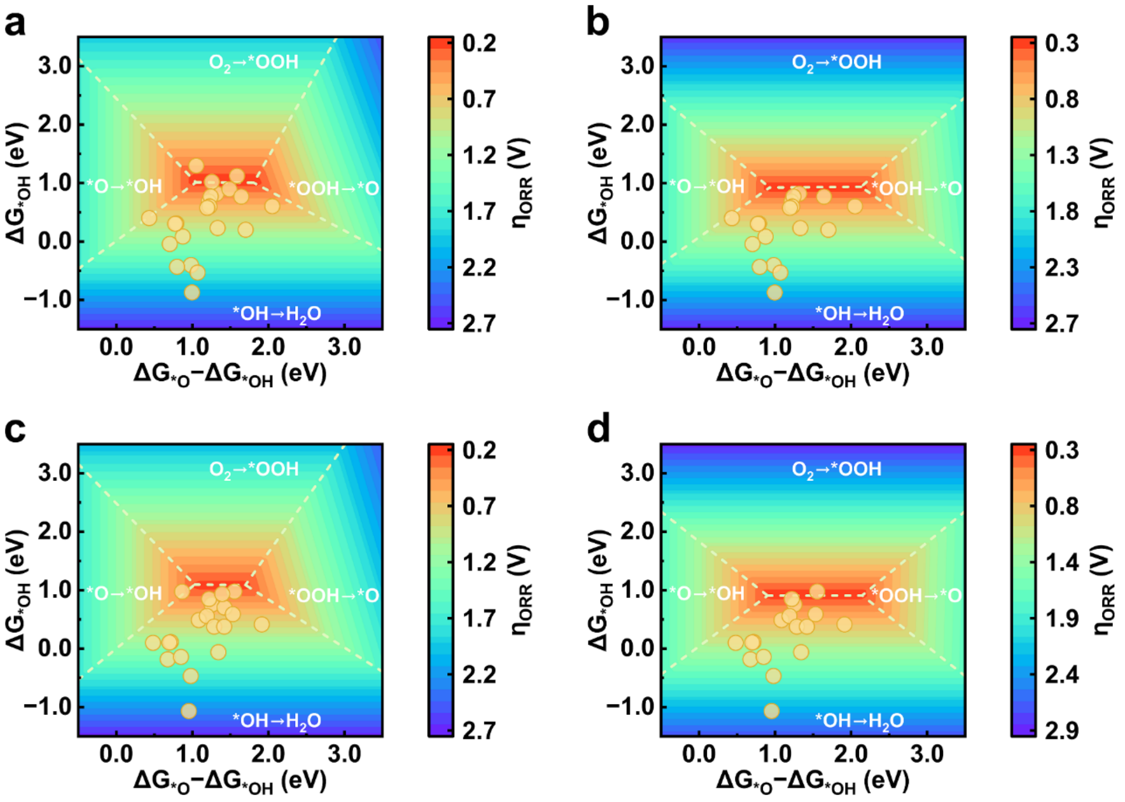

Fig. S49: Colored counter plots ORR activity volcanos for the diatomic catalysts by showing the ORR as a function of the Gibbs free energies for (a) vacuum models, (b) vacuum models of hydroxyl modification, (c) solvent models and (d) solvent models of hydroxyl modification, respectively.

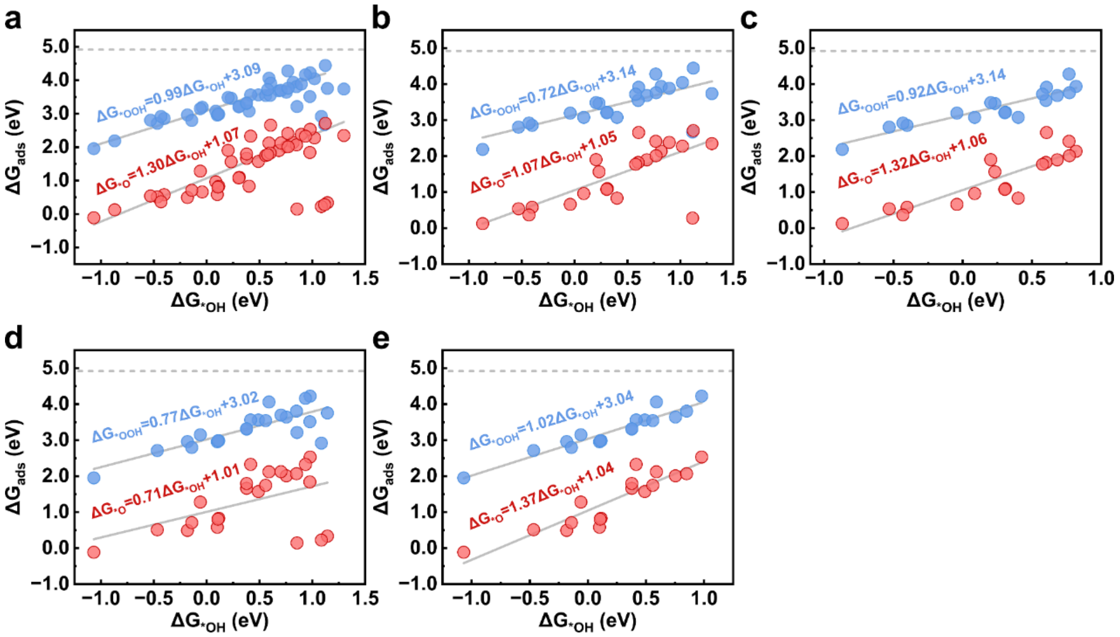

Fig. S50: Scaling relationships between adsorption free energies of intermediates (*OOH vs. *OH in blue sphere, O vs. OH in red sphere) on Fe/Cu diatomic catalysts for (a) all the models we considered, (b) vacuum models, (c) vacuum models of hydroxyl modification, (d) solvent models and (e) solvent models of hydroxyl modification, respectively. The dashed horizontal line represents the O_2 initial state at 4.92 eV.

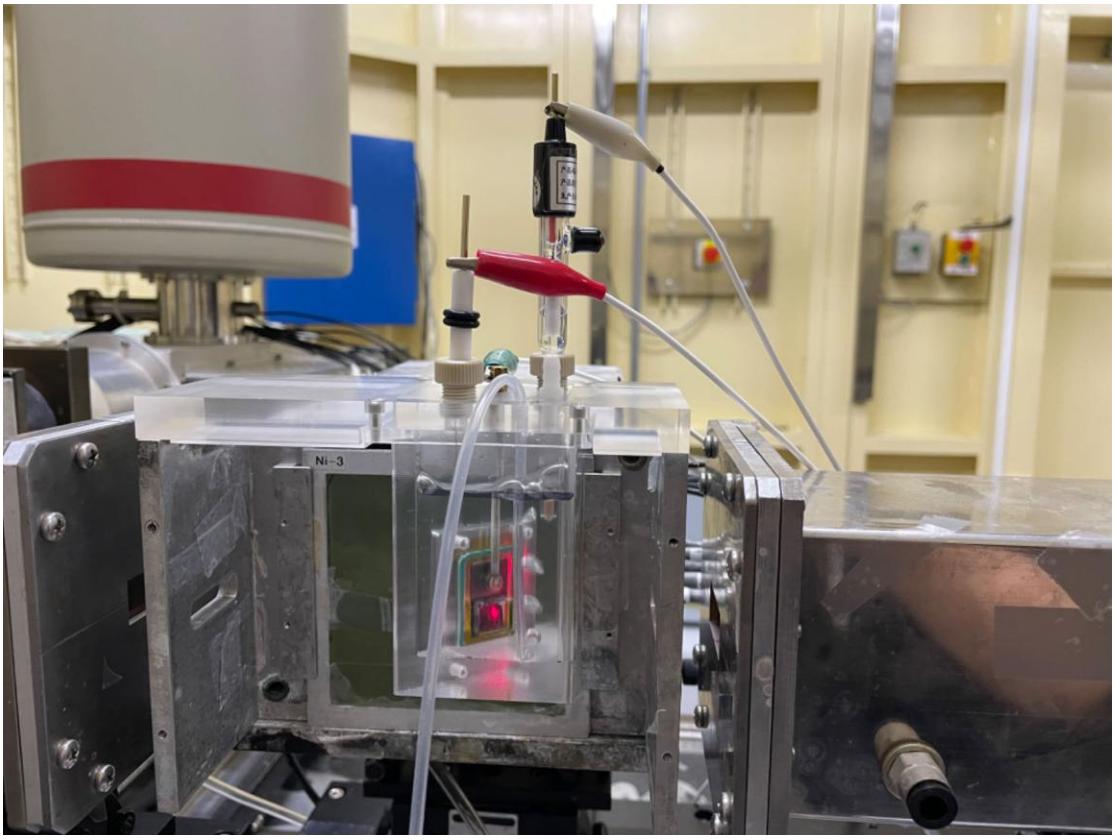

Fig. S51: In situ operando XAFS device constructed by three electrode system.

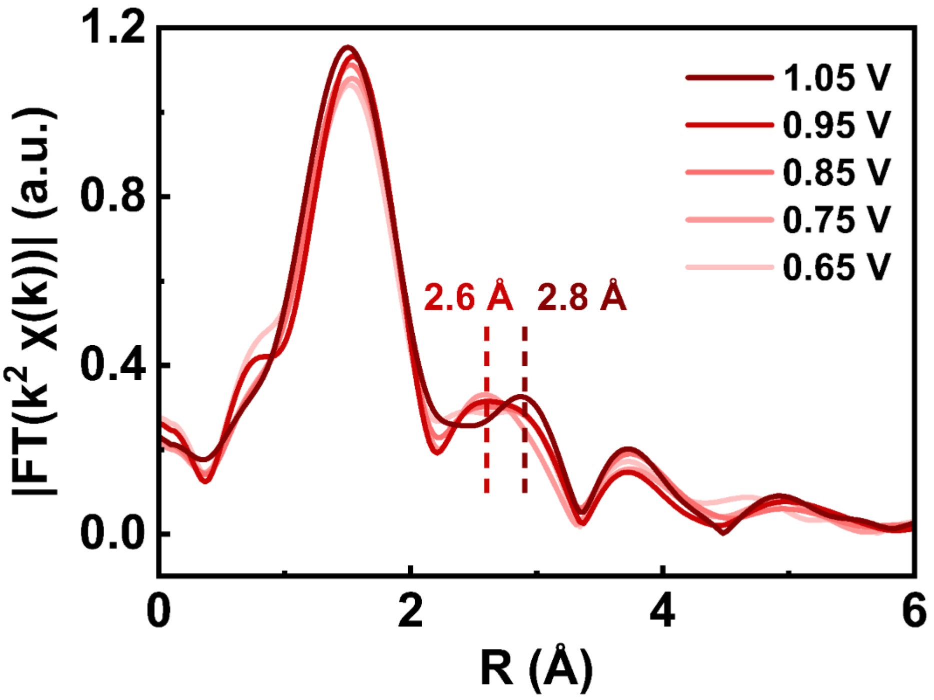

Fig. S52: In-situ FT-EXAFS spectra of Fe K-edge for Fe^{2+}/Cu-N-C under different applied potentials.

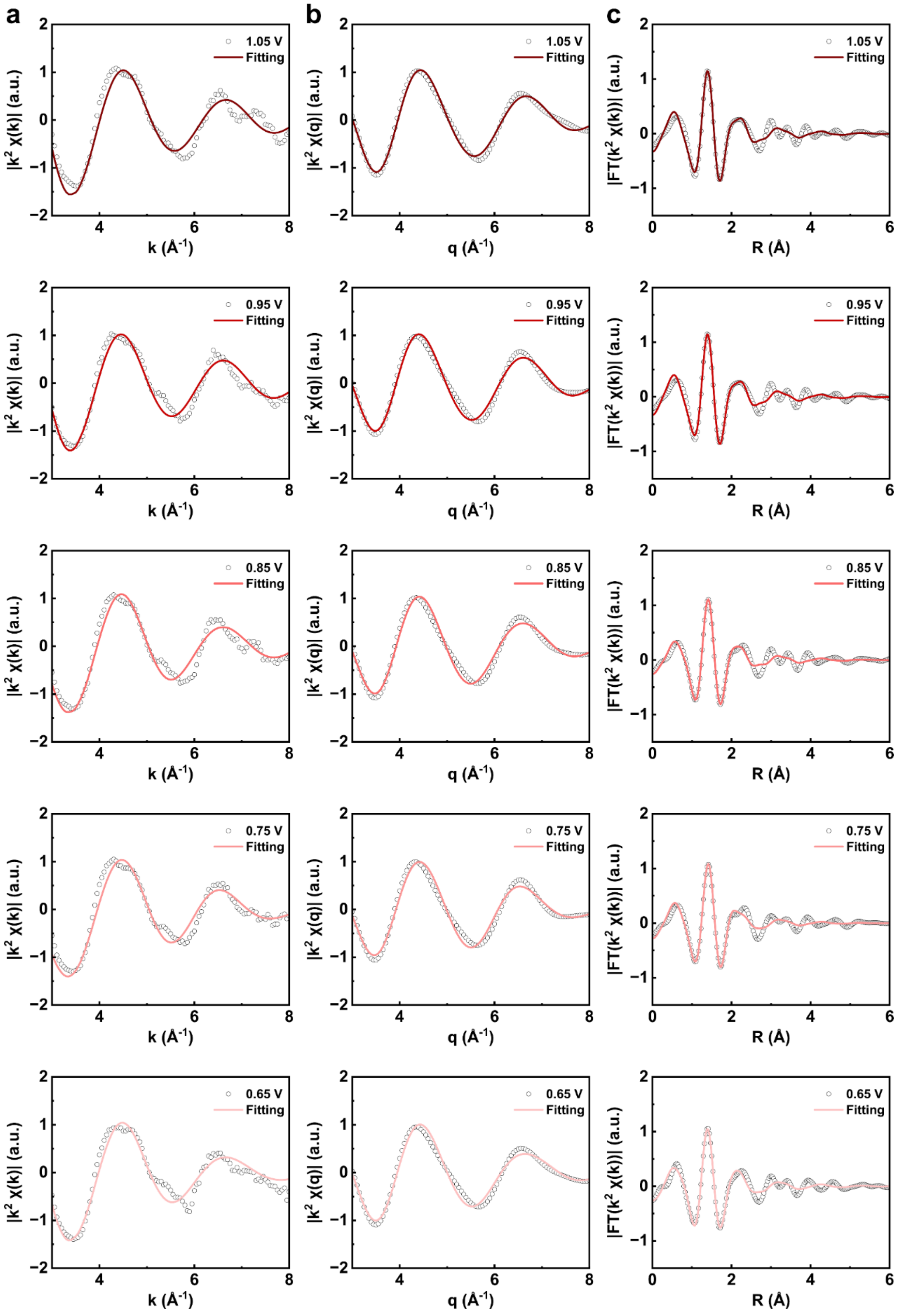

Fig. S53: In-situ FT-EXAFS spectra of Fe K-edge for Fe^{2+}/Cu-N-C under different applied potentials for (a) k-space, (b) q-space and (c) imaginary component of R-space.

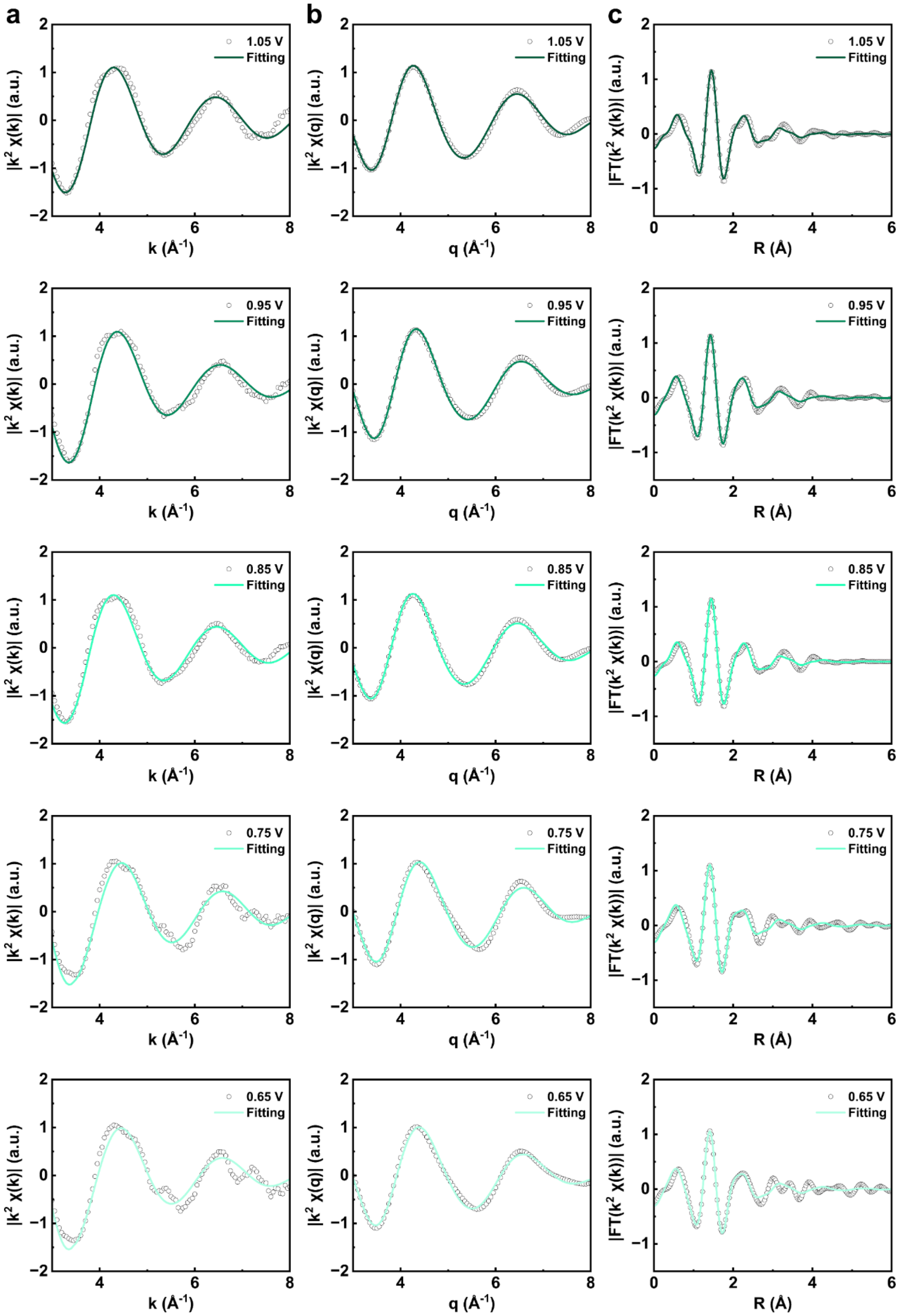

Fig. S54: In-situ FT-EXAFS spectra of Fe K-edge for Fe^{3+}/Cu-N-C under different applied potentials for (a) k-space, (b) q-space and (c) imaginary component of R-space.

Table S1. Structural parameters at the Fe and Cu K-edge for Fe^{2+}/Cu-N-C and Fe^{3+}/Cu-N-C catalysts extracted from quantitative EXAFS curve fitting using the Artemis.

Sample	Path	CN	R (Å)	σ^2 ($Å^2$)	ΔE_0 (eV)	R-factor
$^{a}Fe^{2+}$/Cu-N-C	Fe-N	4.1	1.97	0.0143	7.8	0.025
$^{b}Fe^{2+}$/Cu-N-C	Fe-N	3.2	1.95	0.0118	8.9	0.016
	Fe-O	0.95	1.87	0.0082		
Fe^{3+}/Cu-N-C	Fe-N	4.2	1.95	0.0134	8.8	0.057
Fe^{2+}/Cu-N-C	Cu-N	4.3	1.89	0.0080	9.1	0.066
Fe^{3+}/Cu-N-C	Cu-N	4.1	1.95	0.0072	7.4	0.071

a and b represent single scattering path and multipath scattering fitting, respectively. Multiple scattering path fitting shows a lower R-factor. CN: coordination number; R: the distance between the absorber and the backscattered atom; σ^2: Debye-Waller factor accounts for both thermal and structural disturbances; ΔE_0: the internal potential correction to compensate the internal potential differences between the sample and the reference compound; R-factor: the goodness of fitting.

Table S2. Comparison of ORR performances between Fe^{2+}/Cu-N-C and other reported carbon-based materials in 0.1 M KOH.

Catalysts	$E_{1/2}$ (V)	J_L (mA cm^{-2})	Journal/Year	Reference
Fe^{2+}/Cu-N-C	0.926	6.35	-	**This work**
Fe_2-N_6	0.920	~5.3	*Nat. Commun. (2025)*	[7]
CuNa-CF	0.890	~5.4	*Nat. Commun. (2024)*	[8]
FeCo-NCH	0.860	5.5	*Nat. Commun. (2023)*	[9]
$Co(CN)_3$-Cub	0.900	~5.5	*Nat. Catal. (2023)*	[10]
HESA	0.870	5.8	*Nat. Sustain. (2023)*	[11]
FeNC-V_N	0.902	<6.0	*J. Am. Chem. Soc. (2024)*	[12]
Fe_2N_6-S	0.921	5.6	*Adv. Mater. (2024)*	[13]
NiFe-N-C	0.870	~6.0	*Energy Environ. Sci. (2024)*	[14]
NCPor-Co	0.830	5.35	*Angew. Chem. Int. Ed. (2024)*	[15]
ZnCoFe-TAC/SNC	0.901	~5.2	*Energy Environ. Sci. (2024)*	[16]
Fe–N_4SP/NPS-HC	0.912	~5.5	*Energy Environ. Sci. (2024)*	[17]
$Fe_{SA/AC}$@HNC	0.900	~6.0	*Adv. Mater. (2024)*	[18]
Mn-SAS	0.850	6.2	*Angew. Chem. Int. Ed. (2023)*	[19]
Co SA-NDGs	0.850	<6.0	*Nat. Commun. (2022)*	[20]
Cu-N-C/GC	0.840	6.2	*Angew. Chem. Int. Ed. (2022)*	[21]
Cu/Zn-NC	0.830	~5.8	*Angew. Chem. Int. Ed. (2021)*	[22]
Cu-N-C SAC	0.830	5.25	*J. Am. Chem. Soc. (2021)*	[23]

Table S3. Comparison of ORR activity and stability of Fe^{2+}/Cu-N-C and other reported carbon-based materials in acidic media.

Catalysts	$E_{1/2}$ (V)	Durability test $\Delta E_{1/2}$ (cycle)	Electrolyte	Journal/Year	Reference
Fe^{2+}/Cu-N-C	0.828	17 mV (10k)	0.1 M $HClO_4$	-	**This work**
Fe-N-C	0.815	-	0.5 M H_2SO_4	*Nat. Catal. (2022)*	[24]
$Fe\text{-}NC_{Phen}$	0.830	33 mV (10k)	0.1 M H_2SO_4	*Angew. Chem. Int. Ed. (2023)*	[25]
Fe_{SA}/Fe_{AC}-2DNPC	0.810	15 mV (10k)	0.5 M H_2SO_4	*Nat. Commun. (2022)*	[26]
$Fe_2\text{-}S_1N_5N_5$/SNC	0.829	26 mV (5k)	0.1 M $HClO_4$	*Energy Environ. Sci. (2024)*	[27]
N-HPCs	0.780	-	0.5 M H_2SO_4	*Angew. Chem. Int. Ed. (2022)*	[28]
FeCo-N-HCN	0.750	-	0.1 M $HClO_4$	*Adv. Funct. Mater. (2021)*	[29]
Fe_x/Cu-N@CF	0.815	-	0.1 M $HClO_4$	*Energy Environ. Sci., (2023)*	[30]
$FeMn_{ac}/Mn\text{-}N_4C$	0.790	-	0.5 M H_2SO_4	*Angew. Chem. Int. Ed. (2022)*	[31]
COP_{BTC}@ Cl-CNTs	0.751	-	0.1M $HClO_4$	*Angew. Chem. Int. Ed. (2023)*	[32]
ZnFe-N-C	0.795	-	0.1 M $HClO_4$	*Angew. Chem. Int. Ed. (2023)*	[33]

Table S4. M-N contents before and after CV cycling.

Sample	M-N content (initial)	M-N content (after)	Difference
Fe^{2+}/Cu-N-C	20.8%	18.6%	2.2%
Fe-N-C	22.3%	14.6%	7.7%
Cu-N-C	20.4%	16.3%	4.1%

Table S5. Residual metal amounts in electrolytes after CV cycling.

Sample	Fe (μg/L)	Cu (μg/L)
Electrolyte ($HClO_4$)	0	0
Fe-N-C (Titanium mesh)	4274.0	-
Fe-N-C (Titanium mesh)	4277.4	-
Cu-N-C (Titanium mesh)	-	6042.3
Cu-N-C (Titanium mesh)	-	6050.5
Fe^{2+}/Cu-N-C (Titanium mesh)	2576.2	5334.1
Fe^{2+}/Cu-N-C (Titanium mesh)	2629.6	5272.8

Table S6. Comparison of PEMFC performance of Fe^{2+}/Cu-N-C with other reported M-N-C catalysts.

Catalysts	Loading (mg cm^{-2})	H_2-O_2, P_{max} (W cm^{-2})	H_2-air, P_{max} (W cm^{-2})	Reference
Fe^{2+}/Cu-N-C	2	0.95	0.49	**This work**
FeN_x/GM	4.0	0.86	0.43	[34]
Fe/NC-NaCl	4.0	0.89	0.39	[35]
FeN_4/HOPC-c-1000	4.0	0.68	0.42	[36]
0.17CVD/Fe-N-C-kat	4.0	0.70	0.32	[37]
Fe SAC-MOF-5	4.0	0.84	0.31	[38]
20Co-NC-1100	4.0	0.56	0.28	[39]
Co-N-PCNFs	4.0	0.71	0.40	[40]
20Mn-NC-second	4.0	0.46	0.17	[41]
Fe SAs/N-C	-	0.68	0.35	[42]
1.5Fe-ZIF	4.0	0.67	0.36	[43]
(CM+PANI)-Fe-C	4.0	0.87	0.42	[44]
(Fe,Co)/N-C	0.77	0.85	0.50	[45]
Fe_2N_6	4.0	0.85	0.36	[46]
sur-FeN_4-HPC	4.0	0.79	0.41	[47]
ZIF-NC-0.5Fe700	3.5	0.73	0.29	[48]
Spa-S-Fe,Co/NC	2.5	0.66	0.29	[49]
Fe_{SA}/Fe_{AC}-2DNPC	1.8	0.80	0.34	[26]

Table S7. ΔG of 3-coordination models for each intermediate and overpotential.

Models	Status	ΔG_{*O_2} (eV)	ΔG_{*OOH} (eV)	ΔG_{*O} (eV)	ΔG_{*OH} (eV)	η (V)
$FeN_3CuN_3C_{10}$	vacuum	1.73	2.53	0.70	-0.04	1.27
	solvation	1.96	2.47	0.68	-0.18	1.41
$FeN_2O_1CuN_3C_{10}$_1	vacuum	2.11	2.27	1.07	-0.53	1.76
	solvation	1.16	3.42	-0.81	1.14	2.04
$FeN_2O_1CuN_3C_{10}$_2	vacuum	2.00	2.55	0.80	-0.43	1.66
	solvation	2.19	2.50	-0.86	1.09	2.09
$FeN_2O_1CuN_3C_{10}$_3	vacuum	2.25	2.39	-0.84	1.12	2.07
	solvation	1.71	3.07	-0.71	0.85	1.94
$CuN_3CuN_3C_{10}$	vacuum	0.48	1.73	1.59	1.12	0.75
	solvation	0.76	1.84	1.39	0.94	0.43

Table S8. ΔG of 4-coordination models for each intermediate and overpotential.

Models	Status	ΔG_{*O_2} (eV)	ΔG_{*OOH} (eV)	ΔG_{*O} (eV)	ΔG_{*OH} (eV)	η (V)
$FeN_4CuN_4C_{10}$	vacuum	1.46	1.90	1.33	0.23	1.00
	solvation	1.77	1.87	1.34	-0.06	1.29
$FeN_3O_1CuN_4C_{10}$	vacuum	1.18	1.39	1.05	1.30	0.18
	solvation	1.41	1.67	0.86	0.98	0.37
$FeN_2O_2CuN_4C_{10}$	vacuum	2.07	2.28	0.98	-0.40	1.63
	solvation	2.21	2.20	0.98	-0.47	1.70
$FeN_4FeN_4C_{10}$	vacuum	0.87	1.77	1.26	1.02	0.36
	solvation	1.11	1.74	1.22	0.85	0.38
$FeN_4CuN_4C_9O_1$	vacuum	1.84	2.12	0.87	0.09	1.14
	solvation	2.12	2.09	0.85	-0.14	1.37
$FeN_4CuN_4C_8O_2$	vacuum	2.73	2.06	0.99	-0.87	2.10
	solvation	2.97	2.07	0.95	-1.07	2.30
$FeN_3O_1CuN_3O_1C_{10}$	vacuum	1.84	2.24	0.43	0.40	0.83
	solvation	1.96	2.37	0.48	0.10	1.13
$FeN_4CuN_4C_{14}$	vacuum	1.70	2.12	0.79	0.31	0.92
	solvation	1.94	2.15	0.72	0.11	1.12
$FeN_4FeN_4C_{14}$	vacuum	1.71	2.13	0.77	0.30	0.93
	solvation	1.94	2.18	0.70	0.11	1.12

Table S9. ΔG of hydroxyl ligand 4-coordination models for each intermediate and overpotential.

Models	Status	ΔG_{*O_2} (eV)	ΔG_{*OOH} (eV)	ΔG_{*O} (eV)	ΔG_{*OH} (eV)	η (V)
$FeN_4CuN_4C_{10}(OH)$	vacuum	0.98	1.80	1.32	0.82	0.41
	solvation	1.27	1.64	1.26	0.75	0.48
$FeN_3O_1CuN_4C_{10}(OH)$	vacuum	1.16	1.75	1.24	0.77	0.46
	solvation	1.38	1.80	1.19	0.56	0.67
$FeN_2O_2CuN_4C_{10}(OH)$	vacuum	0.64	1.87	1.64	0.77	0.46
	solvation	0.86	1.94	1.53	0.59	0.64
$FeN_4FeN_4C_{10}(OH)$	vacuum	1.03	1.50	1.49	0.90	0.33
	solvation	1.22	1.57	1.42	0.70	0.53
$FeN_4CuN_4C_9O_1(OH)$	vacuum	1.44	1.58	1.70	0.20	1.03
	solvation	0.70	1.69	1.55	0.98	0.25
$FeN_4CuN_4C_8O_2(OH)$	vacuum	1.01	1.26	2.05	0.60	0.62
	solvation	1.36	1.23	1.91	0.42	0.81
$FeN_3O_1CuN_3O_1C_{10}(OH)$	vacuum	1.24	1.79	1.21	0.68	0.55
	solvation	1.36	1.99	1.08	0.49	0.74
$FeN_4CuN_4C_{14}(OH)$	vacuum	1.37	1.72	1.22	0.60	0.63
	solvation	1.61	1.65	1.29	0.38	0.85
$FeN_4FeN_4C_{14}(OH)$	vacuum	1.20	1.95	1.20	0.58	0.65
	solvation	1.60	1.52	1.42	0.38	0.85

Table S10. Fe^{2+}/Cu-N-C structure parameters extracted by EXAFS at in-situ Fe K-edge under different applied potentials.

Potential	Path	CN	R (Å)	σ^2 ($Å^2$)	ΔE_0 (eV)	R-factor
1.05 V	Fe-N	3.1	1.97	0.0135	5.8	0.027
	Fe-O	1.0	1.87			
0.95 V	Fe-N	2.9	1.98	0.0085	5.9	0.029
	Fe-O	2.0	1.85			
0.85 V	Fe-O	3.0	1.98	0.0077	4.2	0.025
	Fe-O	2.1	1.84			
0.75 V	Fe-N	3.0	2.01	0.0424	7.1	0.034
	Fe-O	2.0	1.86			
0.65 V	Fe-N	3.0	1.98	0.0119	6.5	0.013
	Fe-O	2.1	1.85			

The total coordination number of Fe sites varied by 4.1(0.1), 4.9(0.2), 5.1(0.2), 5(0.1) and 5.1(0.1), respectively within the voltage range of 1.05 to 0.65V. The error is depicted in the figure as confidence bands.

Table S11. Fe^{3+}/Cu-N-C structure parameters extracted by EXAFS at in-situ Fe K-edge under different applied potentials.

Potential	Path	CN	R (Å)	σ^2 (Å^2)	ΔE_0 (eV)	R-factor
1.05 V	Fe-N	4.1	1.95	0.0062	9.7	0.0017
0.95 V	Fe-N	4.1	1.94	0.0102	8.6	0.0011
0.85 V	Fe-N	4.2	1.95	0.0086	4.7	0.0012
0.75 V	Fe-N	4.0	1.98	0.0052	6.9	0.0029
	Fe-O	0.8	1.83	0.0093		
0.65 V	Fe-N	4.0	1.97	0.0015	6.5	0.0018
	Fe-O	0.9	1.84	0.0043		

The total coordination number of Fe sites varied by 4.1(0.2), 4.1(0.2), 4.2(0.1), 4.8(0.3) and 4.9(0.2), respectively within the voltage range of 1.05 to 0.65V. The error is depicted in the figure as confidence bands.

References

1. Liang Y, Li Y, Wang H *et al.* Co_3O_4 nanocrystals on graphene as a synergistic catalyst for oxygen reduction reaction. *Nat Mater* 2011; **10**: 780-6.

2. Kresse G, Furthmüller J. Efficiency of ab-initio total energy calculations for metals and semiconductors using a plane-wave basis set. *Comp Mater Sci* 1996; **6**: 15-50.

3. Kresse G, Furthmüller J. Efficient iterative schemes for ab initio total-energy calculations using a plane-wave basis set. *Phys Rev B* 1996; **54**: 11169-86.

4. Blöchl PE. Projector augmented-wave method. *Phys Rev B* 1994; **50**: 17953-79.

5. Perdew JP, Burke K, Ernzerhof M. Generalized gradient approximation made simple. *phys Rev Lett* 1997; **78**: 1396-6.

6. Martyna GJ, Klein ML, Tuckerman M. Nosé-Hoover chains: The canonical ensemble via continuous dynamics. *J Chem Phys* 1992; **97**: 2635-43.

7. Yan Y, Yu R, Liu M *et al.* General synthesis of neighboring dual-atomic sites with a specific pre-designed distance via an interfacial-fixing strategy. *Nat Commun* 2025; **16**: 334.

8. Li Y, Huang A, Zhou L *et al.* Main-group element-boosted oxygen electrocatalysis of Cu-N-C sites for zinc-air battery with cycling over 5000 h. *Nat Commun* 2024; **15**: 8365.

9. Jiang Z, Liu X, Liu X-Z *et al.* Interfacial assembly of binary atomic metal-N_x sites for high-performance energy devices. *Nat Commun* 2023; **14**: 1822.

10. Sun K, Dong J, Sun H *et al.* $Co(CN)_3$ catalysts with well-defined coordination structure for the oxygen reduction reaction. *Nat Catal* 2023; **6**: 1164-73.

11. Lei X, Tang Q, Zheng Y *et al.* High-entropy single-atom activated carbon catalysts for sustainable oxygen electrocatalysis. *Nat Sustain* 2023; **6**: 816.

12. Lyu L, Hu X, Lee S *et al.* Oxygen reduction kinetics of Fe-N-C single atom catalysts boosted by pyridinic N vacancy for temperature-adaptive Zn-air batteries. *J Am Chem Soc* 2024; **146**: 4803-13.

13. Liu M, Wang X, Cao S *et al.* Ferredoxin-inspired design of S-synergized Fe-Fe dual-metal center catalysts for enhanced electrocatalytic oxygen reduction reaction. *Adv Mater* 2024; **36**: 2309231.

14. Meng H, Wu B, Zhang D *et al.* Optimizing electronic synergy of atomically dispersed dual-metal Ni-N_4 and Fe-N_4 sites with adjacent Fe nanoclusters for high-efficiency oxygen electrocatalysis. *Energy Environ Sci* 2024; **17**: 704-16.

15. Huang S, Tranca D, Rodríguez-Hernández F *et al.* Well-defined N_3Cl-anchored single-metal-sites for oxygen reduction reaction. *Angew Chem Int Ed* 2024; **63**: e202314833.

16. Chen C, Chai J, Sun M *et al.* An asymmetrically coordinated ZnCoFe hetero-trimetallic atom catalyst enhances the electrocatalytic oxygen reaction. *Energy Environ Sci* 2024; **17**: 2298-308.

17. Liu J, Chen W, Yuan S *et al.* High-coordination Fe-N_4SP single-atom catalysts via the multi-shell synergistic effect for the enhanced oxygen reduction reaction of rechargeable Zn-air battery cathodes. *Energy Environ Sci* 2024; **17**: 249-59.

18. Zhang H, Chen H-C, Feizpoor S *et al.* Tailoring oxygen reduction reaction kinetics of Fe-N-C catalyst via spin manipulation for efficient zinc-air batteries. *Adv Mater* 2024; **36**: 2400523.

19. Tong M, Sun F, Xing G *et al.* Potential dominates structural recombination of single atom Mn sites for promoting oxygen reduction reaction. *Angew Chem Int Ed* 2023; **62**: e202314933.

20. Wang Q, Feng Q, Lei Y *et al.* Quasi-solid-state Zn-air batteries with an atomically dispersed cobalt electrocatalyst and organohydrogel electrolyte. *Nat Commun* 2022; **13**: 3689.

21. Xing G, Tong M, Yu P *et al.* Reconstruction of highly dense Cu-N_4 active sites in electrocatalytic oxygen reduction characterized by operando synchrotron radiation. *Angew Chem Int Ed* 2022; **61**: e202211098.

22. Tong M, Sun F, Xie Y *et al.* Operando cooperated catalytic mechanism of atomically dispersed Cu-N_4 and Zn-N_4 for promoting oxygen reduction reaction. *Angew Chem Int Ed* 2021; **60**: 14005-12.

23. Yang J, Liu W, Xu M *et al.* Dynamic behavior of single-atom catalysts in electrocatalysis: Identification of Cu-N_3 as an active site for the oxygen reduction reaction. *J Am Chem Soc* 2021; **143**: 14530-39.

24. Mehmood A, Gong M, Jaouen F *et al.* High loading of single atomic iron sites in Fe-NC oxygen reduction catalysts for proton exchange membrane fuel cells. *Nat Catal* 2022; **5**: 311-23.

25. Cheng X, Jiang X, Yin S *et al.* Instantaneous free radical scavenging by CeO_2 nanoparticles adjacent to the Fe-N_4 active sites for durable fuel cells. *Angew Chem Int Ed* 2023; **62**: e202306166.

26. Wan X, Liu Q, Liu J *et al.* Iron atom-cluster interactions increase activity and improve durability in Fe-N-C fuel cells. *Nat Commun* 2022; **13**: 2963.

27. Li Y, Luo X, Wei Z *et al.* Precisely constructing charge-asymmetric dual-atom Fe sites supported on hollow porous carbon spheres for efficient oxygen reduction. *Energy Environ Sci* 2024; **17**: 4646-57.

28. Kong F, Cui X, Huang Y *et al.* N-doped carbon electrocatalyst: Marked ORR activity in acidic media without the contribution from metal sites? *Angew Chem Int Ed* 2022; **61**: e202116290.

29. Li H, Wen Y, Jiang M *et al.* Understanding of neighboring Fe-N_4-C and Co-N_4-C dual active centers for oxygen reduction reaction. *Adv Funct Mater* 2021; **31**: 2011289.

30. Wu S, Jiang S, Liu S-Q *et al.* Single Cu-N_4 sites enable atomic Fe clusters with high-performance oxygen reduction reactions. *Energy Environ Sci* 2023; **16**: 3576-86.

31. Liu H, Jiang L, Khan J *et al.* Decorating single-atomic Mn sites with FeMn clusters to boost oxygen reduction reaction. *Angew Chem Inter Ed* 2023; **62**: e202214988.

32. Li X, Chen T, Yang B *et al.* Fundamental understanding of electronic structure in FeN_4 site on electrocatalytic activity via dz^2-orbital-driven charge tuning for acidic oxygen reduction. *Angew Chem Inter Ed* 2023; **62**: e202215441.

33. Shu X, Tan D, Wang Y *et al.* Bimetal-bridging nitrogen coordination in carbon-based electrocatalysts for pH-universal oxygen reduction. *Angew Chem Inter Ed* 2024; **63**: e202316005.

34. Fu X, Li N, Ren B *et al.* Tailoring FeN_4 sites with edge enrichment for boosted oxygen reduction performance in proton exchange membrane fuel cell. *Adv Energy Mater* 2019; **9**: 1803737.

35. Wang Q, Yang Y, Sun F *et al.* Molten NaCl-assisted synthesis of porous Fe-N-C electrocatalysts with a high density of catalytically accessible FeN_4 active sites and outstanding oxygen reduction reaction performance. *Adv Energy Mater* 2021; **11**: 2100219.

36. Qiao M, Wang Y, Wang Q *et al.* Hierarchically ordered porous carbon with atomically dispersed FeN_4 for ultraefficient oxygen reduction reaction in proton-exchange membrane fuel cells. *Angew Chem Int Ed* 2020; **59**: 2688-94.

37. Liu S, Wang M, Yang X *et al.* Chemical vapor deposition for atomically dispersed and nitrogen coordinated single metal site catalysts. *Angew Chem Int Ed* 2020; **59**: 21698-705.

38. Xie X, Shang L, Xiong X *et al.* Fe single-atom catalysts on MOF-5 derived carbon for efficient oxygen reduction reaction in proton exchange membrane fuel cells. *Adv Energy Mater* 2022; **12**: 2102688.

39. Wang XX, Cullen DA, Pan Y-T *et al.* Nitrogen-coordinated single cobalt atom catalysts for oxygen reduction in proton exchange membrane fuel cells. *Adv Mater* 2018; **30**: 1706758.

40. He Y, Guo H, Hwang S *et al.* Single cobalt sites dispersed in hierarchically porous nanofiber networks for durable and high-power PGM-free cathodes in fuel cells. *Adv Mater* 2020; **32**: 2003577.

41. Li J, Chen M, Cullen DA *et al.* Atomically dispersed manganese catalysts for oxygen reduction in proton-exchange membrane fuel cells. *Nat Catal* 2018; **1**: 935-45.

42. Yang Z, Wang Y, Zhu M *et al.* Boosting oxygen reduction catalysis with Fe-N_4 sites decorated porous carbons toward fuel cells. *ACS Catal* 2019; **9**: 2158-63.

43. Zhang H, Chung HT, Cullen DA *et al.* High-performance fuel cell cathodes exclusively containing atomically dispersed iron active sites. *Energy Environ Sci* 2019; **12**: 2548-58.

44. Chung HT, Cullen DA, Higgins D *et al.* Direct atomic-level insight into the active sites of a high-performance PGM-free ORR catalyst. *Science*. 2017; **357**: 479-84.

45. Wang J, Huang Z, Liu W *et al.* Design of N-coordinated dual-metal sites: A stable and active Pt-free catalyst for acidic oxygen reduction reaction. *J Am Chem Soc* 2017; **139**: 17281-84.

46. Zhang N, Zhou T, Ge J *et al.* High-density planar-like Fe_2N_6 structure catalyzes efficient oxygen reduction. *Matter*. 2020; **3**: 509-21.

47. Chen G, An Y, Liu S *et al.* Highly accessible and dense surface single metal FeN_4 active sites for promoting the oxygen reduction reaction. *Energy Environ Sci* 2022; **15**: 2619-28.

48. Li J, Zhang H, Samarakoon W *et al.* Thermally driven structure and performance evolution of atomically dispersed FeN_4 sites for oxygen reduction. *Angew Chem Int Ed* 2019; **58**: 18971-80.

49. Liu F, Shi L, Lin X *et al.* Fe/Co dual metal catalysts modulated by S-ligands for efficient acidic oxygen reduction in PEMFC. *Sci. Adv*. 2023; **9**: eadg0366.
